# Cyclic di-GMP directly reprograms the multidrug resistance machinery via UspG-mediated sequestration of RamR in *Klebsiella*

**DOI:** 10.1371/journal.ppat.1014537

**Published:** 2026-08-24

**Authors:** Xiaoxiao Liu, Mingfang Wang, Yikai Fu, Xiangjie Zhu, Miao Du, Yao Wen, Yiwen Liao, Yunhu Zhao, Yinyue Deng, Bing Gu

**Affiliations:** 1 Laboratory Medicine, Guangdong Provincial People’s Hospital, Guangdong Academy of Medical Sciences, Southern Medical University, Guangzhou, Guangdong, China; 2 Guangdong Cardiovascular Institute, Guangdong Provincial People’s Hospital, Guangdong Academy of Medical Science, Guangzhou, China; 3 School of Medicine, South China University of Technology, Guangzhou, Guangdong, China; 4 School of Pharmaceutical Sciences (Shenzhen), Shenzhen Campus of Sun Yat-sen University, Sun Yat-sen University, Shenzhen, China; University of Vermont, UNITED STATES OF AMERICA

## Abstract

The integration of environmental cues to counter selective pressures is crucial for the epidemiological success of major human pathogens. *Klebsiella pneumoniae* poses an increasing critical public health threat due to its high biofilm forming capacity and adaptive antimicrobial resistance. While the second messenger cyclic di-GMP (c-di-GMP) is a key regulator of bacterial cellular physiological adaptations, its downstream effectors that control antibiotic resistance remain unknown in *K. pneumoniae.* Unlike c-di-GMP metabolizing enzymes, which contain highly conserved GGDEF or EAL domains, effectors enable signal transduction through structurally heterogeneous sensing domains that defy homology-based prediction. Here, we identified that upon the elevation of intracellular c-di-GMP levels induced by antibiotic treatmen, the universal stress protein UspG (AVR78_17055) serves as a cryptic, direct c-di-GMP effector in extended-spectrum beta-lactamase (ESBL)-producing strain *K. quasipneumoniae* ATCC 700603. Utilizing site-directed mutagenesis and EMSA, we demonstrate that UspG senses elevated intracellular c-di-GMP levels, thereby promoting biofilm formation, via N39 and K116 residues. Mechanistically, c-di-GMP binding enhances the binding affinity of UspG to the transcriptional repressor RamR. This specific protein sequestration antagonizes RamR, a transcriptional repressor that regulates RamA expression, thereby derepressing the *ramA* locus and unleashing a regulatory program that fortifies lipid A biosynthesis, upregulates multidrug efflux pumps expression, and promotes biofilm development. Importantly, this c-di-GMP–UspG axis is not restricted to ESBL-producing lineages. Through mutagenesis verification, we discovered similar phenotypic dependency in hypervirulent *K. pneumoniae* ATCC 43816. These findings indicate that UspG is functionally conserved across *Enterobacteriaceae*. By elucidating how *Klebsiella* exploits UspG to bridge intracellular nucleotide signaling with acute environmental adaptation, our study provides a new therapeutic target for recalcitrant *Klebsiella* infections.

## Author summary

Multidrug-resistant *Klebsiella pneumoniae* represents a high-priority public threat challenge, driven by its capacity for robust biofilm formation and extensive antimicrobial resistance. However, the central regulatory mechanisms governing the transition of this pathogen from a susceptible to a resistant state remains to be elucidated. In this study, we discovered a previously missing molecular link in this bacterial defense strategy. We identified a universal stress protein, which we named UspG, that acts as a novel receptor for a widespread bacterial chemical messenger cyclic di-GMP in *Klebsiella*. Critically, our finding uncovers how bacteria utilize a conserved second-messenger system to rapidly modulate fixed genetic resistance elements—such as efflux pumps—in response to environmental cues. By bridging intracellular stress signaling with the RamA-mediated resistance regulon, this work uncovers a conserved survival mechanism in *Klebsiella* species that offers a strategic target for antimicrobial intervention. Unveiling this regulatory pathway not only advances our understanding of bacterial adaptation but also reveals a vulnerable target for designing innovative therapies against this priority pathogen in clinical environments.

## Introduction

*Klebsiella pneumoniae*, a prominent opportunistic pathogen of the *Enterobacteriaceae* family, accounts for one-fifth of all Gram-negative bacterial infections globally [1]. The escalating clinical threat of this pathogen is primarily driven by two divergent pathotypes: classical strains (cKp), which are frequently multidrug-resistant (MDR) and dominate hospital-acquired infections, and hypervirulent strains (hvKp), which cause severe tissue-invasive diseases in healthy individuals [2]. Despite their distinct clinical manifestations, a critical unifying factor in the therapeutic recalcitrance of both lineages is the pathogen’s intrinsic capacity to establish robust biofilms on biomedical devices [3]. Biofilms are Earth’s most ancient multicellular life forms, which develops when microbial cells transition from a single motile state to a surface-attached communities [4,5]. According to the NIH, more than 80% of recalcitrant infectious diseases are attributed to biofilms formed by pathogens [6]. Biofilms not only endow bacteria with a robust physical barrier against antimicrobial agents but also simultaneously providing a permissive niche for genetic exchange [7], thereby facilitating the horizontal transfer of resistance and virulence plasmids that drive the fusion and emergence of highly antibiotic resistant and hypervirulent strains of *K. pneumoniae* [8]. Consequently, the precise mechanistic interplay between biofilm development and elevated antimicrobial recalcitrance warrants thorough elucidation, particularly within the high-priority pathogen *K. pneumoniae* [9].

Although clinical studies frequently link MDR lineages, including extended-spectrum beta-lactamase (ESBL) -producing and polymyxin-resistant strains, to enhanced biofilm capacity, contradictory reports find no robust association between biofilm biomass and specific MDR phenotypes [10]. The phenotypic plasticity of biofilm is not merely a passive aggregation but a coordinated multicellular endeavor governed by molecular signaling [11]. In bacteria, the shift from motile state to a surface-attached communities constitutes the central dogma of cyclic di-GMP (c-di-GMP) signaling [12]. Typically, high intracellular c-di-GMP concentrations dictate a sessile bacterial lifestyle characterized by extracellular polymeric substances (EPS) production and biofilm formation, whereas low c-di-GMP pools favor motility and decreased biofilm formation [12–14]. However, it remains unresolved whether this resilient phenotype is primarily driven by the passive exclusion of antimicrobial agents via a fortified EPS matrix, or through the direct activation of active detoxification networks, such as efflux pumps, by specific c-di-GMP effectors [15]. Consequently, deciphering the intracellular regulatory networks that coordinate this lifestyle transition is critical for understanding how *K. pneumoniae* evades therapeutic intervention and establishes chronic infections [16].

In the prokaryote kingdom, c-di-GMP stands as the most profoundly studied cyclic dinucleotide (CDN) molecular [13]. CDNs have emerged as universal signaling motifs across the tree of life, translating discrete molecular binding events into macroscopic, collective physiological responses [17,18]. Unlike centralized mammalian signaling systems, bacteria have evolved a vast array of structurally heterogeneous effectors to transduce c-di-GMP into multifaceted physiological outputs [19–23]. While early studies characterized classical effectors, such as those containing dedicated PilZ domains [22,24,25], the ATPases of FleQ [26], two component systems [27], riboswitch motifs [28,29], and phosphodiesterases with enzymatically inactive GGDEF [30] or EAL/HD-GYP domains [31–33], recent advances have uncovered a class of non-canonical receptors that defy standard structural categorization. For instance, c-di-GMP binds the nucleoid-associated protein Lsr2 to modulate cell wall lipids in *Mycobacterium* [34], interacts with the global regulator H-NS to drive virulence gene expression [35], and regulates intestinal colonization via the WYL-domain transcription factor MbpR in *Lactiplantibacillus plantarum* [36]. Crucially, this structural heterogeneity highlights a fundamental challenge: unlike c-di-GMP metabolizing enzymes (diguanylate cyclase, DGCs and PDEs), which share easily recognizable GGDEF or EAL domains, these diverse receptors lack a unified consensus motif. This absence of sequence homology renders them recalcitrant to identification via standard bioinformatic prediction. Consequently, despite the clear role of c-di-GMP in initiating collective survival behaviors, the specific effectors that sense this signal and translate it into antimicrobial resistance remain largely elusive in *K. pneumoniae*.

Universal stress proteins (Usps) constitute an ancient superfamily of widespread cellular adaptors [37–39] across bacteria, archaea, and plants [40,41], that are essential for bacterial survival under diverse environmental pressures, including antibiotic resistance, adhesion, and motility [39,42]. While pioneering biochemical studies have linked Usp scaffolds to cAMP or c-di-AMP signaling to modulate basic metabolic homeostasis, e.g., potassium transport in *S. aureus* [43] or general viability in *Mycobacterium* [44], their potential integration into the c-di-GMP-driven regulatory networks of Gram-negative pathogens remains entirely obscure. Within this family, the universal stress protein G (UspG) emerges as a highly compelling yet mechanistically uncharacterized candidate [38]. Specifically, it remains elusive how UspG might perceive intracellular c-di-GMP fluxes to subsequently orchestrate downstream biofilm architecture and adaptive antimicrobial tolerance in *Klebsiella*.

Here, we bridge this gap by identifying *K. pneumoniae* UspG as a specific receptor for c-di-GMP. Unlike its homologs that fine-tune physiology [45], we demonstrate that UspG in *K. pneumoniae* has been coopted to control antibiotic resistance. Mechanistically, we demonstrate that UspG binds c-di-GMP via a non-canonical interface anchored by conserved residues N39 and K116. This ligand-binding event empowers UspG to function as a molecular antagonist against the transcriptional repressor RamR. By sequestering RamR, the c-di-GMP–UspG complex effectively derepresses the *ramA* locus, triggering a downstream cascade that fortifies lipid A biosynthesis and upregulates multidrug efflux pumps. This study thus delineates a conserved c-di-GMP–UspG–RamA signaling axis, providing a mechanistic blueprint for how *Enterobacteriaceae* integrate intracellular second messengers with fixed genetic resistance elements to survive antimicrobial pressure.

## Results

### A positive feedback loop between antibiotic stress and c-di-GMP drives adaptive resistance

**Fig 1:**
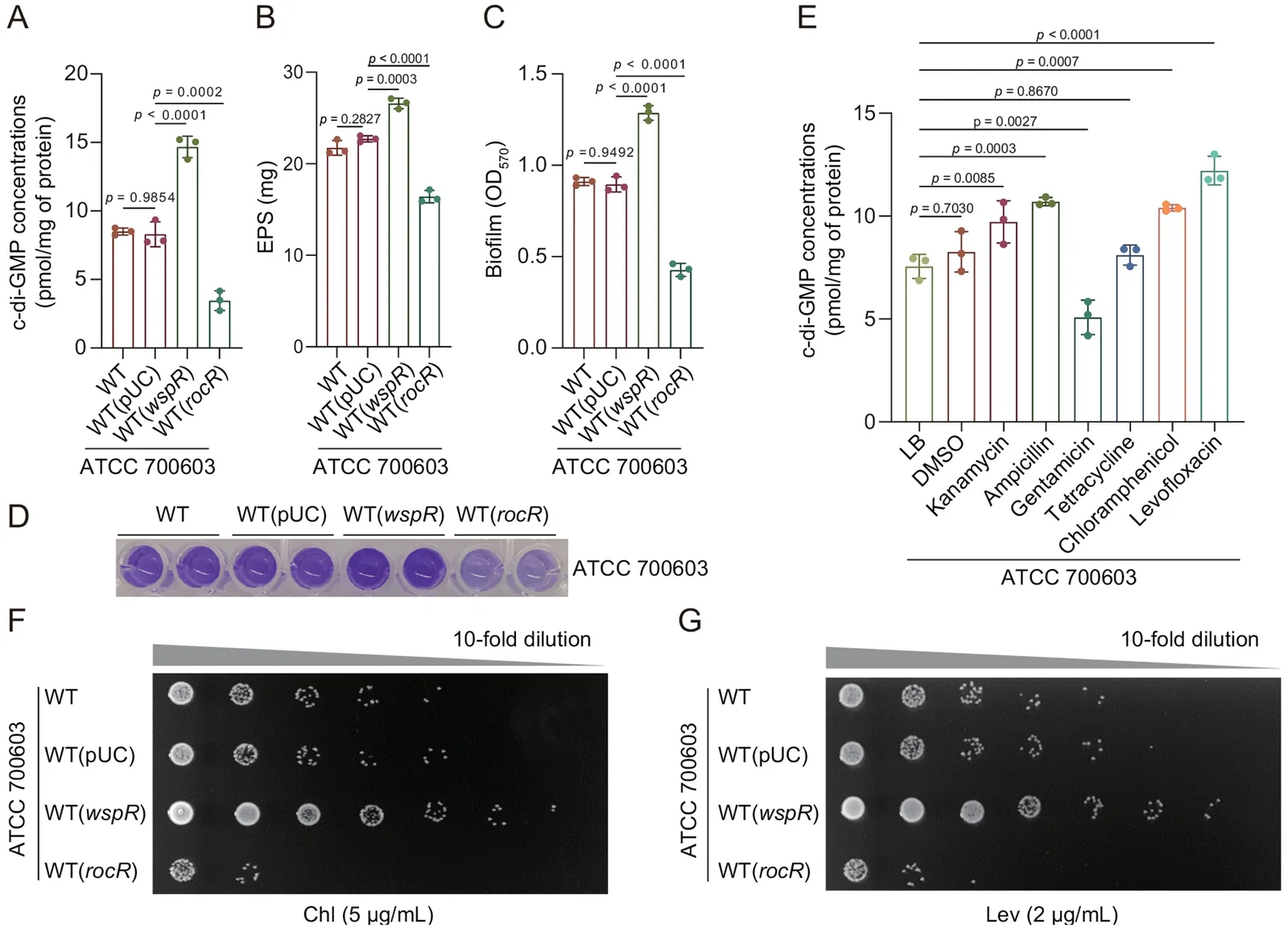
Cyclic di-GMP promotes biofilm and antibiotic tolerance phenotypes while being induced by antibiotic stress in *K. quasipneumoniae* ATCC 700603. **(A-D)** Effects of manipulating intracellular c-di-GMP levels. Wild-type (WT) *K. quasipneumoniae* ATCC 700603 transformed with an empty vector (pUC), a c-di-GMP synthase (DGC) expression plasmid (pUC-*wspR*), or a phosphodiesterase expression plasmid (pUC-*rocR*) were assayed for: **(A)** intracellular c-di-GMP concentration (quantified by LC-MS); **(B)** EPS production; **(C and D)** biofilm formation (assessed by crystal violet staining). **(E)** Antibiotic stress modulates intracellular c-di-GMP levels. ATCC 700603 WT strains were treated with sub-lethal concentrations of the indicated antibiotics (kanamycin, 20 μg/mL; ampicillin, 20 μg/mL; gentamicin, 5 μg/mL; tetracycline, 25 μg/mL; chloramphenicol, 5 μg/mL; levofloxacin, 2 μg/mL), and c-di-GMP levels were quantified by LC-MS. Antibiotic-free LB broth (LB) was utilized as the baseline control. For antibiotics requiring non-aqueous solubilization, an equivalent volume of DMSO was included as a vehicle control. **(F-G)** The previously mentioned *wspR*- and *rocR*-expressing ATCC 700603 strains were determined for susceptibility to **(F)** chloramphenicol and **(G)** levofloxacin via spot dilution assays. Data are presented as mean ± standard deviations (SD) (*n* = 3). Statistical significance was determined using one-way analysis of variance (ANOVA) with Tukey’s multiple comparisons test.

To dissect the regulatory impact of c-di-GMP on the antibiotic stress of the ESBL-producing strain of *K. quasipneumoniae* ATCC 700603, we engineered strains with modulated intracellular c-di-GMP regimes. We introduced the diguanylate cyclase (DGC) WspR [46] and the phosphodiesterase RocR [47] (derived from *Pseudomonas aeruginosa*) to elevate or deplete c-di-GMP pools in ATCC 700603, respectively. Liquid chromatography-tandem mass spectrometry (LC-MS/MS) confirmed that *wspR* overexpression increased intracellular c-di-GMP levels by 76.8%, whereas *rocR* overexpression reduced the pool by 58.4% relative to the ATCC 700603 wild-type containing empty plasmid strain (WT-pUC) (Fig 1A). This modulation profoundly altered the bacterium’s sessile lifestyle: high c-di-GMP levels triggered a hyper-biofilm phenotype, characterized by a 47.9% increase in exopolysaccharide (EPS) production and a 43.7% increase in biofilm biomass (Fig 1B and 1C). Conversely, c-di-GMP depletion via RocR significantly impaired biofilm architecture, reducing EPS and biomass by 38.9% and 52.3%, respectively, a finding further corroborated by crystal violet staining assays (Fig 1D). Additionally, acknowledging the taxonomic and clinical divergence of these pathogens, we also performed parallel investigations using the hypervirulent *K. pneumoniae* ATCC 43816. These assays yielded highly consistent results, confirming that c-di-GMP modulation exerts a conserved regulatory control over EPS synthesis and biofilm formation across both distinct genetic backgrounds of *Klebsiella* (S1A Fig).

Given the established correlation between biofilm developments and c-di-GMP concentrations, we investigated the impact of clinically relevant antibiotics, commonly used in the treatment of *Klebsiella* infections, on intracellular c-di-GMP levels. Subsequently, the ATCC 700603 wild-type strain were exposed to sub-inhibitory concentrations of clinically relevant antibiotics. Strikingly, treatment to chloramphenicol and levofloxacin elicited a robust stress response, elevating intracellular c-di-GMP levels by 48.7% and 54.0%, respectively (Fig 1E), suggesting this increase in c-di-GMP was linked to antimicrobial treatments. Plate gradient dilution assays revealed that the high-c-di-GMP strain (WT-*wspR*) exhibited significant reduction in susceptibility not only to chloramphenicol and levofloxacin (Fig 1F and 1G) but also to kanamycin and tetracycline (S1B Fig). These antibiotic susceptibility observations were corroborated by quantitative time-kill assays, results demonstrating that high cellular c-di-GMP concentration helps ATCC 700603 survive under chloramphenicol and levofloxacin treatment (S1C Fig). However, c-di-GMP depletion (WT-*rocR*) sensitized *K. quasipneumoniae* to these antibiotic treatments (Fig 1F, 1G and S1C Fig). These results establish a vicious cycle that antibiotic pressure elevates c-di-GMP, which in turn fortifies the biofilm matrix and mediates decreased antimicrobial susceptibility, promoting bacterial survival under antibiotic therapy. Similarly, these antibiotic-induced c-di-GMP fluctuations and the subsequent changes in antibiotic susceptibility phenotypes were fully recapitulated in the hypervirulent *K. pneumoniae* ATCC 43816 strain (S1D Fig and S1E Fig), underscoring that the c-di-GMP signaling network drives adaptive resistance across clinically distinct lineages.

### Identification of UspG as a c-di-GMP receptor governing antibiotic susceptibility of ATCC 700603

**Fig 2:**
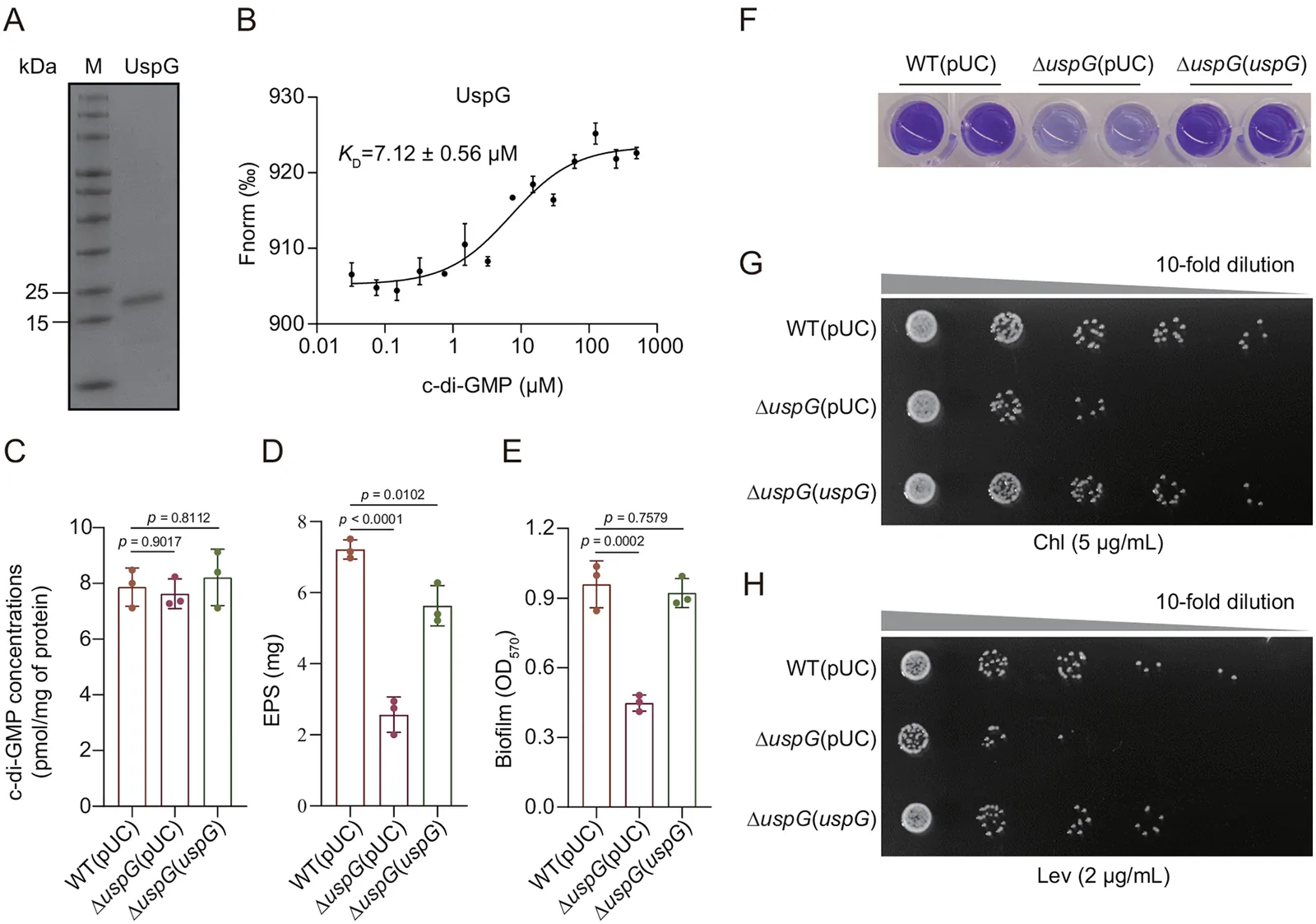
UspG functions as a c-di-GMP receptor modulating pathogenicity in *K. quasipneumoniae* ATCC 700603. **(A)** SDS-PAGE analysis of the purified 6 × His-tagged UspG protein. **(B)** Binding affinity of UspG for c-di-GMP determined by MST. The *K*_*D*_ values are presented as mean ± standard deviations (SD) of 3 biological replicates “Fnorm (‰)” indicates the fluorescence time trace changes in the MST response. **(C-H)** Physiological characterization of the *uspG* deletion mutant in ATCC 700603. ATCC 700603 WT, *uspG* deletion mutant (Δ*uspG*), and complemented strain were assayed for intracellular c-di-GMP level **(C)**, EPS production **(D)**, biofilm formation **(E and F)**, and susceptibility to chloramphenicol **(G)** and levofloxacin **(H)**. Data are presented as mean ± SD (*n* = 3). Statistical significance was determined using one-way analysis of variance (ANOVA) with Dunnett’s multiple comparisons test.

To isolate the elusive c-di-GMP effectors in ATCC 700603, we employed an unbiased affinity purification approach using biotinylated c-di-GMP as the capture probe (S2A Fig). To preclude the omission of low-abundance or weak-affinity interactors, all affinity-captured complexes were subjected to liquid chromatography‒tandem mass spectrometry (LC-MS/MS) (S1 Table). Comparative profiling against the negative control (non-biotinylated bead matrix) identified five high-confidence candidates based on spectral counts and unique peptide enrichment: BcsE (AVR78_03320), SbcD (AVR78_10055), UspG (AVR78_17055), RimO (AVR78_13060), and a GGDEF domain-containing protein (AVR78_21390) (S2B Fig). We prioritized these candidates for validation based on novelty and structural logic. We excluded BcsE, a known c-di-GMP receptor involved in cellulose biosynthesis conserved in *E. coli* [48], and the GGDEF-containing protein, which is a predicted DGC rather than a canonical receptor [49]. Then the remaining three candidates—SbcD, RimO, and the universal stress protein UspG were investigated by microscale thermophoresis (MST) assays. While SbcD and RimO displayed no detectable affinity for the ligand (S2C and S2D Fig), c-di-GMP bound to purified UspG with a dissociation constant (*K*_D_) of 7.12 ± 0.56 μM (Fig 2A and 2B), identifying UspG as a direct c-di-GMP receptor in ATCC 700603. Subsequently, by quantifying *uspG* transcript levels in *wspR*- and *rocR*-expressing strains, as well as in antibiotic-treated cells, we confirmed that the intracellular concentration fluctuations of c-di-GMP exert no obvious effects on *uspG* transcription in ATCC 700603 (S2E and S2F Fig).

Given the 100% sequence identity of the *uspG* encoding sequence between the ATCC 700603 and ATCC 43816 strains, next, we selected ATCC 700603 as the primary genetic background to explore the biological function of *uspG.* We initially constructed a deletion mutant, Δ*uspG*, using CRISPR-Cas9 mediated editing. Importantly, the deletion of *uspG* did not alter bacterial growth kinetics (S3A Fig) nor did it affect the intracellular c-di-GMP pool (Fig 2C), suggesting that UspG functions as a downstream effector rather than a c-di-GMP metabolizing enzyme. Phenotypically, compared to the wild-type strain, the Δ*uspG* mutant exhibited 40.2% and 53.3% reductions in EPS production and biofilm biomass, respectively (Fig 2D and 2E), a defect visually in accordance with crystal violet staining (Fig 2F). These results suggest that the loss of UspG severely compromised biofilm formation of ATCC 700603. Furthermore, the mutant displayed heightened susceptibility to chloramphenicol and levofloxacin (Figs 2G, 2H, and S3B Fig). All phenotypes were restored to wild-type levels in the complemented strain. Finally, to rigorously establish UspG as the primary signal transducer for c-di-GMP, we performed genetic epistasis analysis. We previously demonstrated that manipulation of elevating c-di-GMP (via *wspR* overexpression) drives a hyper-biofilm and resistant phenotype in the wild-type. However, this c-di-GMP-mediated enhancement was significantly blunted in the Δ*uspG* background (S3C and S3D Fig). Furthermore, a similar profile of heightened antimicrobial susceptibility following *uspG* deletion, and its subsequent reversal upon *uspG* complementation, was independently observed in the hypervirulent *K. pneumoniae* strain ATCC 43816 (S3E Fig). These epistatic blocking effect further demonstrates that UspG acts as the indispensable conduit linking intracellular c-di-GMP accumulation to the regulation of EPS, biofilm formation, and antibiotic susceptibility in clinically distinct lineages of *Klebsiella*.

### Residues N39 and K116 define a conserved c-di-GMP binding motif essential for UspG function

**Fig 3:**
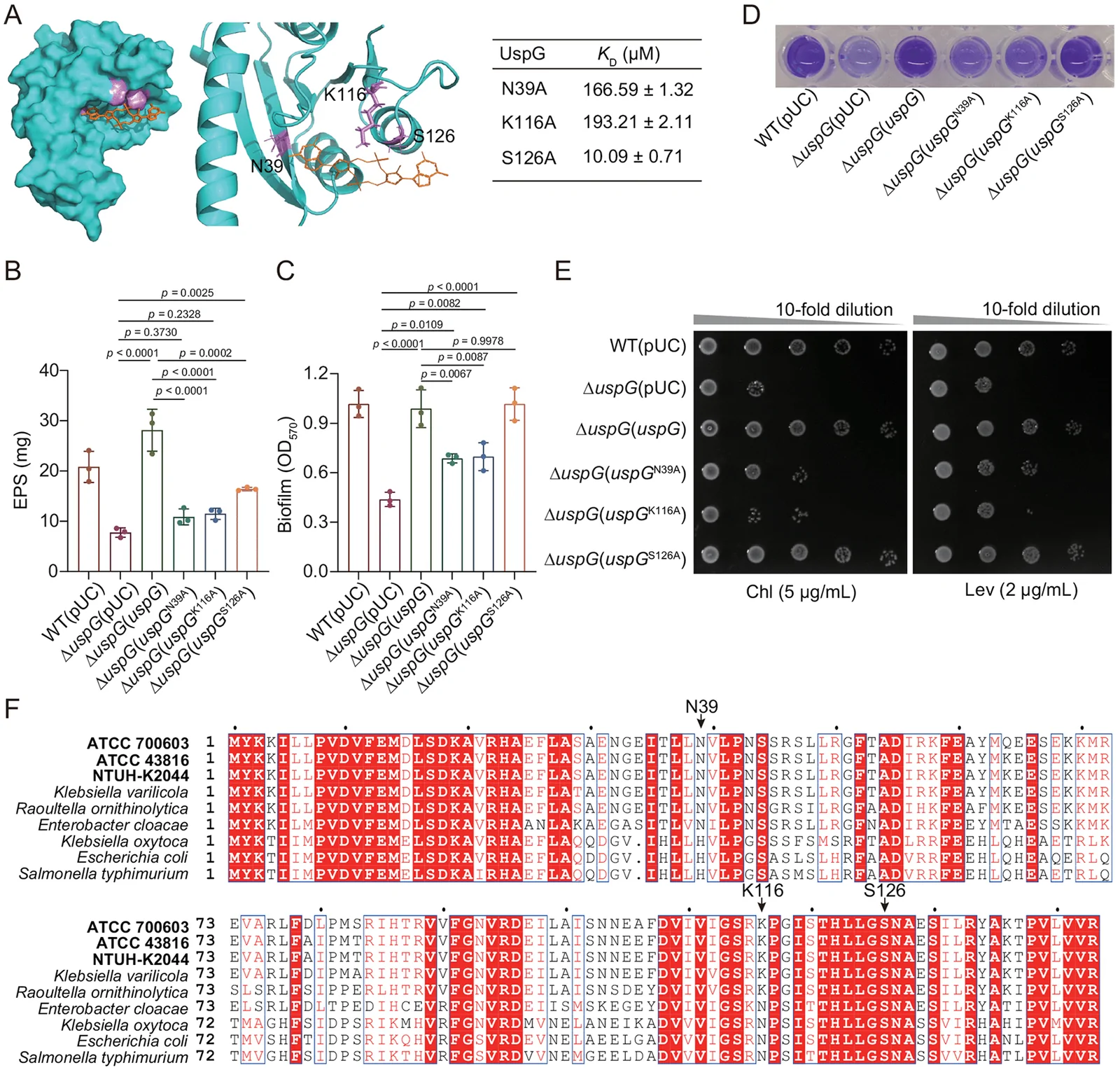
Identification of UspG conserved residues, N39 and K116, critical for c-di-GMP binding. **(A)** Predicted structural model of the UspG–c-di-GMP complex. Surface representation of the structural model of UspG in complex with c-di-GMP. c-di-GMP is shown as orange sticks. Binding affinities of UspG variants for c-di-GMP determined by MST. The dissociation constants *K*_D_ for WT and residues mutants (N39A, K116A, and S126A) are shown. **(B-E)** Phenotypic consequences of disrupting the c-di-GMP binding pocket. *K. quasipneumoniae* ATCC 700603 strains expressing WT UspG or the indicated mutants were assayed for EPS production **(B)**, biofilm formation **(C and D)**, and susceptibility to chloramphenicol and levofloxacin **(E)**. **(F)** Multiple sequence alignment of UspG orthologs from representative *Enterobacteriaceae*. Black arrows indicate the conserved residues (N39 and K116) essential for c-di-GMP binding. Species shown include *K. quasipneumoniae* strains ATCC 700603 (WP_004206026.1), ATCC 43816 (AIK79535.1), NTUH-K2044 (BAH63277.1), *K. variicola* (WP_008805007.1), *K. oxytoca* (QGN37170.1), *Raoultella ornithinolytica* (WP_133599643.1), *Enterobacter cloacae* (HGE7079822.1), *Salmonella Typhimurium* (ECS5601590.1), and *Escherichia coli* (CQR80208.1). Data are presented as mean ± SD (*n* = 3). Statistical significance was analyzed using one-way Analysis of Variance (ANOVA) with Tukey’s multiple-comparison test.

To elucidate the basis of the c-di-GMP–UspG interaction, we performed *in silico* molecular docking using the AlphaFold2-predicted structure of ATCC 700603 UspG. AutoDock (version of 1.57) analysis revealed a high-affinity binding pocket of UspG to c-di-GMP, with a calculated binding energy of -8.1 kcal/mol. Visual inspection of c-di-GMP–UspG complex suggested that the interface is stabilized by three key residues, Asn39 (N39), Lys116 (K116), and Ser126 (S126) (Fig 3A). Next, we validated this predicted interaction by generating three single-amino acids mutants of UspG (S4A Fig) and quantified their binding affinity for c-di-GMP using MST. Results revealed that substitution of N39 or K116 with alanine drastically impaired c-di-GMP binding while mutant of S126A had no impact on c-di-GMP recognition (Fig 3A). The dissociation constants *K*_*D*_ for UspG^N39A^ and UspG^K116A^ increased to 166.6 ± 1.3 µM and 193.2 ± 2.1 µM, respectively, representing a more than 20-fold reduction in affinity compared to the wild-type protein. Crucially, this loss of binding capacity translated directly to a loss of biological function. In complementation assays, expression of the c-di-GMP binding-deficient variants UspG^N39A^ or UspG^K116A^ failed to rescue the defects in EPS production (Fig 3B), biofilm formation (Fig 3C and 3D), and the reduced susceptibility observed in the Δ*uspG* mutant (Fig 3E). In contrast, the UspG^K116A^ variant fully restored wild-type phenotypes (Fig 3B**-**3E). These data confirm that residues N39 and K116 constitute the critical c-di-GMP binding motif and that direct ligand binding is indispensable for UspG-mediated regulation.

To determine the evolutionary conservation of this signaling mechanism, we found UspG homologous protein sequences from bacteria, fungi, and plants (S2 Table). Although UspG is widely conserved, we hypothesized that c-di-GMP binding capability would strictly correlate with the presence of the N39/K116 motif (Fig 3F). We purified UspG homologs from *Enterobacteriaceae* family and tested their affinity (S4B Fig). Consistent with our hypothesis, homologs retaining the conserved N39/K116 pair—such as those from *K. pneumoniae* ATCC 43816, *K. variicola*, *Raoultella ornithinolytica*, and *Enterobacter cloacae*—were capable of binding c-di-GMP (S4C- S4I Fig). Notably, the *E. cloacae* homolog, which retains N39 but lacks K116, exhibited significantly reduced affinity (*K*_D_ of 58.83 ± 1.69 μM), further underscoring the cooperative importance of both residues (S4F Fig). Conversely, homologs from *K. oxytoca*, *Salmonella typhimurium*, and *Escherichia coli*, which naturally lack these specific residues, showed no affinity for c-di-GMP (S4G – S4I Fig). Collectively, these findings further establish the N39/K116 pair as a predictive structural signature for c-di-GMP sensing within the UspG family, suggesting this regulatory pathway is functionally preserved in specific *Enterobacteriaceae* lineages.

### *ramA* is the key downstream effector of the UspG regulatory axis

**Fig 4:**
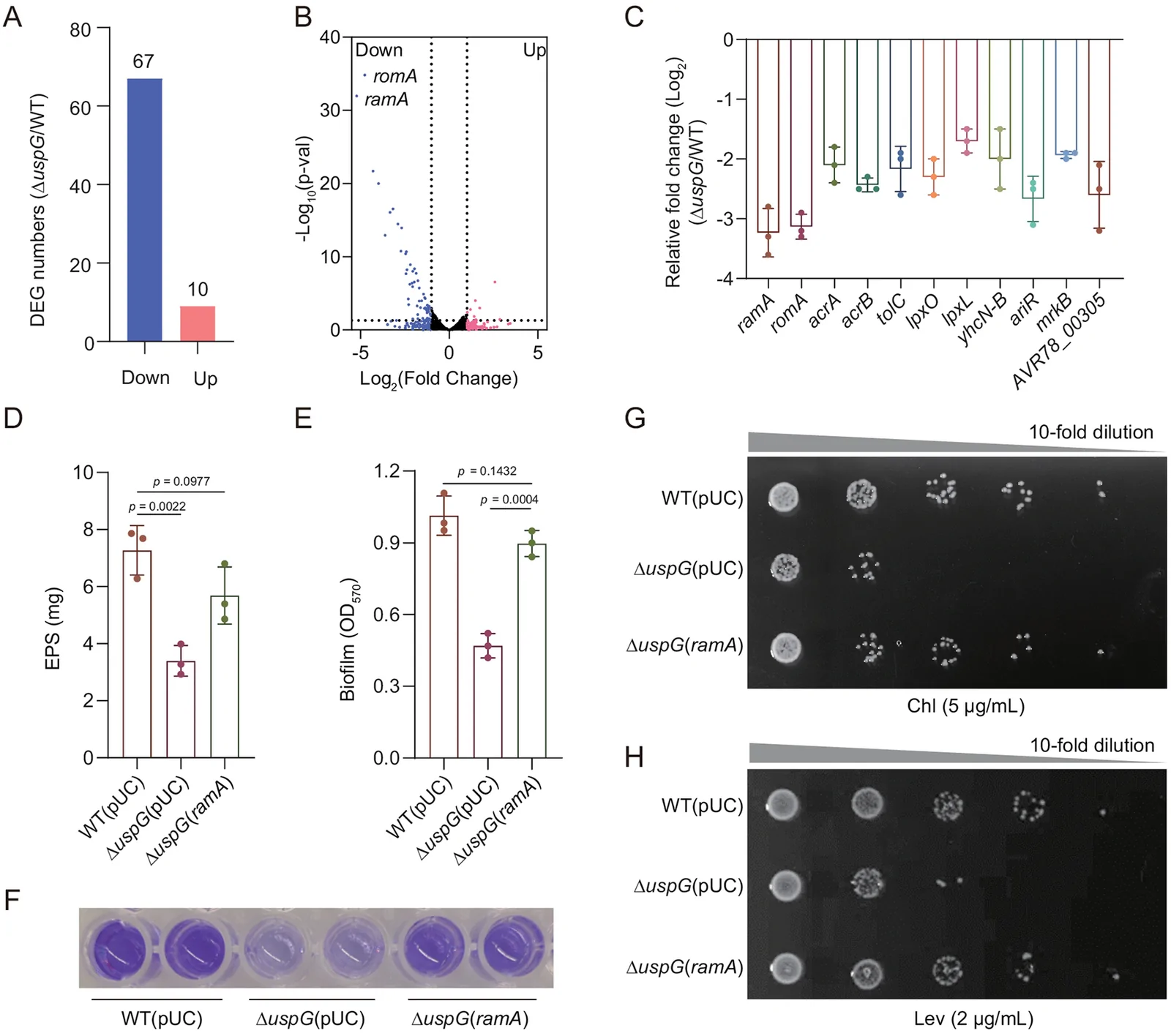
Transcriptomic profiling identifies RamA as a key downstream effector of UspG in *K. quasipneumoniae* ATCC 700603. **(A and B)** Global transcriptional changes in the ATCC 700603 Δ*uspG* mutant compared to the ATCC 700603 WT strain. **(A)** Number of differentially expressed genes (DEGs). **(B)** Volcano plot showing the distribution of DEGs; red and blue dots indicate significantly upregulated and downregulated genes, respectively (cut off by Log_2_ fold change ≥ 1, value of *p* < 0.05). **(C)** RT-qPCR analysis of selected DEGs involved in antibiotic efflux (*acrA/B*), fimbriae (*mrkB*), and regulon (*ramA*). **(D-H)** Overexpression of *ramA* rescues the phenotypic defects of the *uspG* mutant. The Δ*uspG* strain transformed with a *ramA*-expressing plasmid was assayed for EPS production **(D)**, biofilm formation **(E and F)**, and susceptibility to chloramphenicol **(G)** and levofloxacin **(H)**. Data are presented as mean ± SD (*n* = 3). Statistical significance was analyzed using one-way Analysis of Variance (ANOVA) with Tukey’s multiple-comparison test.

To further characterize the transcriptional network governed by UspG, RNA-Seq was performed in ATCC 700603 Δ*uspG* and WT strains. Transcriptomic profiling identified 77 differentially expressed genes (DEGs) (S3 Table), with 67 downregulated and 10 upregulated in the Δ*uspG* mutant (Fig 4A). Functional enrichment analysis highlighted pathways including ABC transporters, lipopolysaccharide biosynthesis, bacterial chemotaxis, and beta−lactam resistance. Notably, volcano plot analysis revealed a significant down-regulated operon of *romA*-*ramA* in the Δ*uspG* background (Fig 4B). The *romA* gene encodes a metallo-β-lactamase RomA, while RamA functions as a global AraC-type transcriptional activator known to drive multidrug resistance by upregulating the *acrAB* efflux pump operon, the small RNA gene *micF*, and the lipid A biosynthesis genes (*lpxO/lpxL*) [50–52]. Consistent with this, genes encoding the biofilm determinants (e.g., *ariR* and AVR78_*00305*) were also suppressed. Furthermore, RT-qPCR confirmed the reliability of these transcriptomic signatures (Fig 4C).

Given the profound downregulation of *ramA*, we hypothesized that it serves as the major effector of UspG. To test this, we performed phenotypic rescue experiments. In trans overexpression of *ramA* effectively restored the EPS and biofilm formation in the ATCC 700603 Δ*uspG* mutant, reaching 88.7% and 88.4% of the ATCC 700603 wild-type (WT-pUC) levels, respectively (Fig 4D and 4E). Consistently, this complementation reinstated the antibiotic tolerance of Δ*uspG* to antibiotics including chloramphenicol, levofloxacin, kanamycin and tetracycline (Figs 4G**-**4H, and S5A-S5B Fig). Together, these findings confirm that UspG exerts a functionally conserved role in driving adaptive antimicrobial recalcitrance across distinct clinical lineages. To confirm that *ramA* is essential for these phenotypes, we generated a ATCC 700603 Δ*ramA* knockout mutant. As expected, the Δ*ramA* strain phenocopied the Δ*uspG* mutant, exhibiting significant defects in EPS production (40.8% reduction) and biofilm formation (50.9% reduction) (S5C – S5E Fig), alongside heightened antibiotic susceptibility (S5F – S5I Fig), without affecting growth kinetics (S6A Fig). Furthermore, we constructed an IPTG-inducible system to verify the genetic hierarchy. In wild-type cells, induction of *uspG* expression led to a dose-dependent increase in *ramA* transcription (S6B Fig). Collectively, these genetic data establish *ramA* as the critical downstream element through which UspG orchestrates pathogenicity and drug susceptibility.

### UspG regulates *ramA* transcription by acting as a molecular antagonist of the repressor RamR

**Fig 5:**
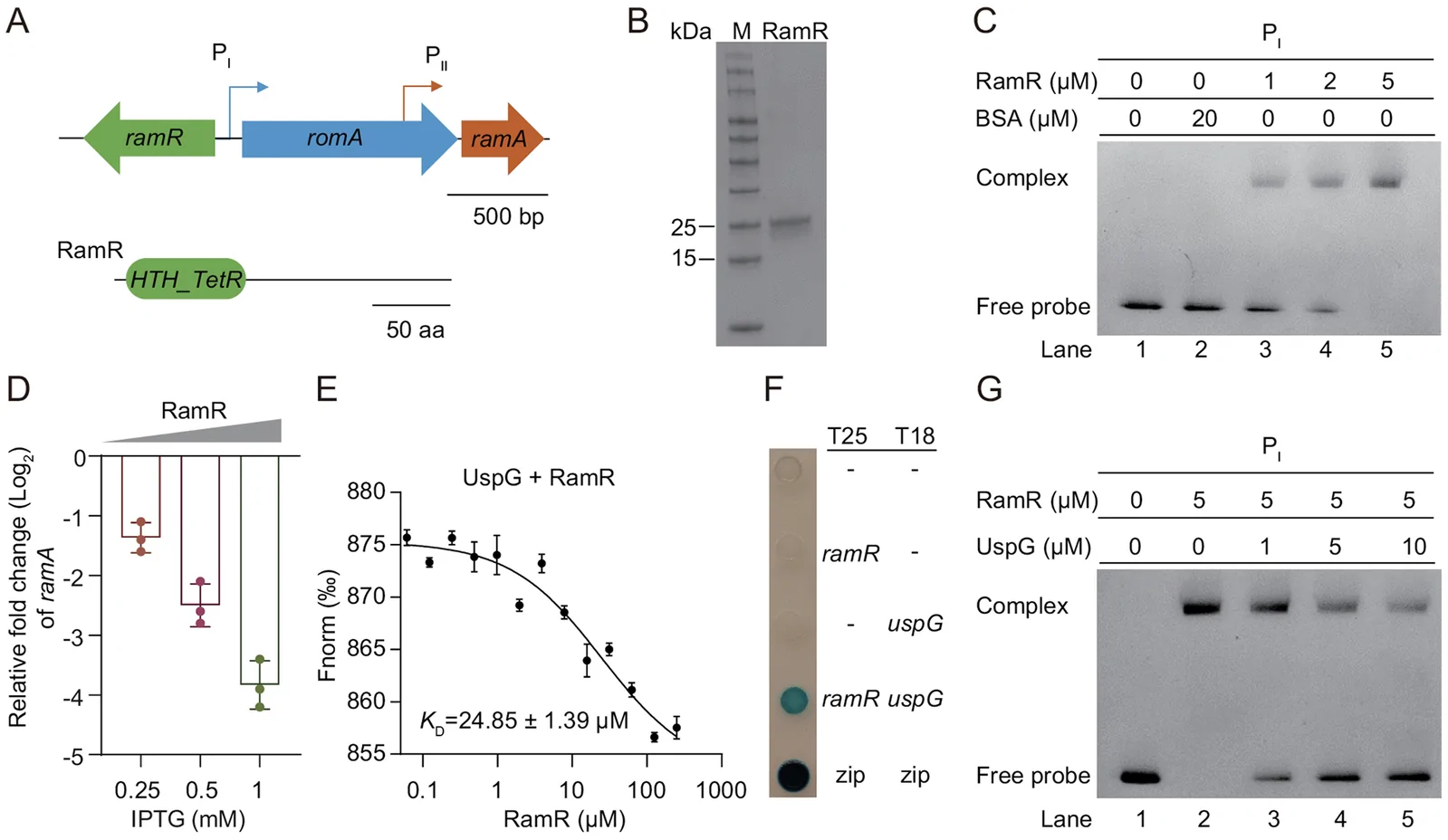
UspG antagonizes RamR repression via direct protein–protein interaction in ATCC 700603. **(A and B)** Characterization of the transcriptional repressor RamR. **(A)** Genomic organization of the *ramA/ramR* region and the predicted domain architecture of RamR. **(B)** SDS-PAGE of purified recombinant RamR. **(C)** EMSA showing dose-dependent binding of RamR to the *ramA* promoter **(P**_**I**_). **(D)** RT-qPCR analysis showing that IPTG-induced expression of RamR significantly represses *ramA* transcription in the ATCC 700603 strain. **(E)** MST analysis of the UspG bind with RamR. The *K*_*D*_ values are presented as mean ± SD of 3 biological replicates. **(F)** The interaction between UspG and RamR was detected by Bacterial Adenylate Cyclase Two-Hybrid (BACTH) assay. The interaction between UspG-T18 and RamR-T25 reconstructs adenylate cyclase activity, empty vectors and leucine-zipper fusions served as negative and positive controls, respectively. **(G)** UspG disrupts the RamR–DNA complex. Competitive EMSA showing that the addition of increasing concentrations of UspG prevents RamR from binding to the *ramA* promoter of P_I_. Data are presented as mean ± SD (*n* = 3).

We next investigated the molecular mechanism linking UspG to *ramA* activation. As previously reported, the *ramA* locus is regulated by two promoters, the repressor-controlled P_I_ promoter (co-transcribed with *romA*) and an internal P_II_ promoter [50] (Fig 5A). The TetR-family repressor RamR is known to inhibit transcription by binding to the upstream region of *romA/ramA* locus [53–55]. We first tested if UspG acts as a transcription factor. Electrophoretic mobility shift assays (EMSAs) showed that purified UspG does not bind directly to either the P_I_ or P_II_ promoters, regardless of the presence of c-di-GMP (S7 Fig). We therefore hypothesized that UspG functions indirectly by modulating RamR. First, we validated the RamR repressor function and found that purified RamR (22.3 kDa) (Fig 5B) bound specifically to the P_I_ promoter (but not P_II_) *in vitro* (Figs 5C and S8A Fig), and its overexpression *in vivo* significantly repressed *ramA* transcription (Fig 5D).

We then investigated the direct interaction between UspG and RamR. MST assays demonstrated that UspG binds to RamR with a dissociation constant *K*_*D*_ of 24.9 ± 1.4 μM *in vitro* (Fig 5E). This interaction was further corroborated *in vivo* using a bacterial two-hybrid system, where the UspG-RamR interaction reconstituted adenylate cyclase activity in *E. coli* BTH101 (Fig 5F). Crucially, we postulate whether this protein-protein interaction would interfere with RamR’s ability to bind DNA. In competitive EMSA experiments, the addition of UspG dose-dependently abolished the formation of the RamR- P_I_ promoter complex (Fig 5G). Collectively, our data demonstrated that UspG binds to RamR, disrupting RamR DNA-binding ability and thus alleviating the transcriptional repression of *ramA.*

### Cyclic di-GMP potentiates the UspG–RamR interaction to drive *ramA* expression

**Fig 6:**
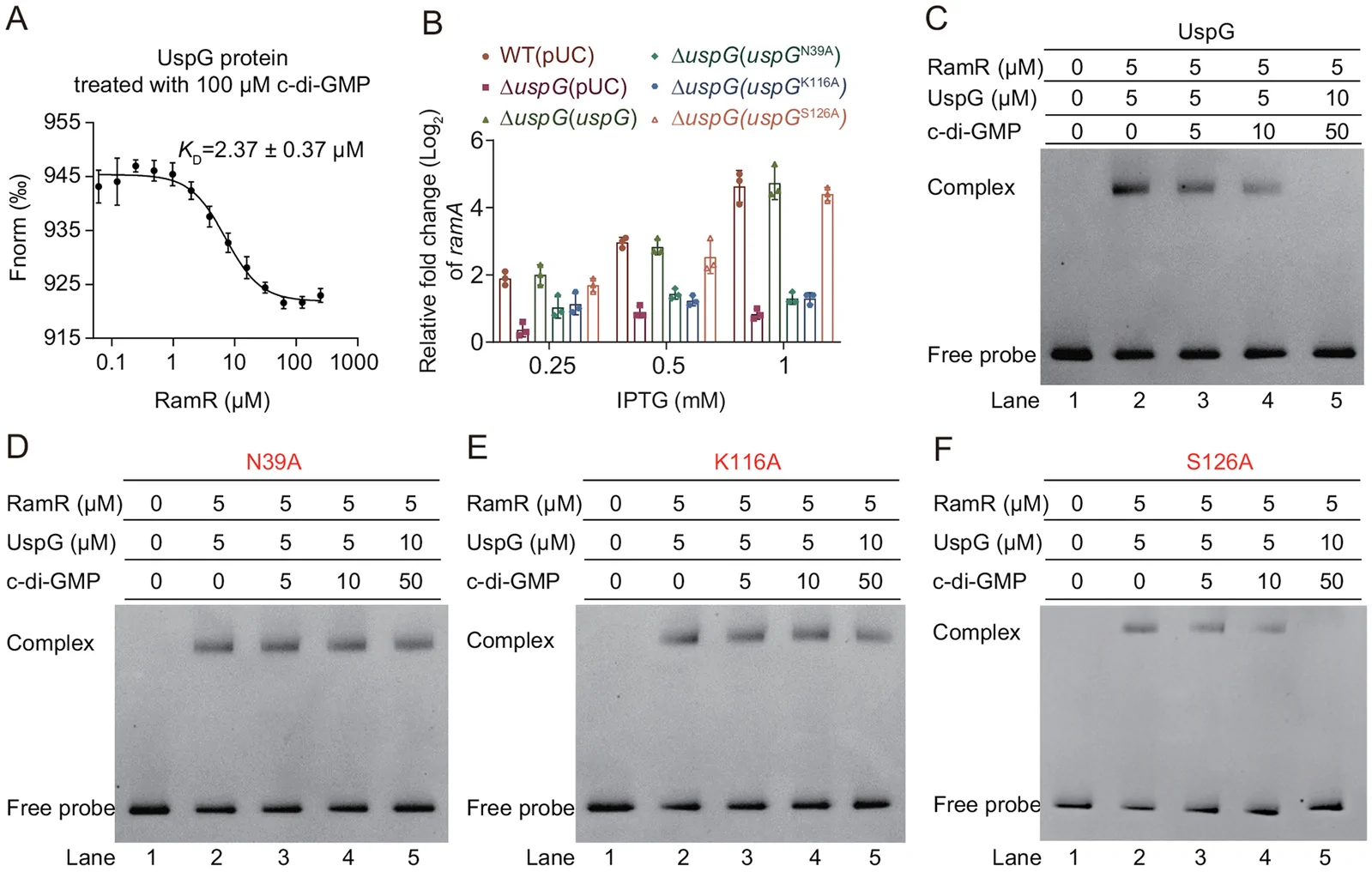
Cyclic di-GMP potentiates the interaction between UspG and RamR in ATCC 700603. **(A)** MST analysis of the UspG bind with RamR in presence of 100 uM c-di-GMP. Note the lower *K*_D_ compared to the binding of UspG to RamR in the absence of c-di-GMP (Fig 5E**). (B)** Gene *ramA* transcription is c-di-GMP dependent. RT-qPCR analysis of the transcriptional level of *ramA* in the wild type strain expressing the cyclase *wspR*. Intracellular c-di-GMP levels were titrated by inducing WspR expression with increasing concentrations of IPTG. **(C-F)** C-di-GMP-enhanced antagonism requires a functional binding pocket. EMSAs assessing the ability of WT UspG **(C)**, N39A **(D)**, K116A **(E)**, and S126A **(F)** variants to disrupt the RamR–DNA complex in the presence of increasing concentrations of c-di-GMP. Note that c-di-GMP facilitates RamR displacement by WT and S126A UspG, but fails to enhance the antagonistic effect of the binding-deficient mutants N39A and K116A. Data are presented as mean ± SD (*n* = 3).

Having established this protein-protein interaction network, we next investigated whether and how the c-di-GMP signaling ligand modulates the UspG–RamR complex. We hypothesized that c-di-GMP acts as an allosteric switch to modulate the affinity of UspG for RamR. Strikingly, MST assays revealed that the presence of c-di-GMP increased the binding affinity of UspG for RamR by approximately 10-fold (*K*_D_ improved from 24.9 μM to 2.4 μM) (Figs 6A *vs* 5E). Furthermore, to validate this ligand-dependent activation *in vivo*, we utilized the UspG mutants (N39A, K116A and S126A) characterized in our earlier structural analysis. The intracellular c-di-GMP levels were manipulated by inducing the cyclase WspR (harbored on pME-*wspR*), followed by the quantification of *ramA* transcription. In strains expressing wild-type UspG or the S126A variant, high c-di-GMP levels triggered a robust upregulation of *ramA* (Fig 6B). In contrast, this c-di-GMP-driven activation was completely abolished in the Δ*uspG* background or in strains complemented with the UspG mutants (N39A or K116A) (Fig 6B). These findings indicate that high intracellular c-di-GMP levels alone are insufficient to trigger *ramA* expression without a functional UspG receptor.

Finally, EMSA was performed to further verify this regulatory switch. Our results revealed that the RamR and P_I_ promoter complex band intensities gradually weakened due to wild-type UspG and S126A mutant with increasing concentrations of c-di-GMP (Fig 6C and 6F), but N39A and K116A did not show such effect (Fig 6D and 6E). These experimental results were consistent with the results of c-di-GMP promoting *ramA* expression *in vivo* (Fig 6B). Intriguingly, the binding strength of RamR to the P_I_ promoter was reduced by the addition of UspG variant of N39A or K116A (S8B and S8C Fig), suggesting that N39 and K116 are key resides for c-di-GMP binding, but not significantly affect the binding of UspG to RamR. In addition, c-di-GMP failed to influence the binding of RamR and P_I_ promoter complexes in the absence of UspG, suggesting that the critical role of UspG in mediating c-di-GMP-dependent regulation (S8D Fig). Collectively, our findings define a complete signal transduction pathway: c-di-GMP binds to UspG, enhancing its sequestration of the repressor RamR by an order of magnitude. This derepression trigger *ramA* transcription, thereby activating the downstream regulon governing pathogenicity and antibiotic susceptibility in ATCC 700603.

## Discussion

**Fig 7:**
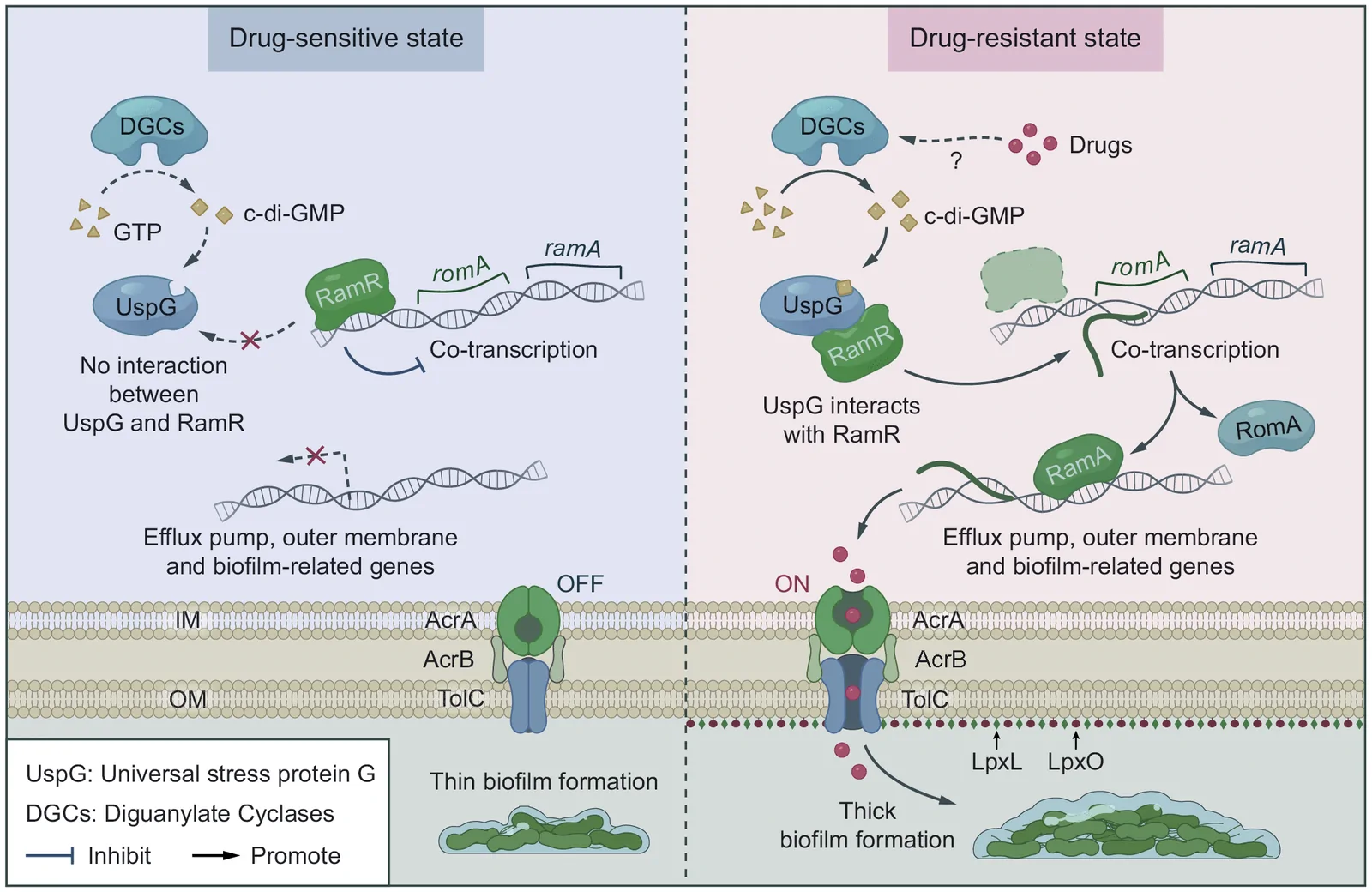
Proposed model of the c-di-GMP–UspG axis coordinating adaptive resistance and biofilm formation. Antibiotic stress triggers a surge in intracellular c-di-GMP levels in *K. pneumoniae*, driven by the activation of endogenous DGCs. This signaling molecule directly binds to the receptor UspG, strengthening its association with the TetR-family transcriptional repressor RamR. The interaction titrates RamR away from the upstream promoter region, thereby relieving transcriptional repression of the *romA*-*ramA* operon. The consequent derepression not only upregulates the global activator RamA—which unleashes a transcriptional program, activating multidrug efflux pumps, lipid A biosynthesis genes, and biofilm determinants —but also co-activates RomA, a metallo-β-lactamase that directly contributes to β-lactam resistance. By coupling sessility (biofilm) with active genetic resistance (efflux), this coordinated virulence-resistance strategy significantly enhances *K. pneumoniae* fitness and survival in the presence of antimicrobial agents.

Biofilms are universally recognized as physical fortresses that confer passive antibiotic tolerance [16]. While the second messenger c-di-GMP is the master driver of this sessile lifestyle, its role has been largely viewed as structural promoting biofilm biomass rather than actively regulating genetic resistance determinants [12,13]. Crucially, apart from the specific BrlR system in *Pseudomonas* [56,57], direct evidence linking c-di-GMP signaling to the transcriptional activation of multidrug efflux pumps—the hallmark of genetic resistance—has remained elusive. Here, we fundamentally expand this framework in *Klebsiella* by demonstrating that c-di-GMP signaling does not merely construct a physical barrier but directly reprograms the pathogen’s genetic resistance profile (Fig 7). We show that antibiotic exposure (chloramphenicol and levofloxacin) triggers an intracellular c-di-GMP surge (Fig 1E), which promotes the interaction between UspG and the TetR-family repressor RamR (a TetR-family member). This active sequestration relieves the suppression of the global regulator RamA, thereby directly activating multidrug efflux pumps and lipid A biosynthesis. By coupling c-di-GMP to resistance determinants via the UspG-RamR-RamA axis, the bacterium coordinates a dual defense: simultaneously establishing a biofilm stronghold and activating drug extrusion. Unlike passive tolerance, this response allows *K. pneumoniae* to replicate even under substantial antibiotic pressure, defining a state of active resilience.

The *ramR/ramA* locus acts as a critical master switch for multidrug resistance in *Enterobacteriaceae* [50,51,53–55,58]. Clinical studies have shown that approximately 12% of ESBL-producing clinical isolates from ICUs exhibit *ramA* overexpression due to *ramR* or *ramAR* promoter mutations, conferring cross-resistance to ciprofloxacin, tigecycline, and chloramphenicol [53]. However, the physiological signals that antagonize the TetR-family repressor RamR during infection have remained obscure. Our study elucidates this regulatory mechanism by identifying UspG as a dynamic, signal-dependent antagonist of RamR, demonstrating that *uspG* deletion represses *ramA* and *marB* expression. Furthermore, we observed that *ramA* ablation in *K.* *quasipneumoniae* ATCC 700603 resulted a 50.9% collapse in biofilm biomass and a 40.8% reduction in EPS production (S5C-S5E Fig). This positive regulatory dynamic diverges from the previously discovered mechanisms in *E. coli* and *S. enterica*, wherein the overexpression of MarA or RamA represses biofilm development [59,60]. This phenotypic paradox reflects a species-specific evolutionary rewiring of stress responses. Unlike curli-dependent biofilms in *E. coli* or *Salmonella*, *Klebsiella* relies on robust capsules and EPS. Lacking motility for dispersal, *Klebsiella* co-opts the RamA regulon alongside the c-di-GMP effector UspG. Consequently, RamA activation concurrently drives *acrAB*-mediated multidrug efflux and EPS fortification, orchestrating a dual defense strategy that maximizes antimicrobial resilience.

Although USPs are ancient architects of bacterial survival [38,40], their classically defined roles are relegated to regulate essential physiological processes, such as DNA repair [61,62], metabolic enzymes [63], osmotic balance [64], and cell wall integrity [65], through the direct sensing of c-di-AMP [43] or cAMP [44]. While previous studies have noted that c-di-GMP can influence USP transcription [66], the structural and functional integration into c-di-GMP signaling networks has remained undefined. Here, our characterization of UspG reveals an evolutionary strategy wherein *Klebsiella* co-opts this conserved structural fold to function as a specific c-di-GMP receptor. Distinct from the classic ligand-binding TetR-family repressors, e.g., EthR in *Mycobacterium* [67], UspG operates as a signal-dependent protein antagonist. This binding event acts as a molecular switch, dramatically enhancing UspG’s affinity for RamR and sequestering it from the *ramA* promoter. Consequently, this c-di-GMP–UspG–RamR axis enables the pathogen to bypass the need for slow genetic mutations and instead achieve immediate, signal-driven upregulation of the resistance machinery. Ultimately, our findings not only complete the cyclic nucleotide-sensing spectrum of the USP superfamily but also highlights a profound evolutionary plasticity, demonstrating how a conserved homeostatic domain has been rewired to actively govern acute antimicrobial recalcitrance.

Bacterial biofilms exhibit a structural and metabolic complexity convergence with eukaryotic solid tumors [68], defined by microenvironmental heterogeneity [69], adaptive metabolic reprogramming, and the maintenance of recalcitrant seed cells that drive relapse [7]. In eukaryotes, 2’3’-cGAMP represents the most extensively characterized molecule in eukaryotes [17], while c-di-GMP stands as the most profoundly studied in the prokaryotic kingdom [13]. A striking evolutionary parallel exists between the eukaryotic secondary messenger 2’3’-cGAMP and bacterial c-di-GMP: just as cGAMP triggers a systemic interferon storm to orchestrate host defense [70,71], bacterial c-di-GMP drives the synchronization of thousands of individual cells into recalcitrant biofilms [12,14]. However, a fundamental divergence exists in how these chemical motifs are interpreted by their respective hosts. While eukaryotic 2’3’-cGAMP signaling is largely centralized through the canonical STING adaptor, a monolithic pathway that funnels diverse innate immune stimuli into a unified interferon response [72,73], bacterial c-di-GMP signaling is characterized by a strikingly decentralized and pluralistic architecture [74,75]. Given that mammalian systems lack endogenous c-di-GMP signaling cascades and harbor no orthologs of the bacterial UspG receptor, the c-di-GMP–UspG–RamR axis emerges as a highly selective therapeutic target, conceptually devoid of mechanism-based host toxicity.

Finally, the coevolution of hypervirulence and antibiotic resistance in *Klebsiella* demands therapeutic strategies that go beyond simple bactericidal activity [76,77]. Our study offers a molecular framework that may aid in addressing current therapeutic challenges by defining the c-di-GMP–UspG–RamA axis as a critical hub for bacterial stress adaptation. We demonstrate that UspG functions as a regulatory nexus, connecting biofilm formation, membrane integrity (via the *mla* operon) [78], type 3 fimbriae expression (*mrkB*) [79], and drug efflux. A notable feature of this regulatory axis is the apparent absence of identifiable UspG homologs in higher metazoans, including humans. Consequently, targeting the UspG-RamR interface the offers a specific therapeutic strategy. Disrupting this interaction would achieve a two-pronged effect: simultaneously stripping the bacterium of its physical biofilm armor and neutralizing its active antibiotic susceptibility. These findings not only decode fundamental principles of bacterial signal integration but also pinpoint druggable targets for next-generation antimicrobial development. Ultimately, these insights provide a mechanistic basis for host-safe interventions that convert bacterial resilience into a therapeutic vulnerability.

## Materials and methods

**Bacterial strains and culture conditions.** Bacterial strains used in this study are cataloged in S4 Table. All plasmids and strains used in this study were verified by sequencing. *K. quasipneumoniae, K. pneumoniae,* and *Escherichia coli* strains were cultured in Luria-Bertani (LB) medium consist of 10 g/L NaCl, 10 g/L tryptone, 5 g/L yeast extract; or on LB agar (LB medium containing 15 g/L agar) at 37°C. Antibiotics were supplemented as required: streptomycin (50 µg/mL), apramycin (50 µg/mL), chloramphenicol (25 µg/mL), levofloxacin (2 µg/mL), kanamycin (50 µg/mL) or ampicillin (100 µg/mL). Isopropyl-β-D-thiogalactopyranoside (IPTG) and bromo-indolyl-galactopyranoside (X-gal) were commercially sourced from Solarbio.

**Construction of in-frame deletion mutant and complement strains.** The gene knockout of *K. quasipneumoniae,* and *K. pneumoniae* was performed using the CRISPR/Cas9 editing system [80], and the S5 Table provides a summary of the sequences of primers used. Briefly, designed sgRNAs were cloned into the pSGKP plasmid, and dsDNA homologous arms of genes of interest were amplified by fusion PCR using PrimeSTAR DNA polymerase (TAKARA). Plasmid pSGKP-sgRNA and its matched dsDNA homologous arms were electro-transformed into pCasKP-harboring *K. pneumoniae* competent cells cultured in LB supplemented with 0.2% L-arabinose. Transformants were selected with apramycin and streptomycin at 30 °C and verified by DNA sequencing. Finally, the plasmids were cured with 5% sucrose at 37 °C. In addition, the target gene was integrated into the plasmid to obtain the complemented strains by using pUC-smR, which was modified by pUC19 with ampicillin resistance gene replaced by streptomycin resistance gene. The resulting constructs were introduced into *K. pneumoniae* wild type strains or mutants using electroporation. The pME2-MCS plasmid was constructed using the following procedure. The pME2 plasmid was digested with HindIII and XhoI and the dissected plasmid was collected. A fraction containing the *tac* promoter, multiple cloning site and lactose operon repressor *lacI* was synthesized by Azenta Life Sciences. The apramycin resistance gene was amplified from pCasKP-Apr plasmid. The aforementioned fragments were connected using In-Fusion cloning, yielding the final plasmid pME2-MCS containing both apramycin resistance and an IPTG-inducible *tac* promoter. The target genes were integrated into the pME2-MCS by using In-Fusion cloning, and all plasmids were verified by DNA sequencing.

**Biofilm formation assays.** The *Klebsiella* biofilm formation assays were prepared in 96-well plates and measured using crystal violet staining as previously described [79]. Briefly, overnight cultures were diluted in LB broth until the suspension had an optical density OD_600_ of 0.05. The plates containing 150 μL of culture were then incubated for 24 hours at 37 °C without shaking. After removing the medium, PBS was used to gently clean the wells three times. The air-dried samples were fixed with methanol and then stained with 100 μL of a 0.5% crystal violet solution for 15 minutes at room temperature. The wells were cleansed three times with distilled water to remove crystal violet. Finally, 150 μL of 95% ethanol was added to the wells to dissolve the samples. The biofilm formation was measured by reading the microplates at 570 nm.

**EPS content detection.** To accurately quantify EPS production, a standardized uronic acid quantification assay were performed [81]. The strains were cultured in LB and inoculated at 37 °C with shaking until the OD_600_ of cultures were 3.0. To extract total EPS, 250 µL of the bacterial culture was mixed with 50 µL of 1% Zwittergent 3–14 in 100 mM citric acid and incubated at 50 °C for 20 min. The mixture was centrifuged at 17,000 × g for 5 min, and 100 µL of the resulting supernatant was precipitated with 400 µL of ice-cold absolute ethanol on ice for at least 20 min. The EPS precipitates were harvested by centrifugation (17,000 × g at 4 °C for 5 min), allowed to air dry at room temperature, and subsequently rehydrated in 200 µL of ultrapure water at 37 °C for 30 min. Subsequently, EPS was quantified accordingly and the final calculated EPS concentrations were normalized to the corresponding initial culture density (OD_600_). The experiment was conducted three times and the mass of the precipitate was measured by analytical balance.

**Resistant spot dilution assays**. To assess bacterial sensitivity to drugs, an agar screening spot assay was utilized as reported previously [82]. Briefly, Overnight cultures of the *K. pneumoniae* strains were uniformly adjusted to an initial density at OD_600_ of 0.5, and serial dilution was performed using fresh LB medium. For each dilution, 1 μL aliquots of each suspension were plated onto LB agar plates containing the corresponding antibiotics. The concentration of drugs used for *K. pneumoniae* and its mutants was as follows: tetracycline (25 μg/mL), chloramphenicol (5 μg/mL), levofloxacin (2 μg/mL), kanamycin (20 μg/mL) and gentamicin (5 μg/mL). The assays were repeated with three independent experiments.

**Time-kill kinetics assays.** To quantify the bactericidal dynamics, time-kill kinetics assays were subsequently performed. Bacteria were diluted in fresh LB to an initial inoculum of OD_600_ ~ 0.1, followed by the addition of the respective antibiotics at specified concentrations. The cultures were incubated at 37 °C with shaking at 200 rpm. At predetermined time intervals (0, 0.5, 1, 2, 3, and 4 h), cells were serially diluted tenfold in 0.85% NaCl, and plated onto LB agar plates. Viable cell counts were enumerated after 18 h of incubation at 37 °C. The time-kill dynamics were plotted as log_10_ CFU/mL over time. All susceptibility assays were independently conducted in biological triplicates.

**Growth rate analysis**. Growth rates of the *K. pneumoniae* wild type and mutants were determined in LB broth. In brief, the 150 μL of *K. pneumoniae* suspensions (OD_600_ ~ 0.05) was subjected in the cells to and incubated at 37 °C with shaking at 220 rpm, and the OD_600_ value was measured for determining the growth of strains, which was read by using a Synergy HTX multimode microplate reader (BioTek Instruments, Winooski, VT, USA).

**RNA-Seq and RT-qPCR.** RNA-seq of the *K. pneumoniae* wild-type and the *uspG* mutant was contracted to Shanghai Personal Biotechnology Cp., Ltd. The RNA-seq samples were harvested from mid-logarithmic phase cultures (OD_600_ ~ 1.0) grown in LB broth at 37°C with continuous shaking (200 rpm). The raw transcriptomic sequencing data have been deposited in the NCBI Sequence Read Archive (SRA) under BioProject accession number PRJNA1391018. Total RNA extraction employed TRIzol Reagent (Invitrogen), with sample quality assessed via NanoDrop spectrophotometry (Thermo Scientific) and Bioanalyzer 2100 analysis (Agilent). Using NCBI RefSeq assembly GCF_001596075.2 as the reference genome, significantly differentially expressed genes were identified under thresholds of |log_2_FoldChange| ≥ 1 and *p* value of <0.05. RNA-seq results were verified through RT-qPCR (StepOne System, ABI) detecting DEG expression levels with the 16S RNA gene of *K. pneumoniae* serving as endogenous control, HiScript III RT SuperMix (Vazyme Biotech Co., Ltd.) was used to complete the reverse transcription reaction. All RT-qPCR primer sequences were listed in S5 Table.

**Protein expression and purification.** The steps for protein expression and purification as previously described [83]. In brief, cloning of target genes into pCold I or pET30a were followed by transformation into *E. coli* BL21(DE3) competent cells. Protein expression involved culturing transformants in kanamycin-supplemented LB medium (100 μg/mL) at 37 °C with 220 rpm shaking until the OD_600_ reached 0.4-0.6. Overnight induction at 16 °C using 1 mM IPTG initiated recombinant protein production. Cells were subsequently collected by centrifugation (4000 × g, 20 min), resuspended in PBS (pH 7.4), and lysed via sonication. After clarifying the lysate through centrifugation (10000 × g, 15 min, 4 °C), the supernatant underwent purification on a 1 mL His-Tag affinity column pre-equilibrated with PBS containing 250 mM NaCl. Elution employed a linear imidazole gradient (10–300 mM) in PBS. Purified proteins were resolved on SurePAGE gels (GenScript) with purity assessed by SDS-PAGE analysis.

**Autodocking.** The molecular docking steps according to previously described [84]. Briefly, the UspG structure was retrieved from UniProt following AlphaFold prediction. Molecular docking employed AutoDock 4.2.1 with supporting AutoDock Tools and MGL Tools. The docking parameters of c-di-GMP and UspG were based on program defaults, the PyMOL was used for visualizing the resulting complex conformations, which were formed by AutoDock and ranked by energy.

**Quantitative analysis of c-di-GMP levels.** The detailed steps for extracting c-di-GMP were carried out in accordance with previously reported methods [36]. Briefly, overnight cultures were diluted in LB broth to the OD_600_ was 0.05 and incubated at 37 °C with 220 rpm shaking until the OD_600_ was 2.0. Cells were pelleted by centrifugation (4000 rpm, 2 min, 4°C), washed twice with precooled PBS, and supernatants discarded after each wash. After resuspension in precooled PBS, boiling water incubation proceeded for 10 min. The mixture was adjusted to 65% precooled ethanol and vortexed for 15 s, then the mixture was centrifuged at 4 °C and 16000 rpm for 2 min. The collected supernatant underwent rotary evaporation before dissolving in water. Finally, the LC-MS/MS was used for identification and relative quantification of c-di-GMP after the supernatant was moved to a mass spectrometry sample tube for analysis. The samples were separated on a C18 column using a binary pump system with solvent A and eluent B, the components of which were water containing 0.1% (v/v) formic acid and methanol containing 0.1% (v/v) formic acid, respectively. The gradient was maintained for 10 minutes at a flow rate of 0.3 mL min^−1^ after beginning at 10% eluent B. The analyte detection was carried out on a hybrid LCMS-IT-TOF liquid chromatograph mass spectrometer from Shimadzu (Kyoto, Japan).

**Microscale thermophoresis assay.** The MST assay was conducted on the Nano Temper Monolith NT.115 instrument (NanoTemper Technologies; www.nanotemper-technologies.com). In this study, the purified UspG and mutated proteins were labeled with Monolith His-tag Labeling Kit RED-tris-NTA second Generation (Nano Temper, Germany) followed by the manufacturer’s protocol. Briefly, the labeled protein and ligand were mixed and incubated for 10 min under room temperature. Subsequently, 10 μl of the mixtures were put into standard treated silicon capillaries (K022 Monolith NT.115, Nano Temper, Munich, Germany) and the fluorescence was measured at 60% LED power and 40% MST power. The assays were repeated with three independent experiments.

**Bacterial Two-Hybrid Assay.** The bacterial two-hybrid method based on adenylate cyclase was applied as previously described [85]. In brief, the UspG, RamR proteins were fused to the isolated catalytic domains of Bordetella adenylate cyclase. Co-transformation of both fusion plasmid constructs into reporter strain BTH101 preceded 24 h incubation at 30 °C. Triplicate colonies from each transformation were inoculated into antibiotic-supplemented LB media containing 0.5 mM IPTG. Following overnight growth at 30 °C, 1 μL aliquots were spotted onto LB agar plates added with 0.5 mM IPTG, 40 μg/mL X-gal (bromo-indolyl-galactopyranoside), and corresponding antibiotics, then incubated at 30 °C for 24 h. All assays were performed in triplicate and representative images were displayed.

**Electrophoretic mobility shift assay.** Electrophoretic mobility shift assays (EMSA) were conducted using Thermo Fisher Scientific kits with minor modifications. Briefly, PCR-purified promoters with 3’-end biotin labeling via the Biotin 3’ End DNA Labeling Kit. DNA-protein binding reactions proceeded per manufacturer’s protocols (Thermo Fisher, Waltham, USA), with interactions detected by using the LightShift Chemiluminescent EMSA Kit. DNA-protein complexes were resolved on 5% polyacrylamide gels. After UV cross-linking, membrane-immobilized biotinylated probes were visualized using Thermo Fisher’s biotin luminescence detection kit.

**Affinity pull-down assay.** Screening of c-di-GMP effector protein in *K. pneumoniae* was performed using affinity pull-down method as reported previously [86]. In brief, *K. pneumoniae* cultures in 100 mL LB were grown to OD_600_ of 0.5 at 37 °C prior to centrifugation. Cell pellets were resuspended in 1 mL reaction buffer [10 mM Tris-HCl (pH 7.5), 50 mM KCl, 1 × protease inhibitor, 1 mM DTT, 1% (v/v) n-dodecyl-β-D-maltoside, 0.5% (v/v) Triton X-100] and lysed ultrasonically. Following centrifugation, the supernatant was retained. To 50 μg soluble protein, 5 μM biotinylated c-di-GMP (B098, BioLog, USA) was added, with overnight incubation at 4 °C. Streptavidin Dynabeads (Thermo Fisher) captured biotin-c-di-GMP complexes during 30-min incubation. After three successive washes with reaction buffer, the beads were resuspended in loading buffer and boiled for 10 min. Finally, the supernatant was analyzed by using SDS-PAGE. The gel was sent to wininnovatebio Biotechnology Co., Ltd for analysis and identification. The search results employed Percolator rescoring (v2.04) to enhance matching accuracy. Spectra were filtered at 1% FDR (PSM-level FDR ≤ 0.01) to retain significant spectral matches and peptide identifications. Protein assignment utilized Mascot 2.3.02 against the UniProt database, which was identified by more than 3 unique peptides.

**Statistical analysis**. Statistical analyses were performed using Prism 8 software (GraphPad). Biological replicates and numbers of independent experiments are provided in the legends. Data are presented as mean ± standard deviations (SD). Statistical significance was determined using one-way analysis of variance (ANOVA) or Student’s *t*-test. All of the experiments presented as representative figures were repeated at least three times with similar results.

## Supporting information

S1 Fig Modulation of intracellular c-di-GMP levels governs biofilm formation and adaptive antimicrobial tolerance across distinct *Klebsiella* pathotypes. **(A)** Effects of genetically altering c-di-GMP levels in the hypervirulent *K. pneumoniae* strain ATCC 43816. Cells harboring an empty vector (pUC), a DGC expression plasmid (pUC-*wspR*), or a phosphodiesterase expression plasmid (pUC-*rocR*) were assessed for intracellular c-di-GMP concentrations, EPS, and biofilm biomass, mirroring the experimental setup in **Fig 1A–1C. (B)** *K. quasipneumoniae* wild-type ATCC 700603 strains expressing the c-di-GMP synthase WspR or the phosphodiesterase RocR were assayed for susceptibility to kanamycin and tetracycline. The experiments were repeated at least 3 times with similar results and representative figures are shown. **(C-D)** Time-kill kinetics evaluating c-di-GMP-mediated adaptive antibiotic tolerance. Survival dynamics of ATCC 700603 **(C)** and ATCC 43816 **(D)** during exposure to chloramphenicol and levofloxacin were quantified over time. **(E)** Sub-lethal antibiotic stress dynamically modulates intracellular c-di-GMP pools in ATCC 43816. Cells were exposed to the indicated antibiotics at the exact sub-lethal concentrations detailed in **Fig 1E**, with the exception of levofloxacin (0.1 μg/mL). Data are presented as mean ± standard deviations (SD) (*n* = 3). Statistical significance was determined using one-way analysis of variance (ANOVA) with Tukey’s multiple comparisons test.
(TIF)

S2 Fig Affinity capture and identification of putative c-di-GMP receptors. **(A)** SDS-PAGE analysis of pull-down eluents. Streptavidin magnetic beads were incubated with *K. quasipneumoniae* ATCC 700603 cell lysates in the presence of buffer (lane 1, negative control) or biotinylated c-di-GMP (Lane 2). M, protein molecular weight marker. **(B)** The top five candidate receptors identified by mass spectrometry (LC-MS/MS) from the specific elution fraction. **(C and D)** Validation of candidates by MST assay. Bind affinity of c-di-GMP for recombinant RimO **(C)** and ScbD **(D)** was assessed by MST. Data are presented as mean ± SD (*n* = 3). **(E-F)** Relative *uspG* transcript levels were quantified in **(E)** *K. quasipneumoniae* ATCC 700603 ectopically expressing the diguanylate cyclase WspR (pUC-*wspR*) or the phosphodiesterase RocR (pUC-*rocR*), normalized to the empty vector (pUC) control; and **(F)** ATCC 700603 cells following sub-lethal antibiotic exposure (concentrations as detailed in **Fig 1E**), compared to the LB broth baseline control.
(TIF)

S3 Fig UspG is essential for c-di-GMP-mediated phenotypic regulation. **(A)** Growth kinetics of *K. quasipneumoniae* ATCC 700603 WT and Δ*uspG* strains in LB medium. **(B)** Time-kill assay evaluating the viability of the ATCC 700603 Δ*uspG* mutant and its complemented strain upon exposure to the chloramphenicol and levofloxacin. **(C and D)** c-di-GMP fails to stimulate pathogenic phenotypes in the absence of UspG. The effect of elevating intracellular c-di-GMP (via *wspR* overexpression) on EPS production **(B)** and biofilm formation **(C)** was compared between WT and Δ*uspG* backgrounds. The Y-axis represents the relative fold change induced by *wspR* overexpression compared to the vector control (pUC). Note that the c-di-GMP-driven upregulation observed in the WT is abolished in the mutant. **(E)** Time-kill assay of the hypervirulent *K. pneumoniae* ATCC 43816 Δ*uspG* and its complemented strain against chloramphenicol and levofloxacin. Data are presented as mean ± SD (*n* = 3). Statistical significance was determined using Student’s *t*-test.
(TIF)

S4 Fig Conservation of c-di-GMP binding among UspG orthologs. **(A and B)** SDS-PAGE analysis of purified recombinant proteins. **(A)** UspG variants (N39A, K116A, S126A) of *K. quasipneumoniae* ATCC 700603 used in this study. **(B)** UspG orthologs from representative *Enterobacteriaceae* species. (C–I) MST binding curves showing the interaction between c-di-GMP and UspG orthologs from **(C)** *K. pneumoniae* ATCC 43816; **(D)** *K. variicola*; **(E)** *Raoultella ornithinolytica*; **(F)** *Enterobacter cloacae*; **(G)** *K. oxytoca*; **(H)** *Salmonella enterica* Serovar Typhimurium; and **(I)** *E. coli*. Data are presented as mean ± SD (*n* = 3).
(TIF)

S5 Fig RamA positively regulates multidrug tolerance downstream of UspG. **(A and B)** Overexpression of *ramA* restores antibiotic resistance in the ATCC 700603 Δ*uspG* background. The *uspG* mutant transformed with a *ramA*-expressing plasmid was assayed for susceptibility to **(A)** kanamycin and **(B)** tetracycline. **(C-I)** Phenotypic characterization of the *ramA* deletion mutant on the EPS production **(C)**, biofilm formation **(D and E)**, and susceptibility to chloramphenicol **(F)**, levofloxacin **(G)**, kanamycin **(H)**, and tetracycline **(I)**. Data are presented as mean ± SD (*n* = 3). Statistical significance was analyzed using one-way Analysis of Variance (ANOVA) with Tukey’s multiple-comparison test.
(TIF)

S6 Fig Transcriptional expression of *ramA* is activated by *uspG.* **(A)** Growth kinetics of *K. quasipneumoniae* ATCC 700603 WT and Δ*ramA* strains in LB medium. **(B)** Positive correlation between UspG levels and *ramA* transcription. RT-qPCR analysis of *ramA* mRNA levels *ramA* mRNA levels in *K. quasipneumoniae* cells expressing UspG under the control of an IPTG-inducible promoter. Increasing IPTG concentrations correspond to elevated intracellular UspG levels. Data are presented as mean ± SD (*n* = 3).
(TIF)

S7 Fig UspG does not directly bind to the *ramA* promoters. **(A and B)** EMSA analysis assessing the direct interaction between purified UspG and the *ramA* promoter fragments P_I_ **(A)** and P_II_ **(B)**. Assays were performed in the presence or absence of c-di-GMP. The absence of shifted bands confirms that UspG does not possess intrinsic DNA-binding activity, supporting the model that it functions *via* protein–protein interaction with RamR rather than direct transcriptional regulation. Data are representative of three independent experiments.
(TIF)

S8 Fig Specificity of RamR binding and control experiments. (**A)** RamR specifically binds to the *ramA* P_I_ promoter. EMSA analysis of RamR incubation with the *ramA* P_II_ promoter fragment. A reaction containing RamR and the pI promoter served as a positive control to define the migration of the RamR–DNA complex. **(B and C)** Interaction of UspG mutants with RamR. Competitive EMSA assessing the ability of the UspG variants **(B)** N39A and **(C)** K116A to dissociate RamR from the P_I_ promoter in the absence of c-di-GMP. **(D)** c-di-GMP does not directly disrupt the RamR–DNA complex. EMSA analysis of pre-formed RamR–P_I_ complexes treated with increasing concentrations of c-di-GMP in the absence of UspG. The persistence of the shifted band indicates that c-di-GMP alone cannot displace RamR. Data are representative of three independent experiments.
(TIF)

S1 Table LC-MS/MS identification of the c-di-GMP affinity-captured interactome. (XLSX)

S2 Table Genomic distribution and evolutionary conservation of UspG homologs. (XLSX)

S3 Table Transcriptomic profiling of differentially expressed genes in Δ*uspG* versus ATCC 700603 WT strains. (XLSX)

S4 Table Bacterial strains and plasmids utilized in this study. (DOCX)

S5 Table Oligonucleotide primers used in this study. (DOCX)

S1 Rawgel Original uncropped raw images for all electrophoretic gels and immunoblots in this study. (PDF)
