# Supplementary material for: Cyclic di-GMP directly reprograms the multidrug resistance machinery via UspG-mediated sequestration of RamR in Klebsiella
Source: PLoS Pathog. 2026 Aug 24;22(8):e1014537. doi: 10.1371/journal.ppat.1014537 (PMC13524336; doi:10.1371/journal.ppat.1014537)

Figure1D_raw_image

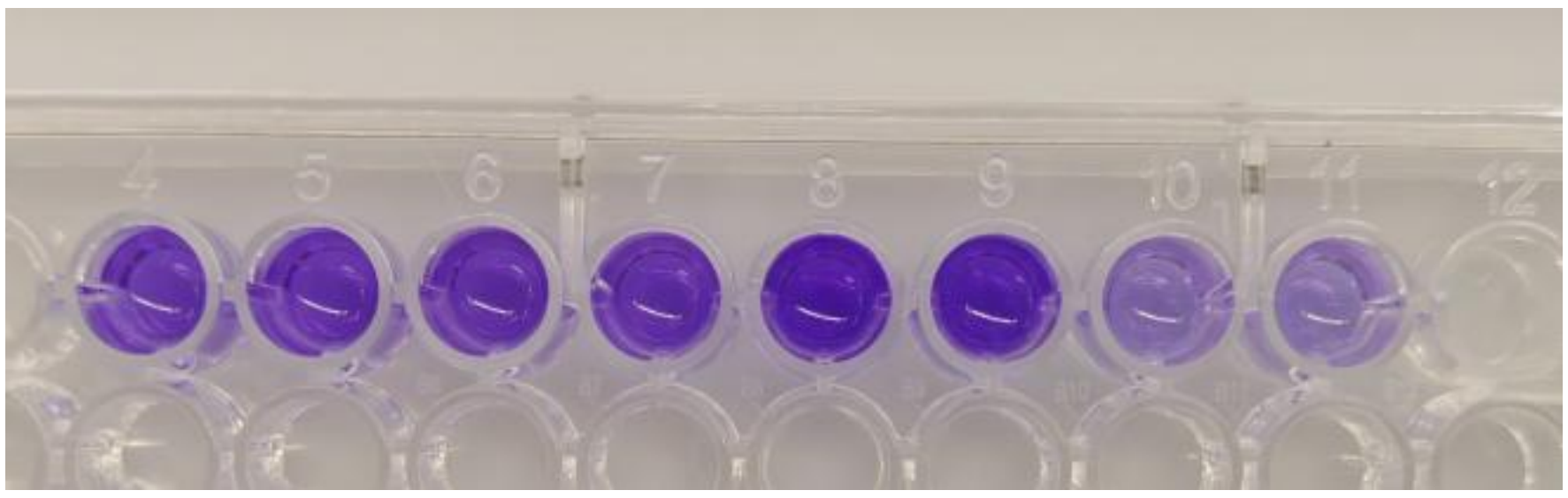

Figure1F_raw_image

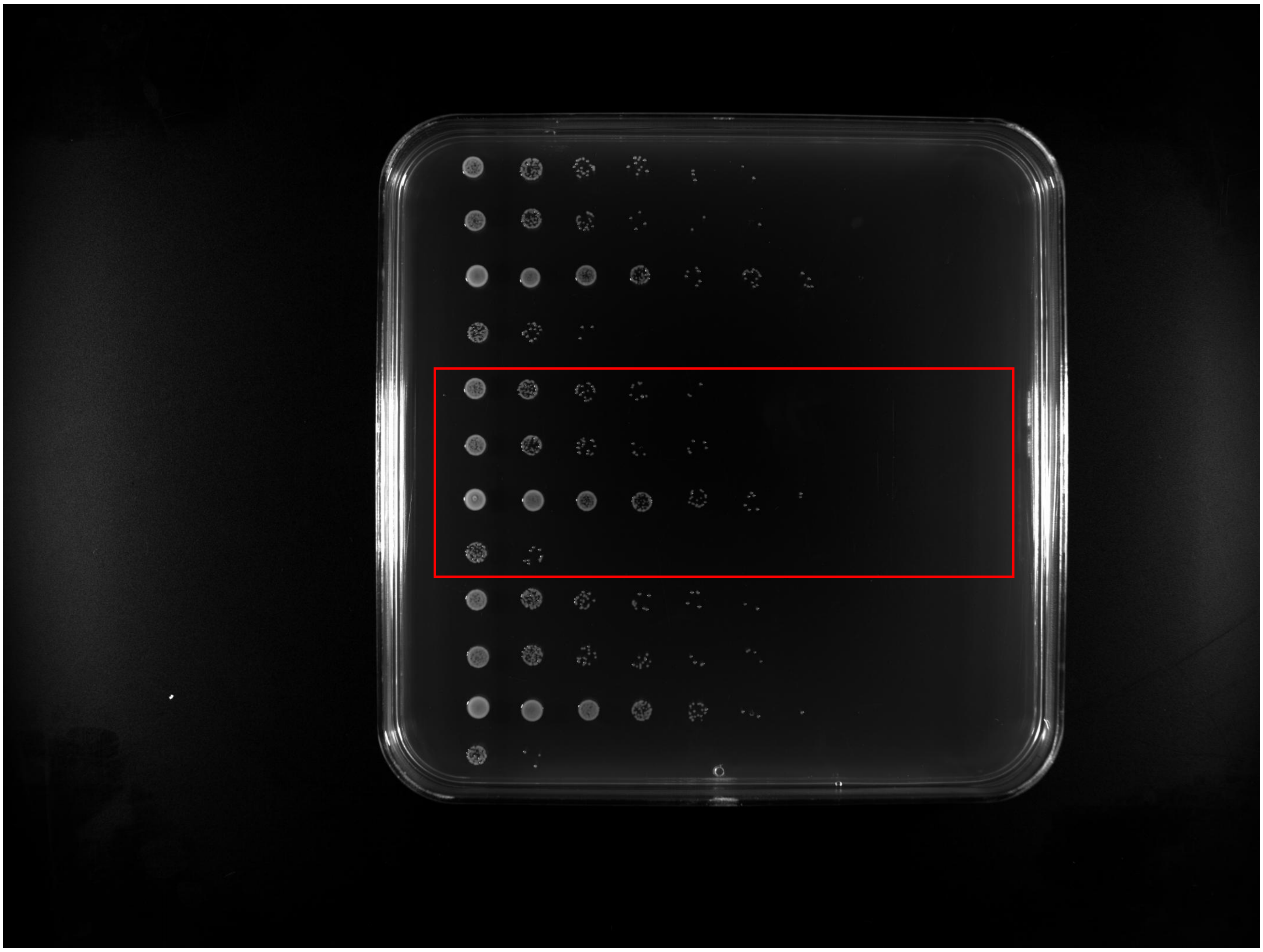

Figure1G_raw_image

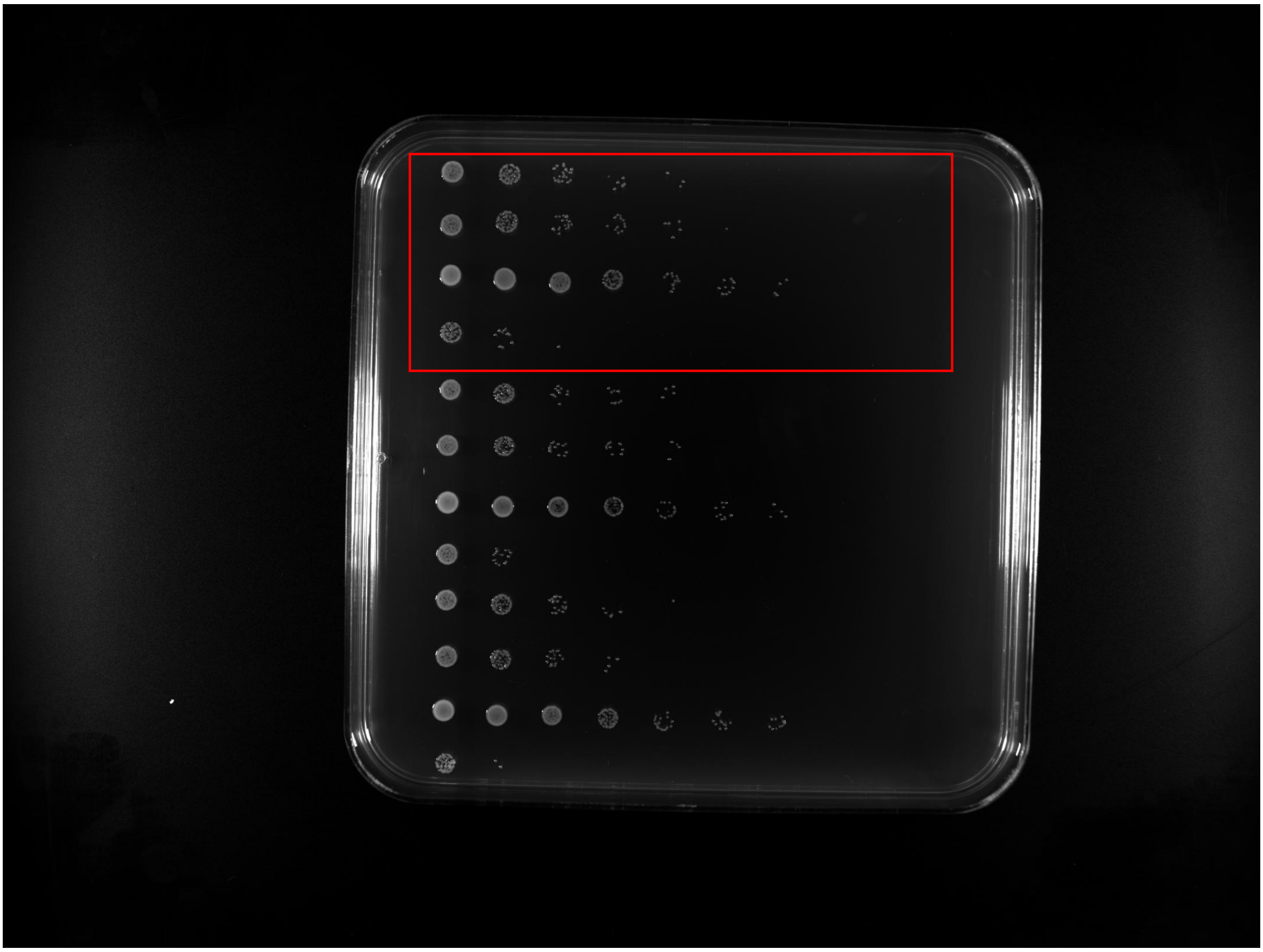

Figure2A_raw_image

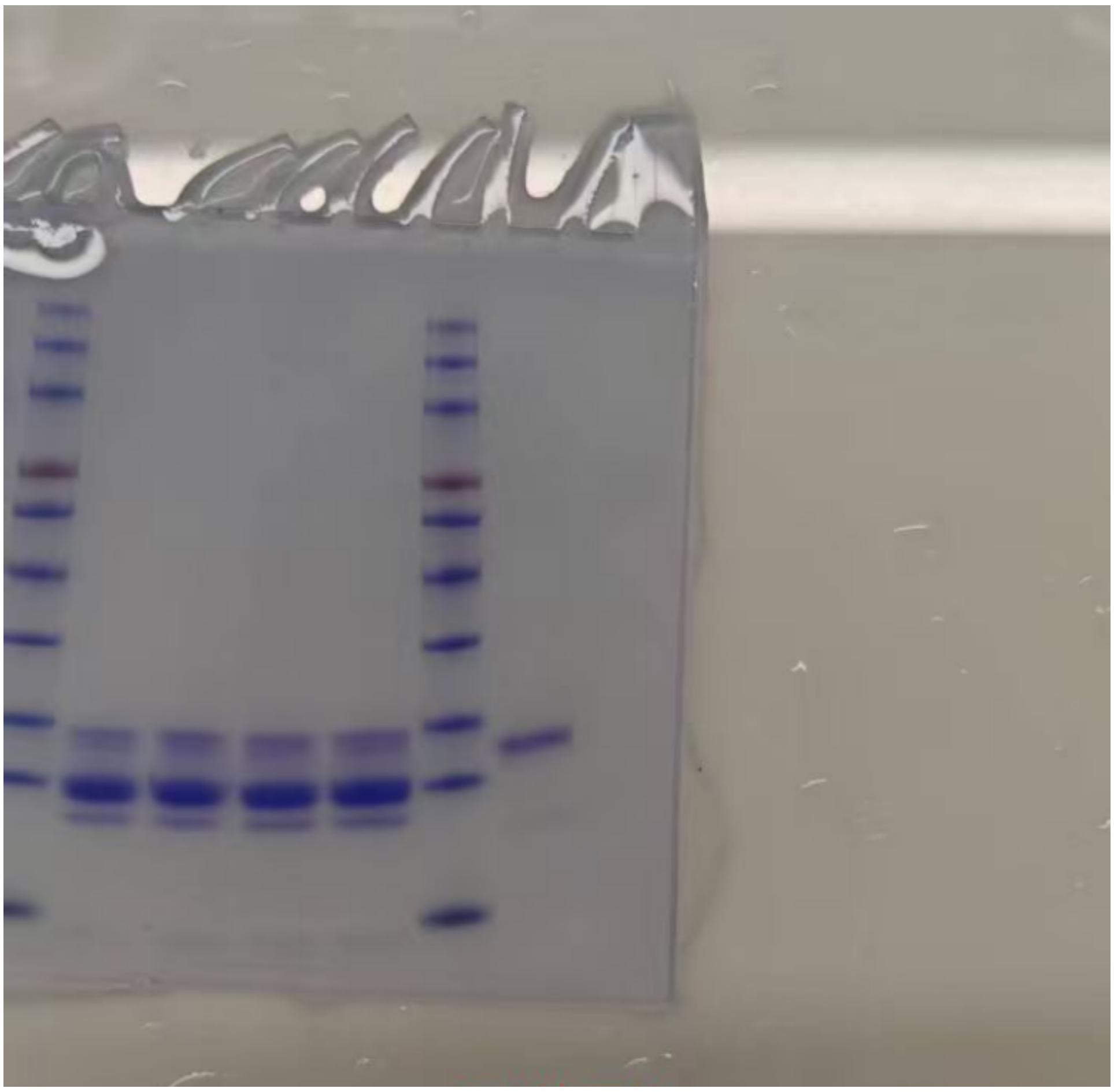

Figure2F_raw_image

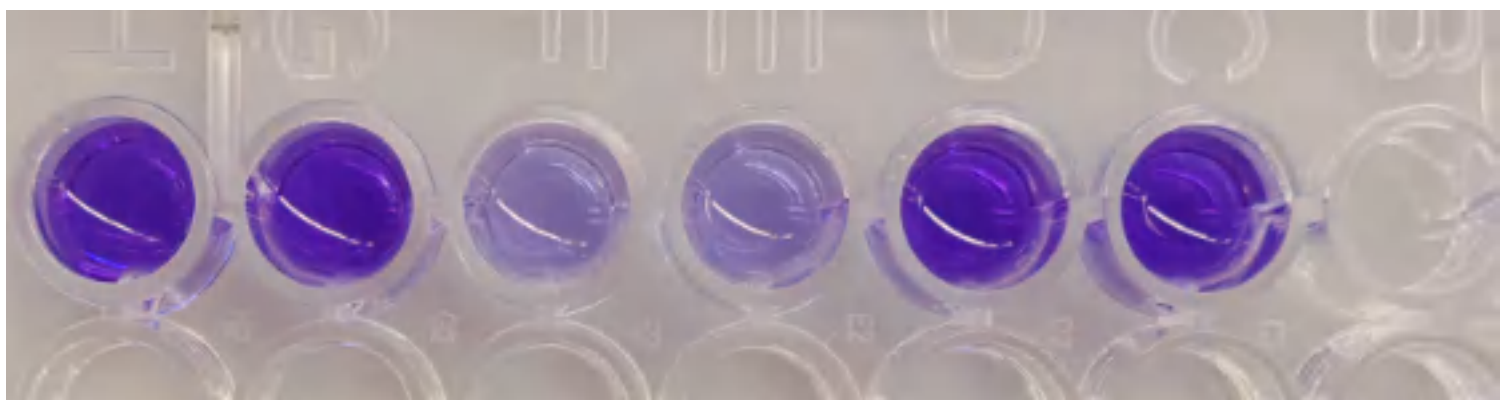

Figure2G_raw_image

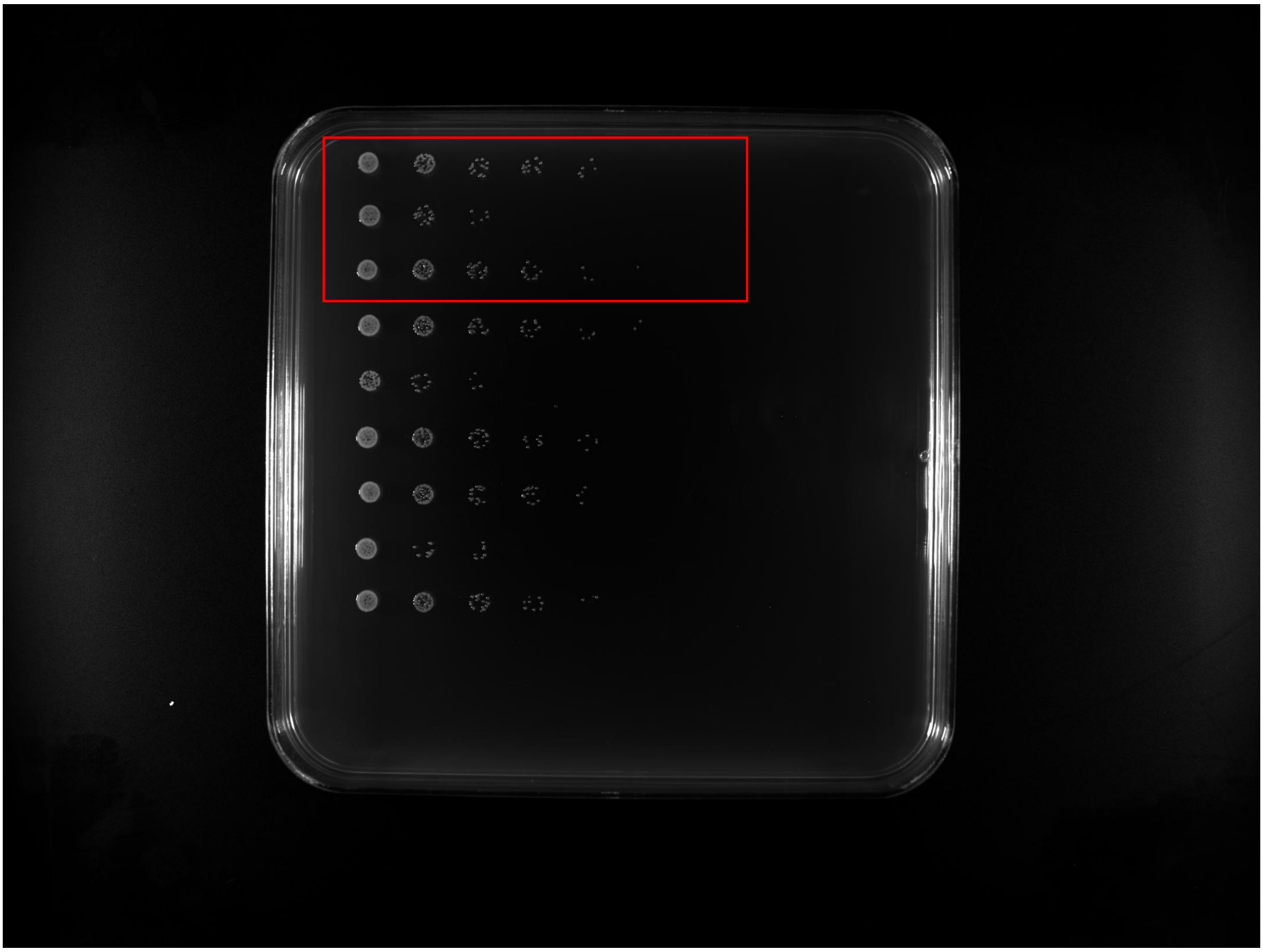

Figure2H_raw_image

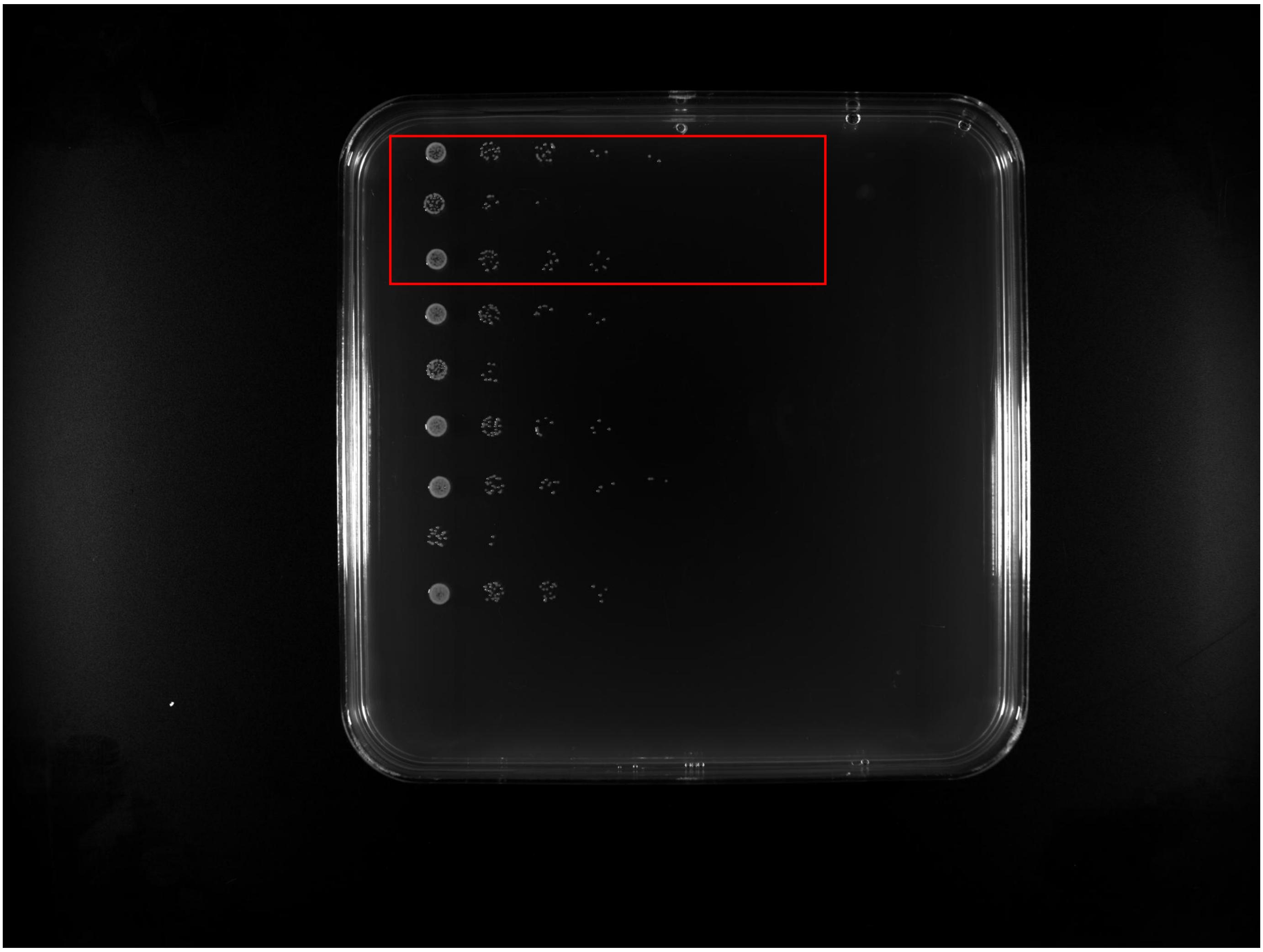

Figure3D_raw_image

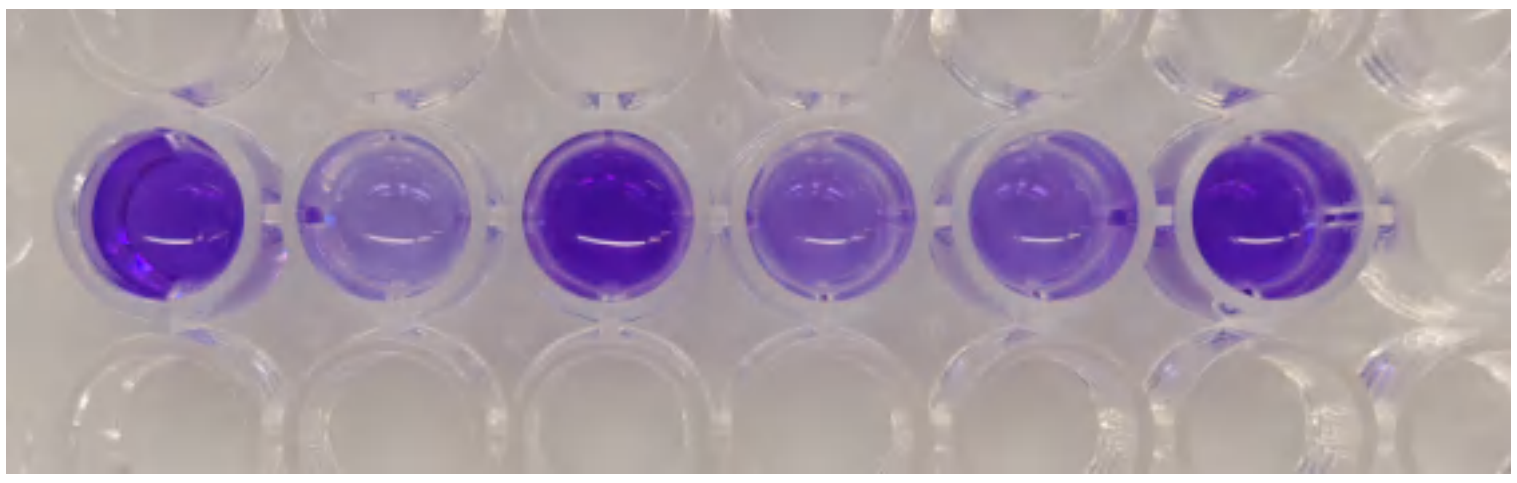

Figure3E_raw_image

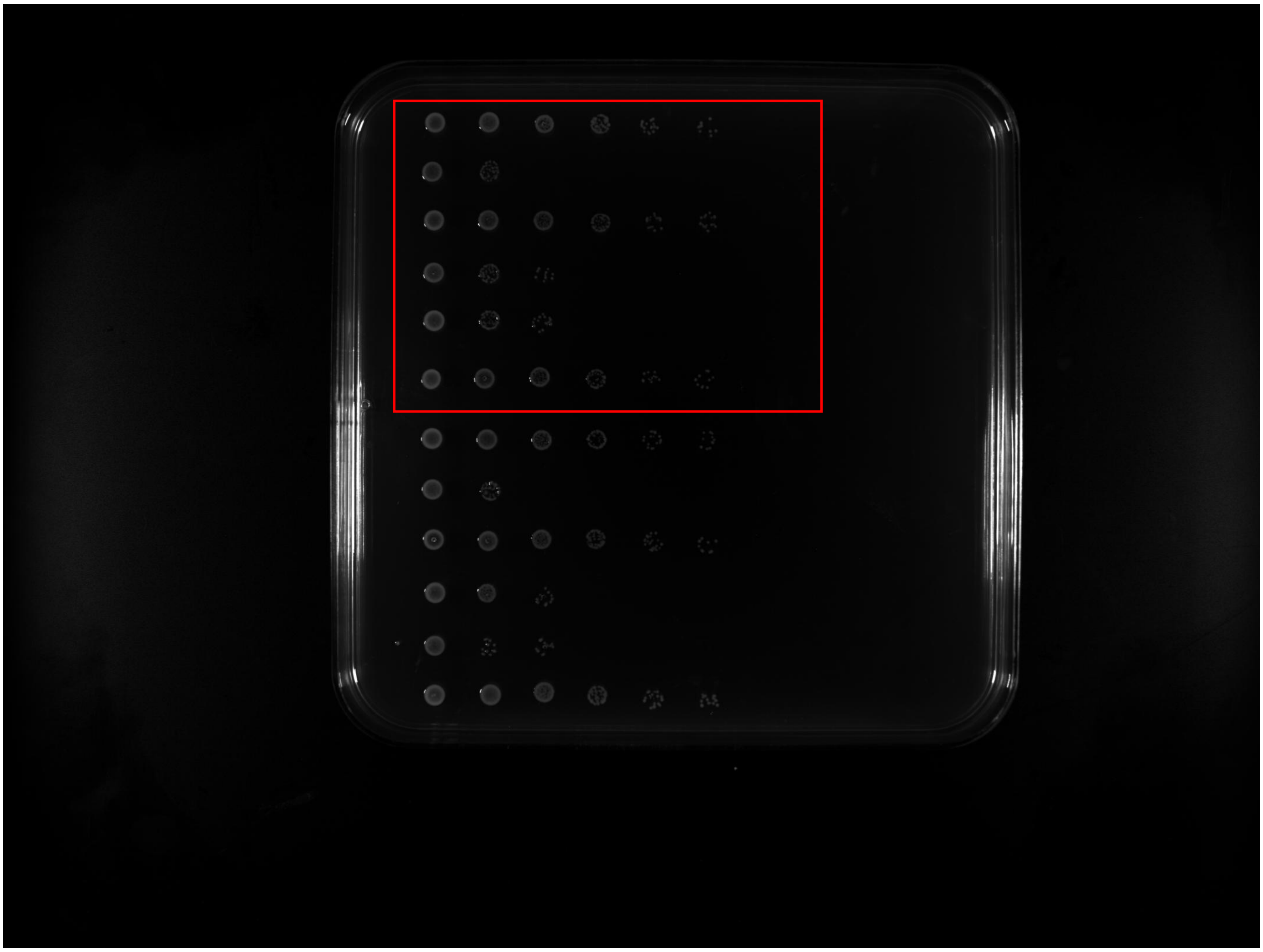

Figure3E_raw_image

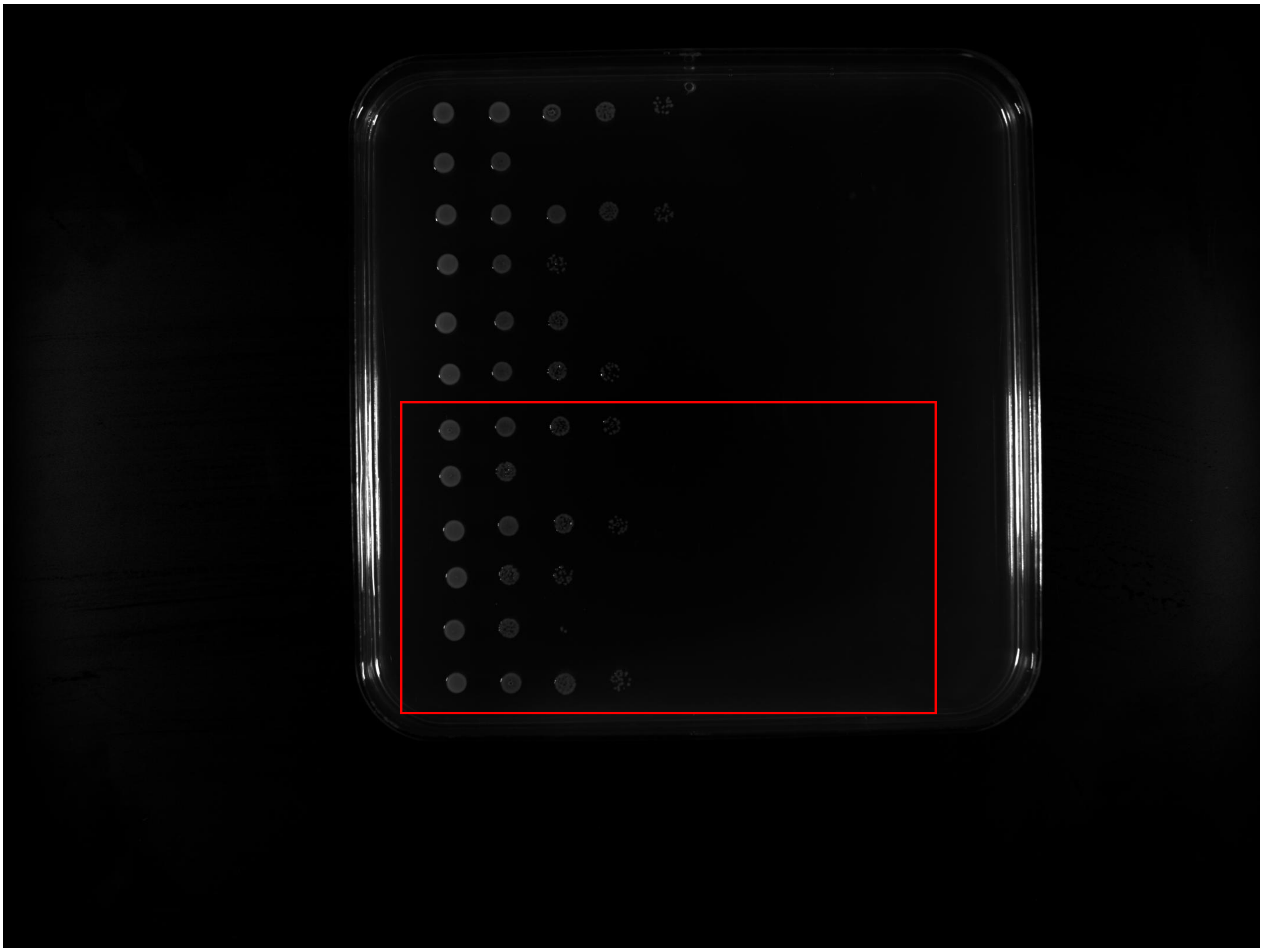

Figure4F_raw_image

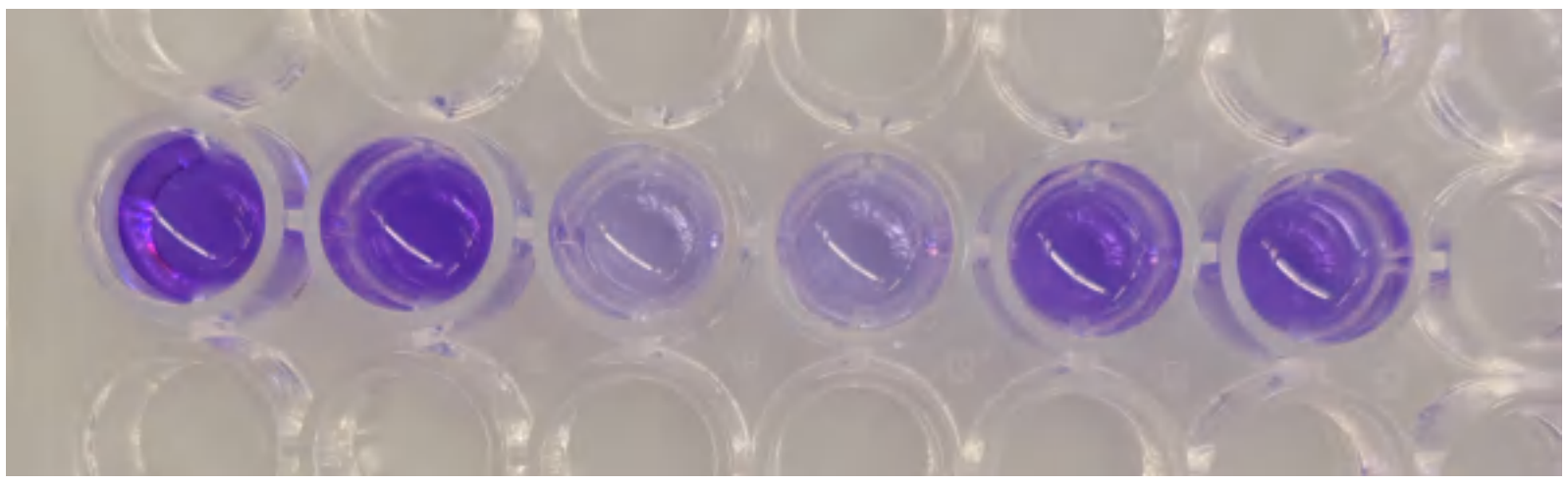

Figure4G_raw_
image

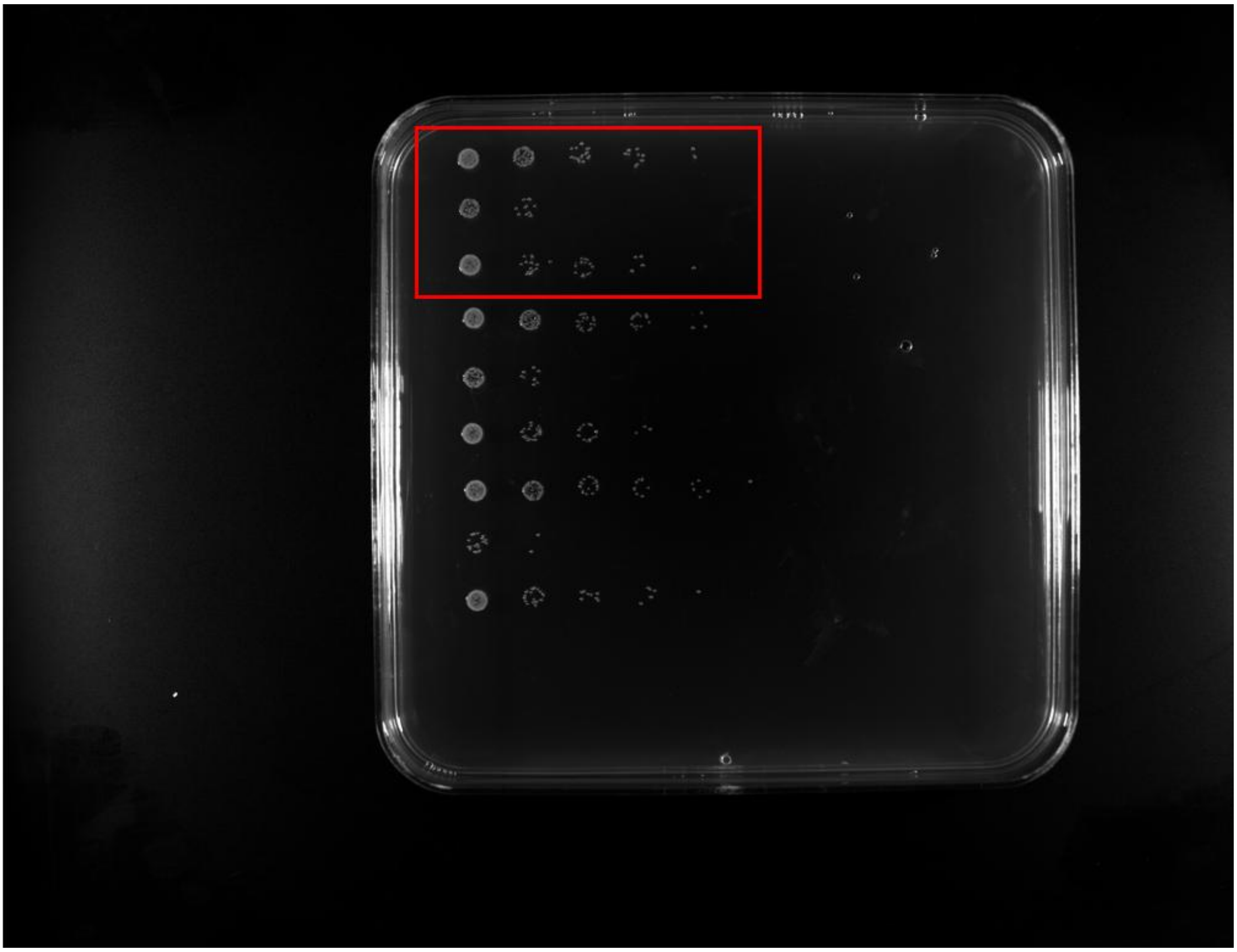

Figure4H_raw_image

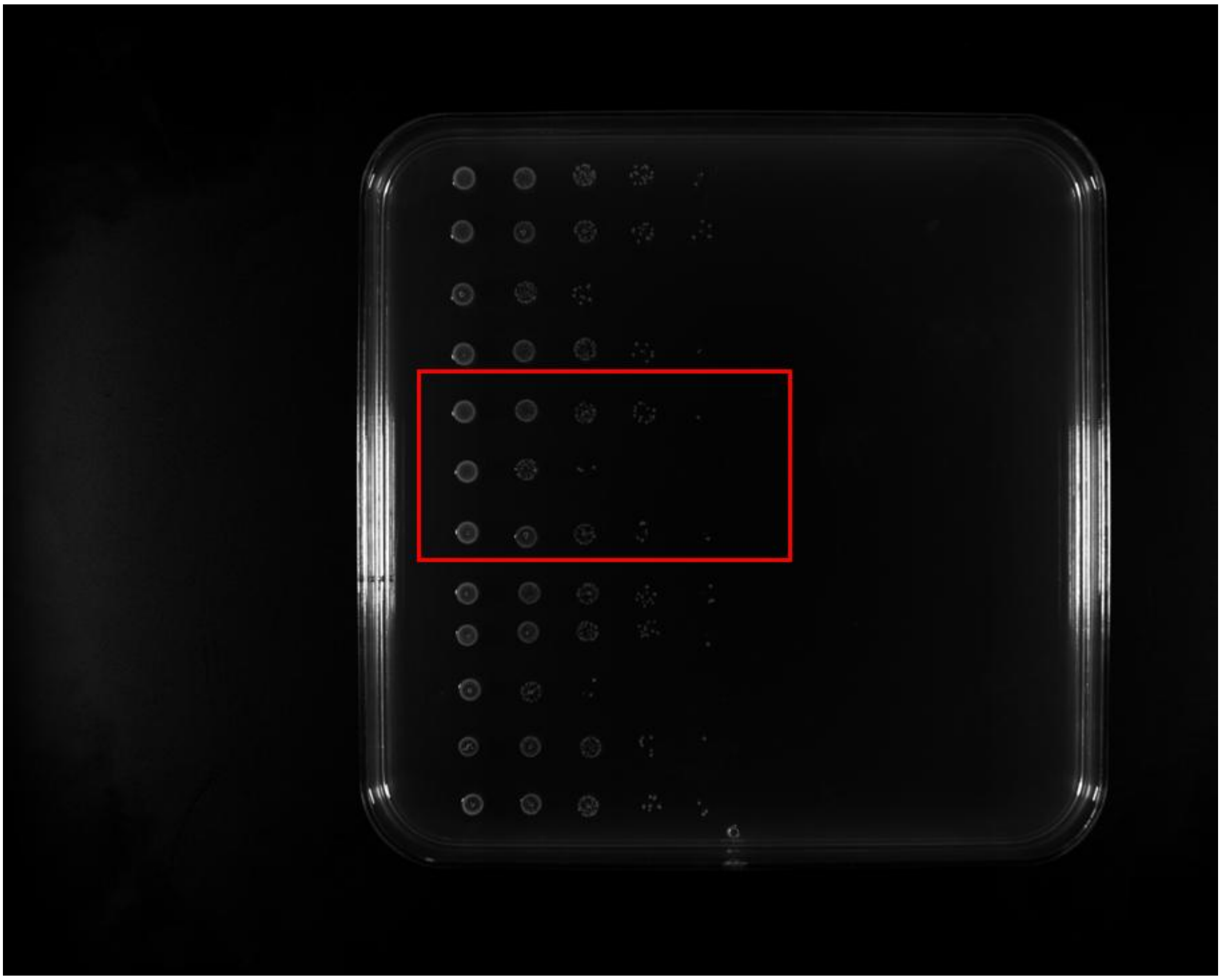

Figure5B_raw_image

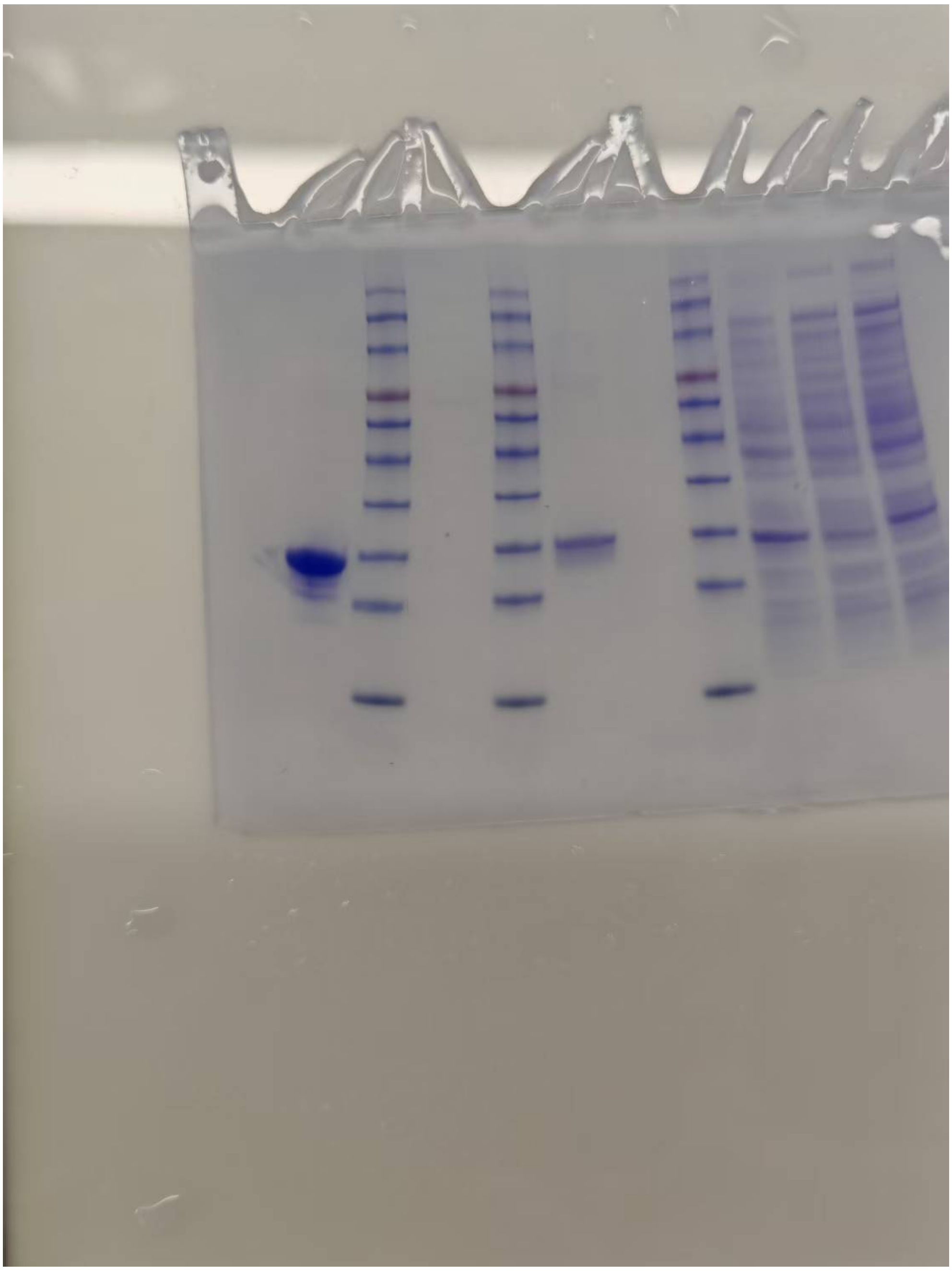

Figure5C_raw_image

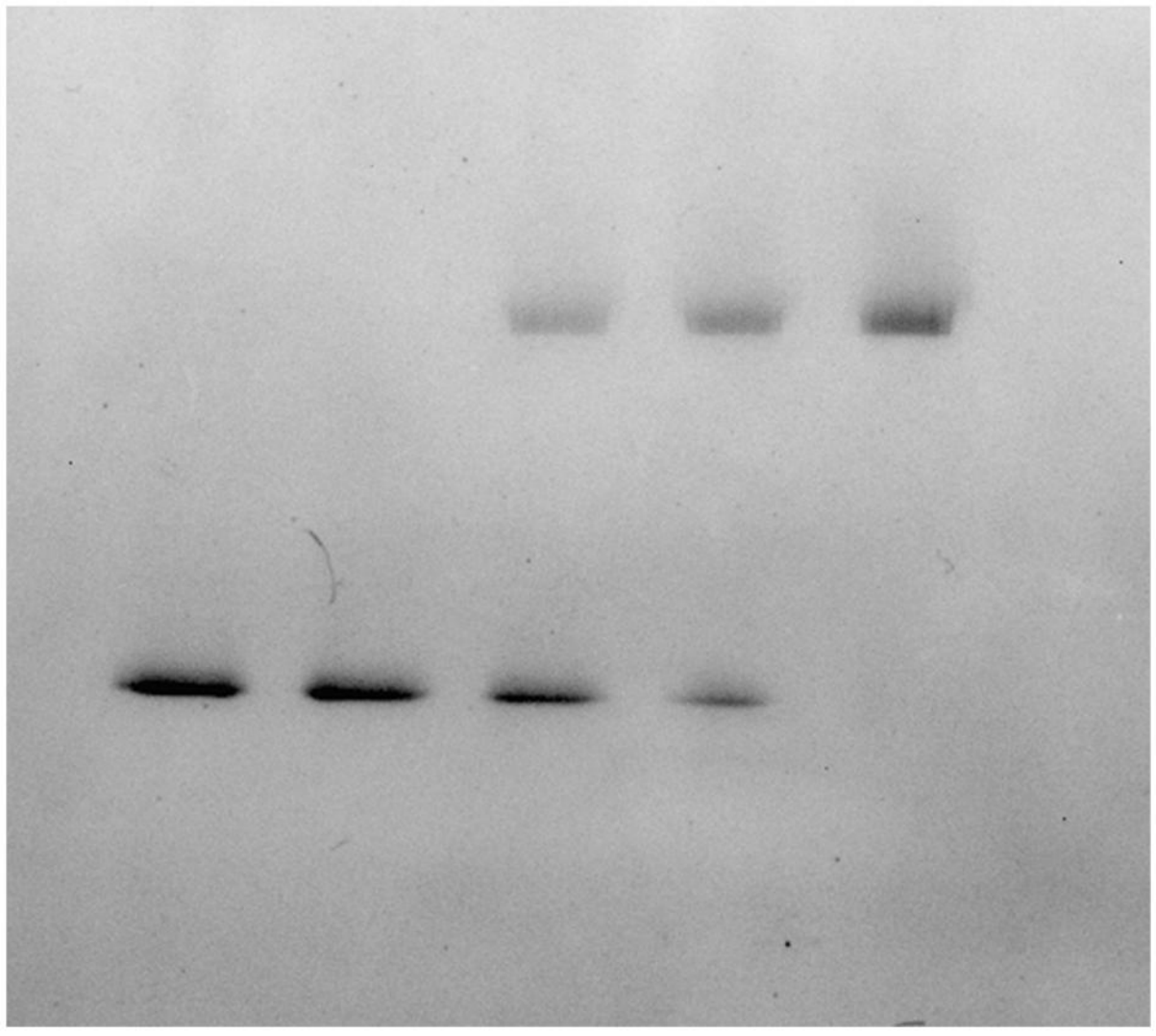

Figure5F_raw_image

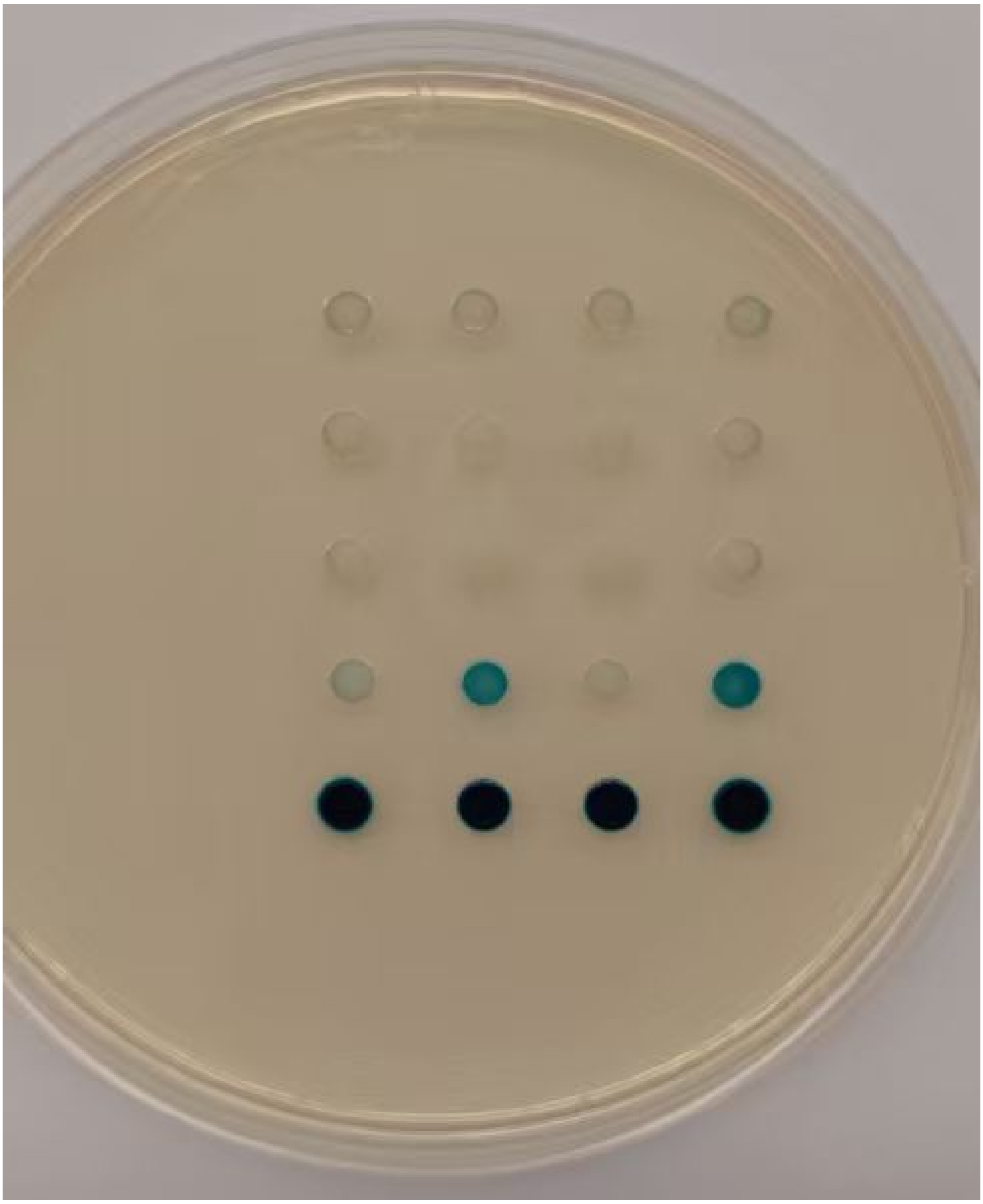

Figure5G_raw_image

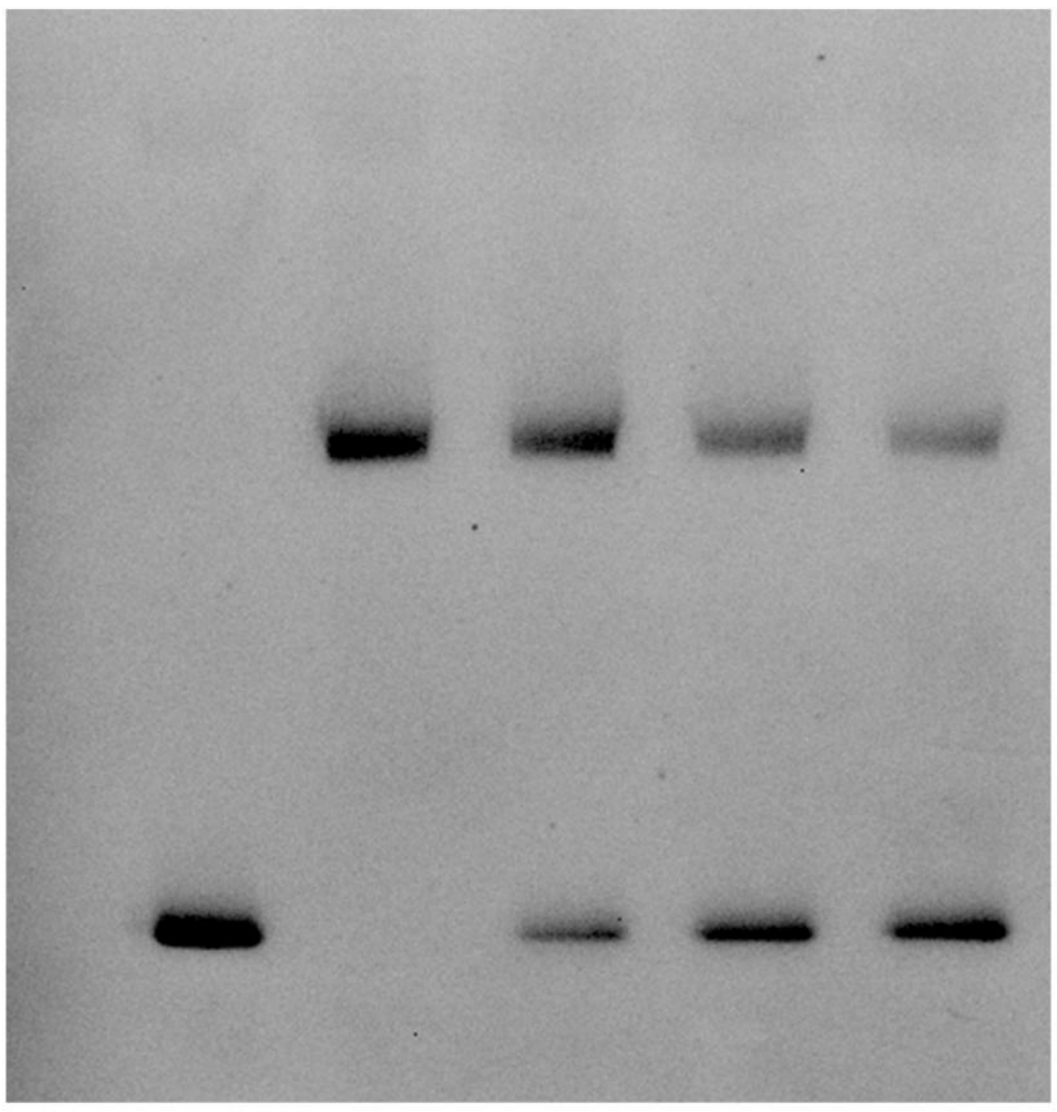

Figure6C_raw_image

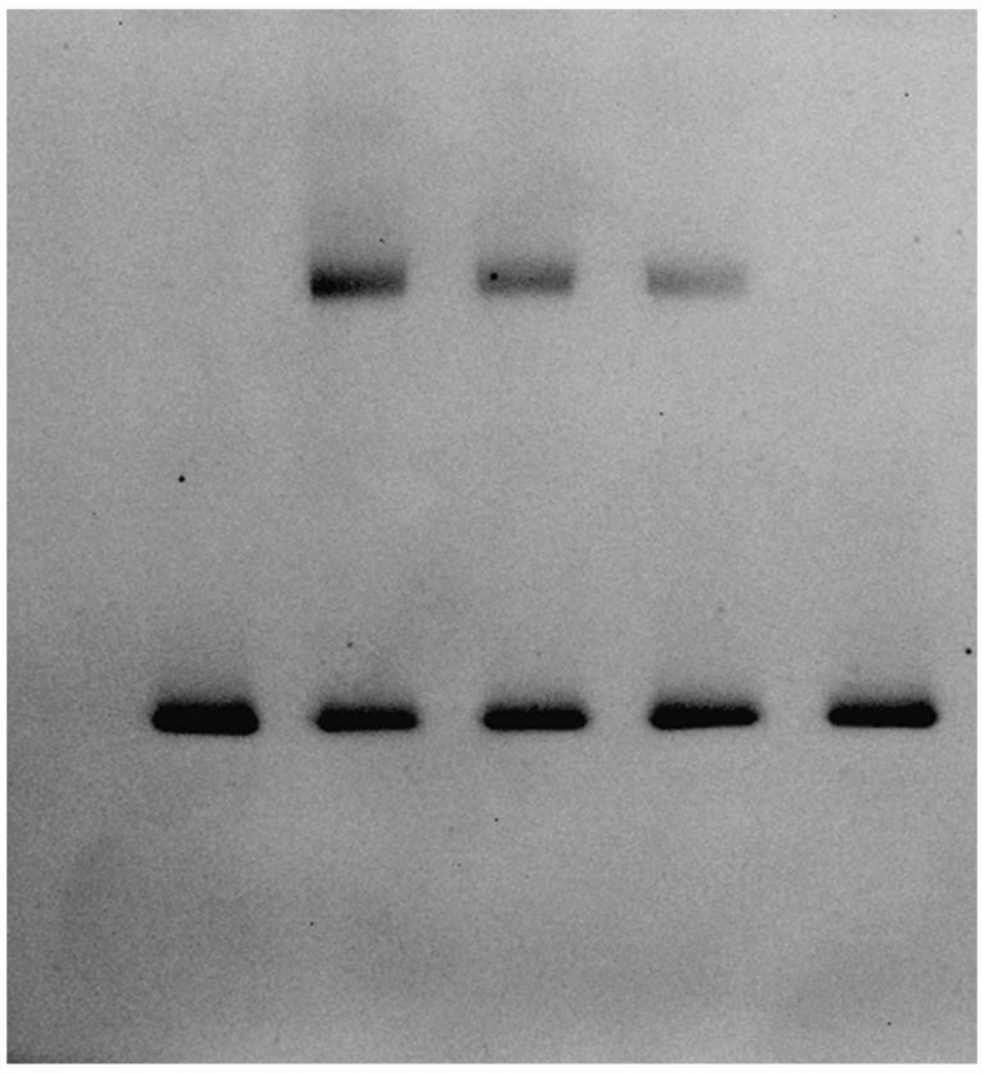

Figure6D_raw_image

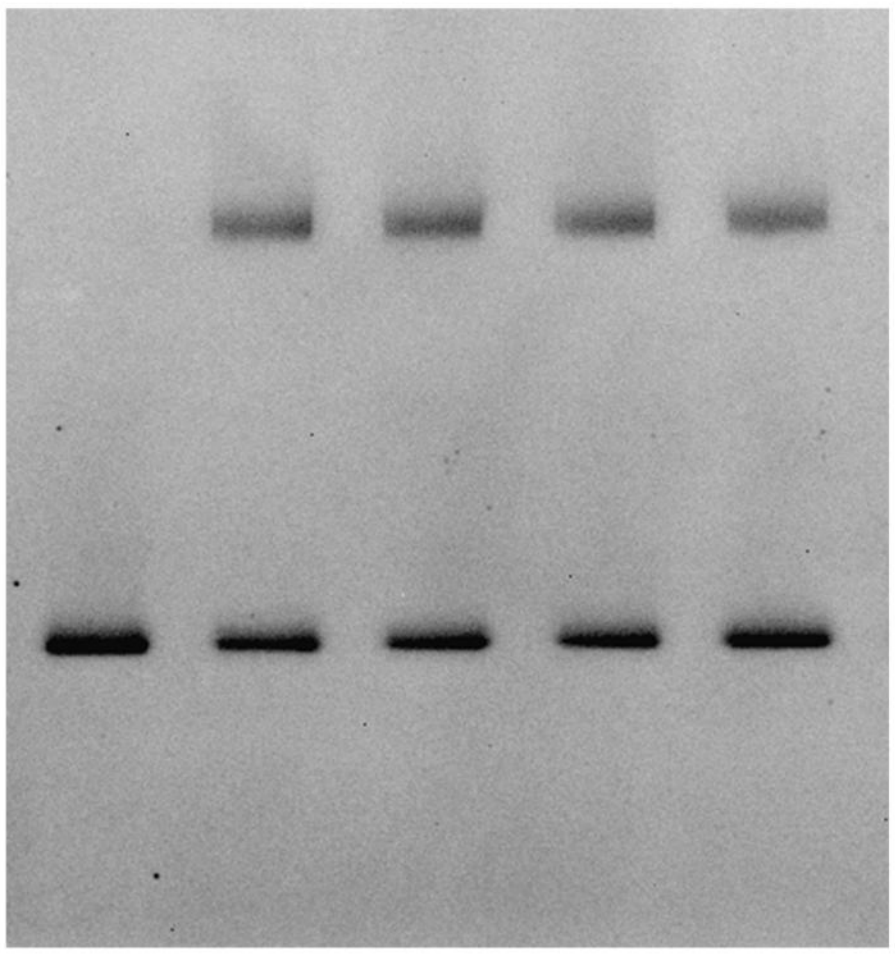

Figure6E_raw_image

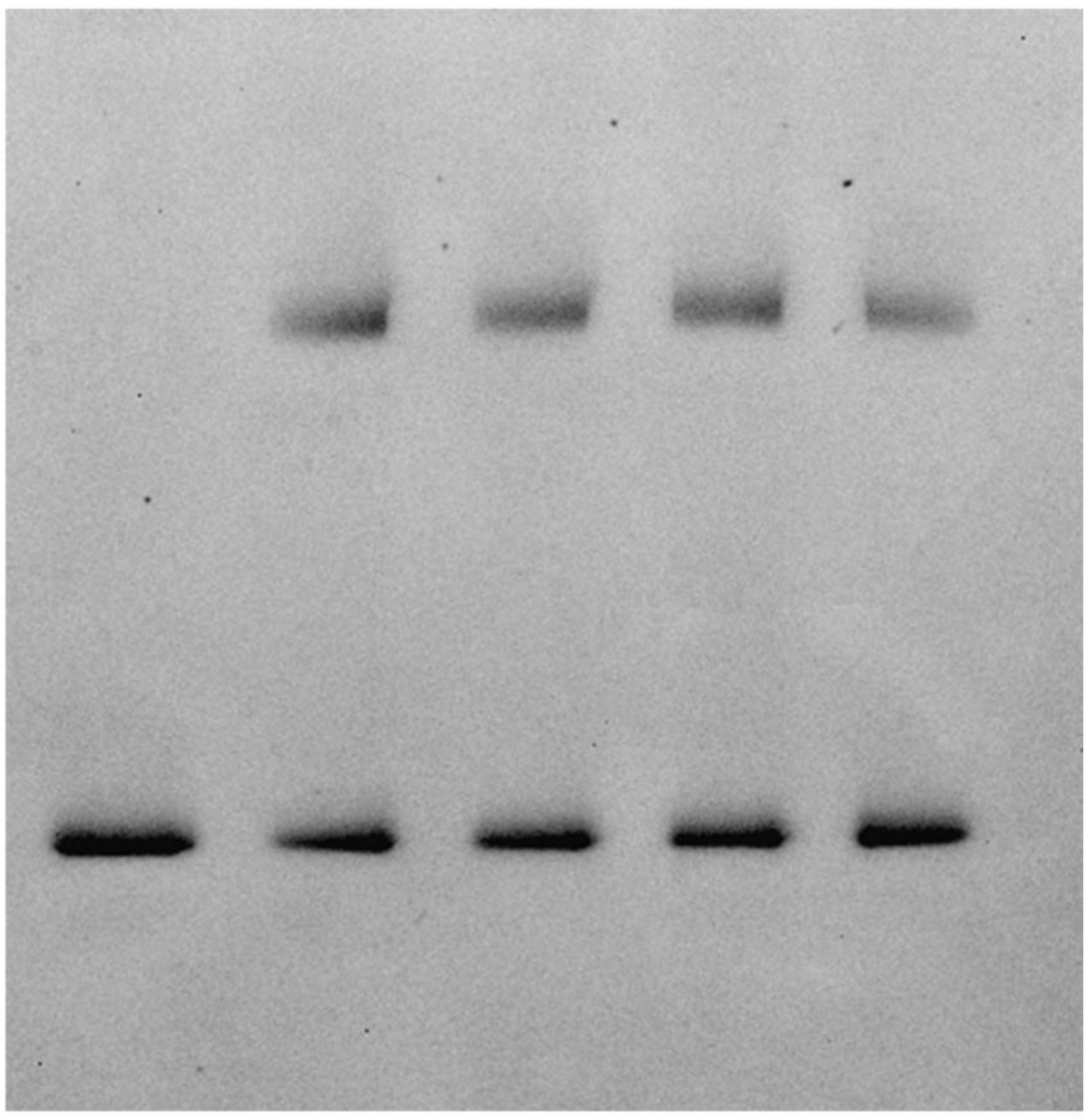

Figure6F_raw_image

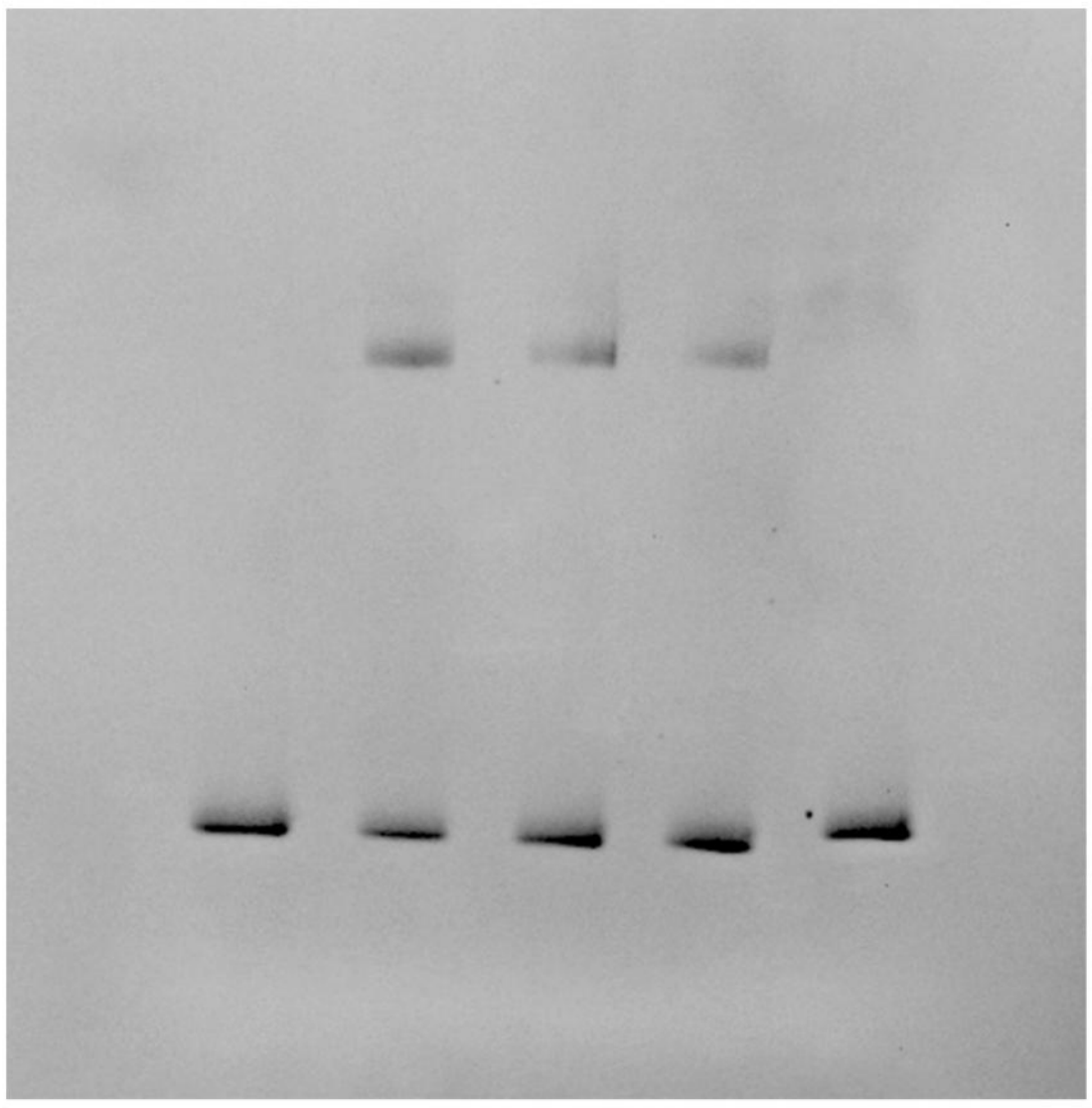

Supplementary_Fig_S1B (KAN)_ raw_image

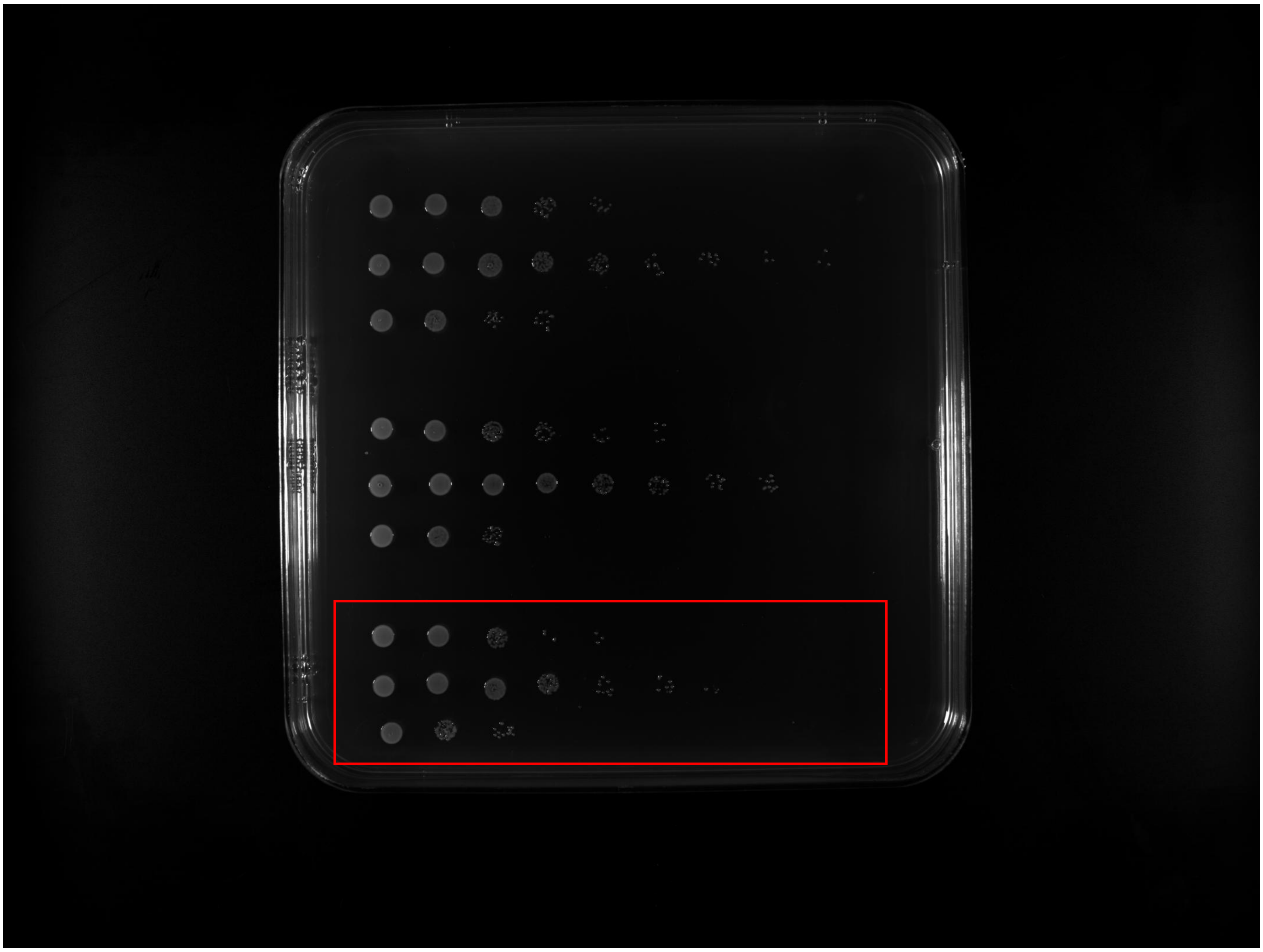

Supplementary_Fig_S1B(Tet)_ raw_image

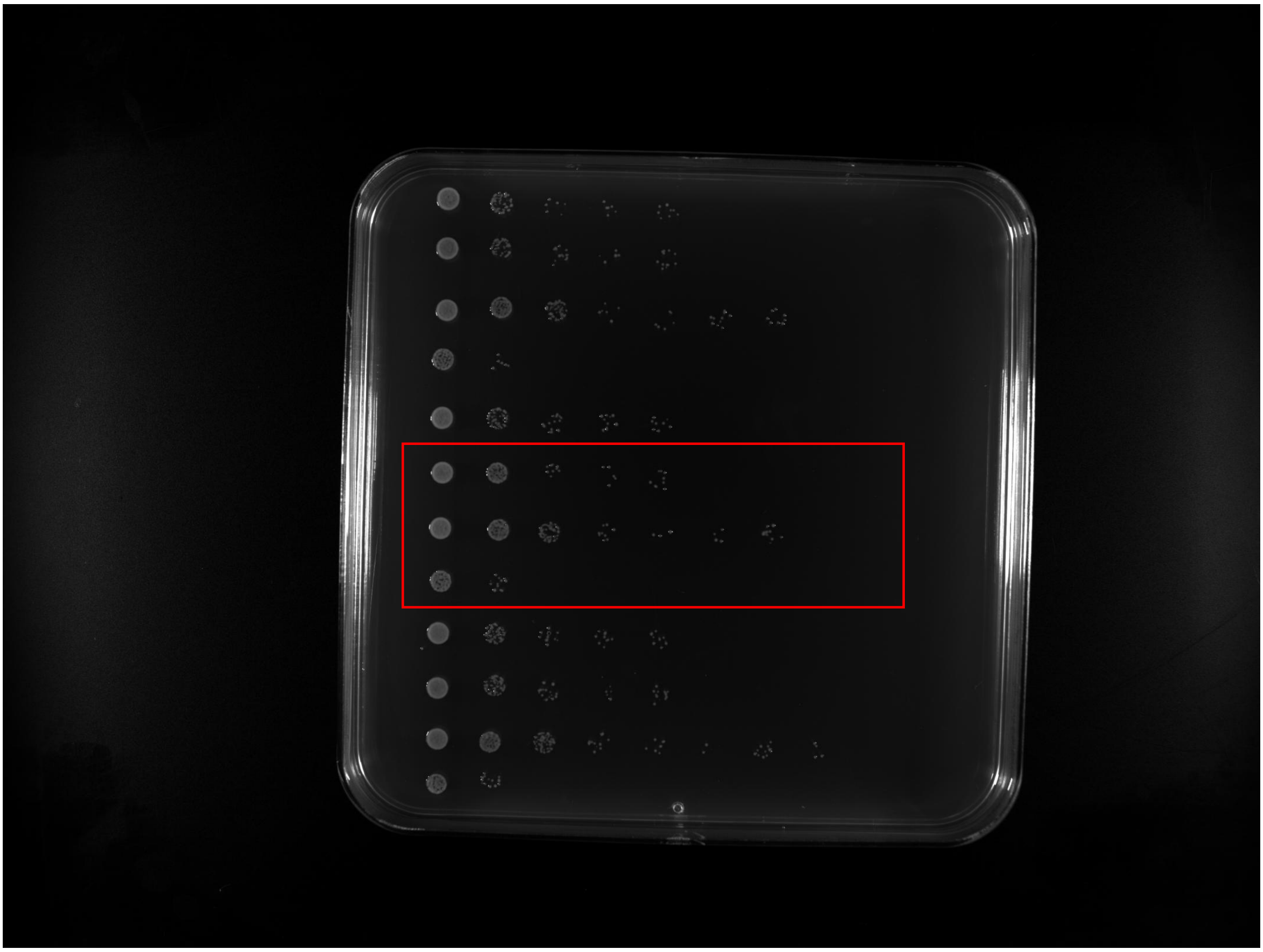

Supplementary_Fig_S2A_ raw_image

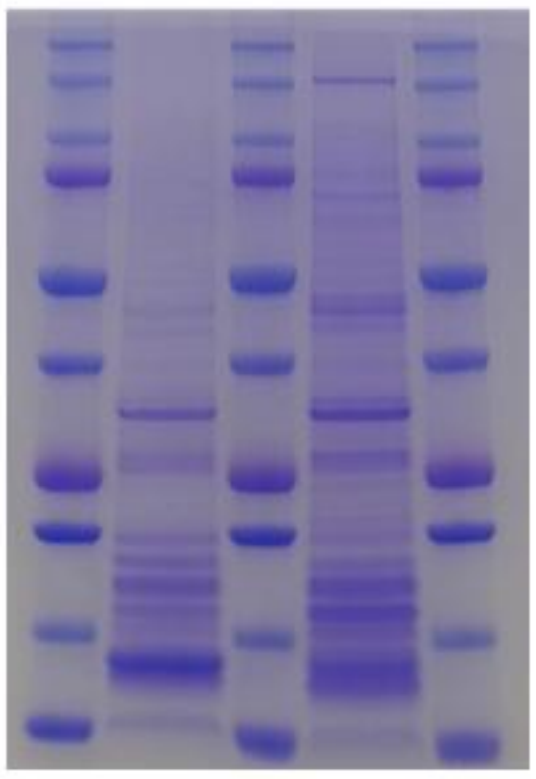

Supplementary_Fig_S4A_ raw_image

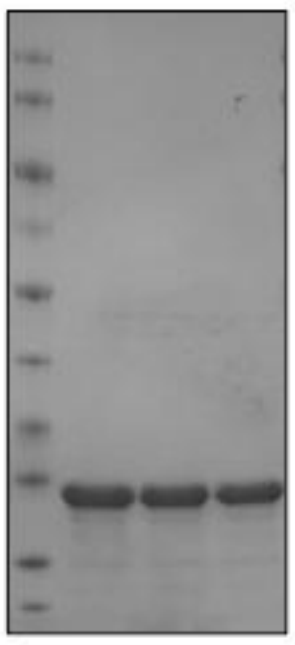

Supplementary_Fig_S4B_ raw_image

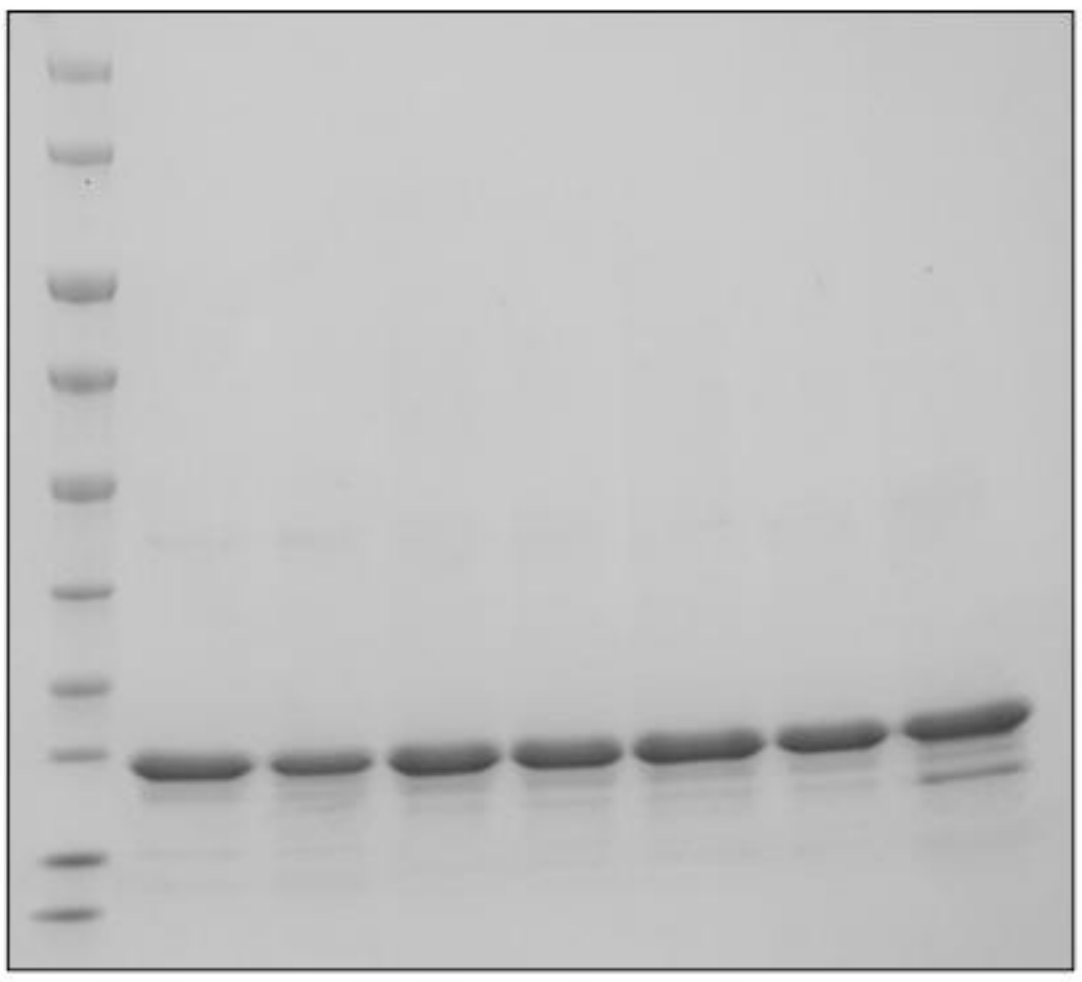

Supplementary_Fig_S5A_ raw_image

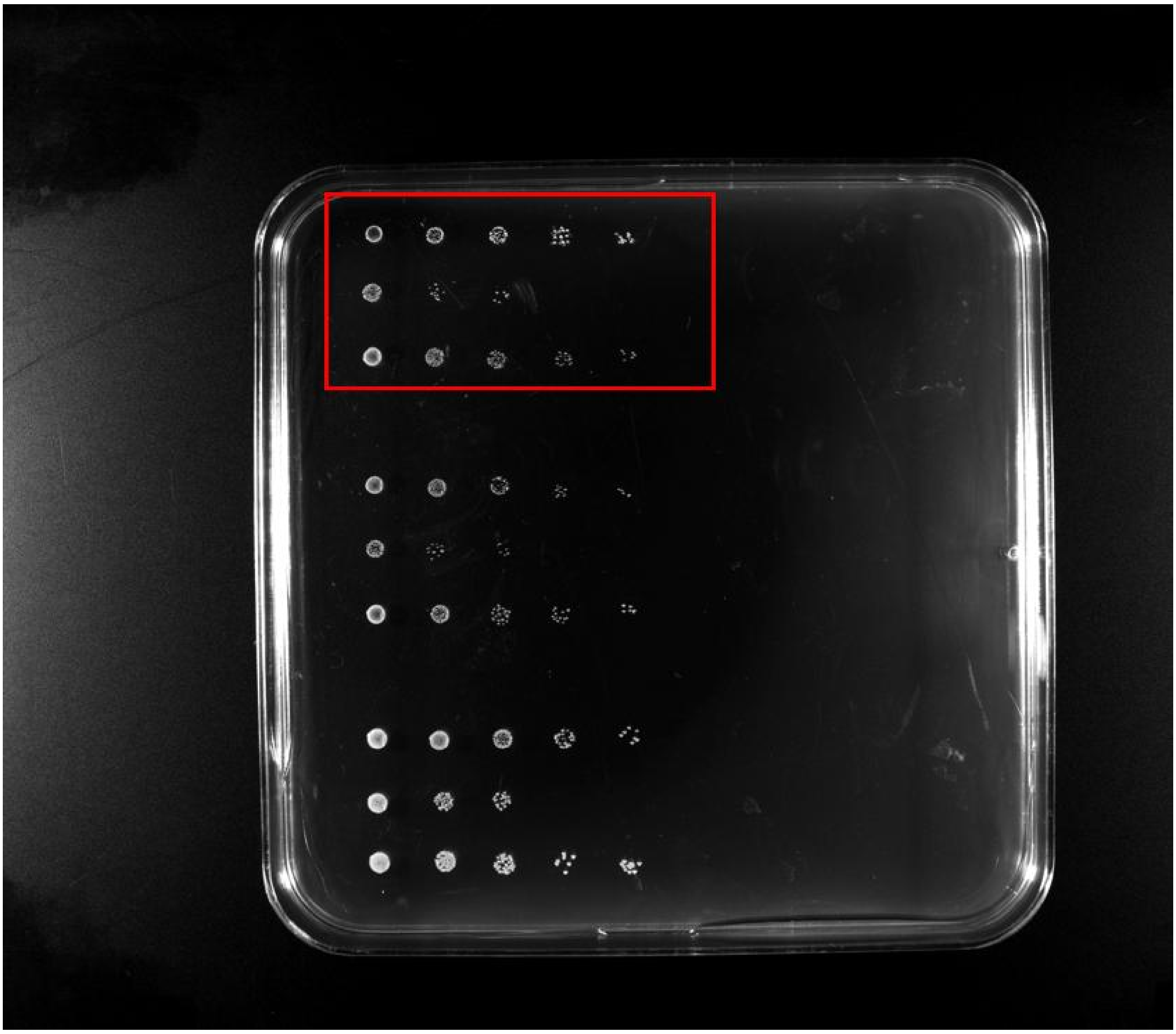

Supplementary_Fig_S5B_ raw_image

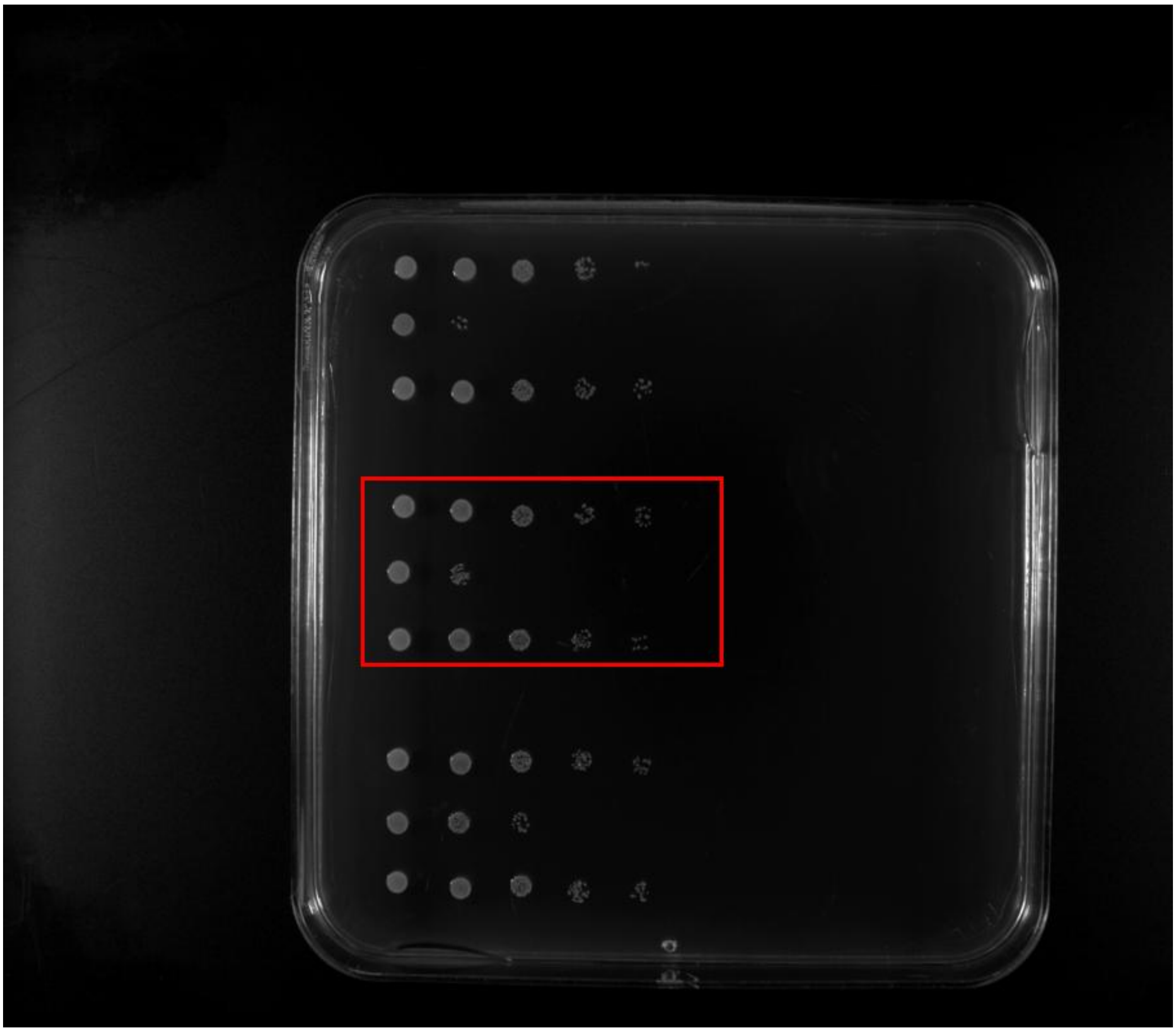

Supplementary_Fig_S5E_ raw_image

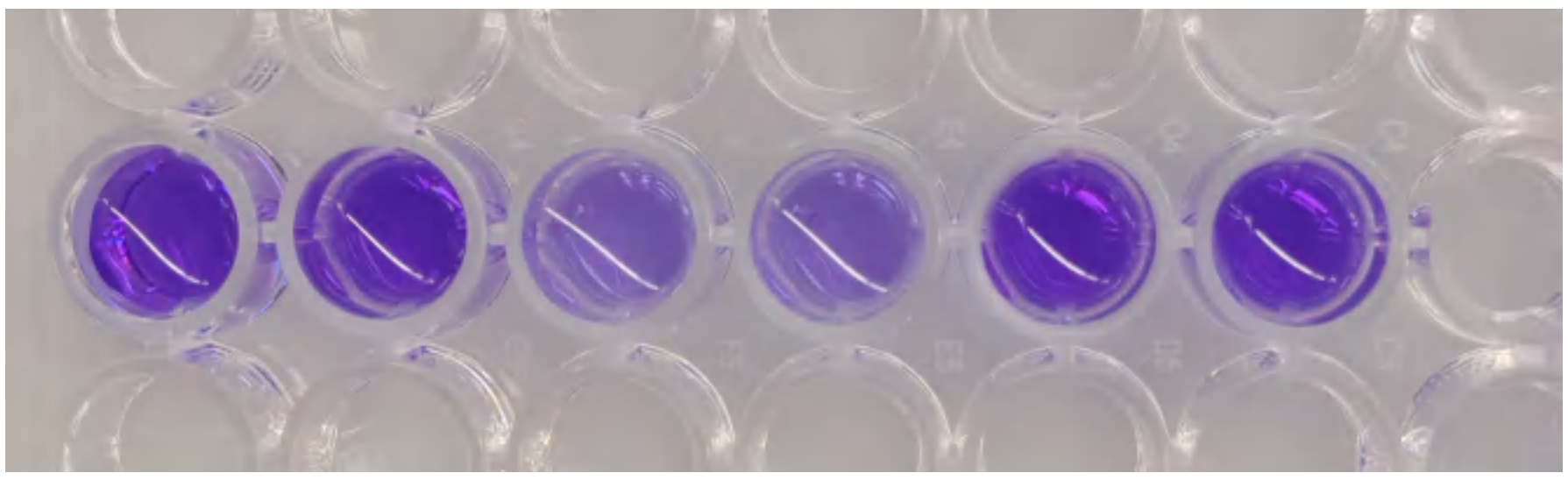

Supplementary_Fig_S5F_ raw_image

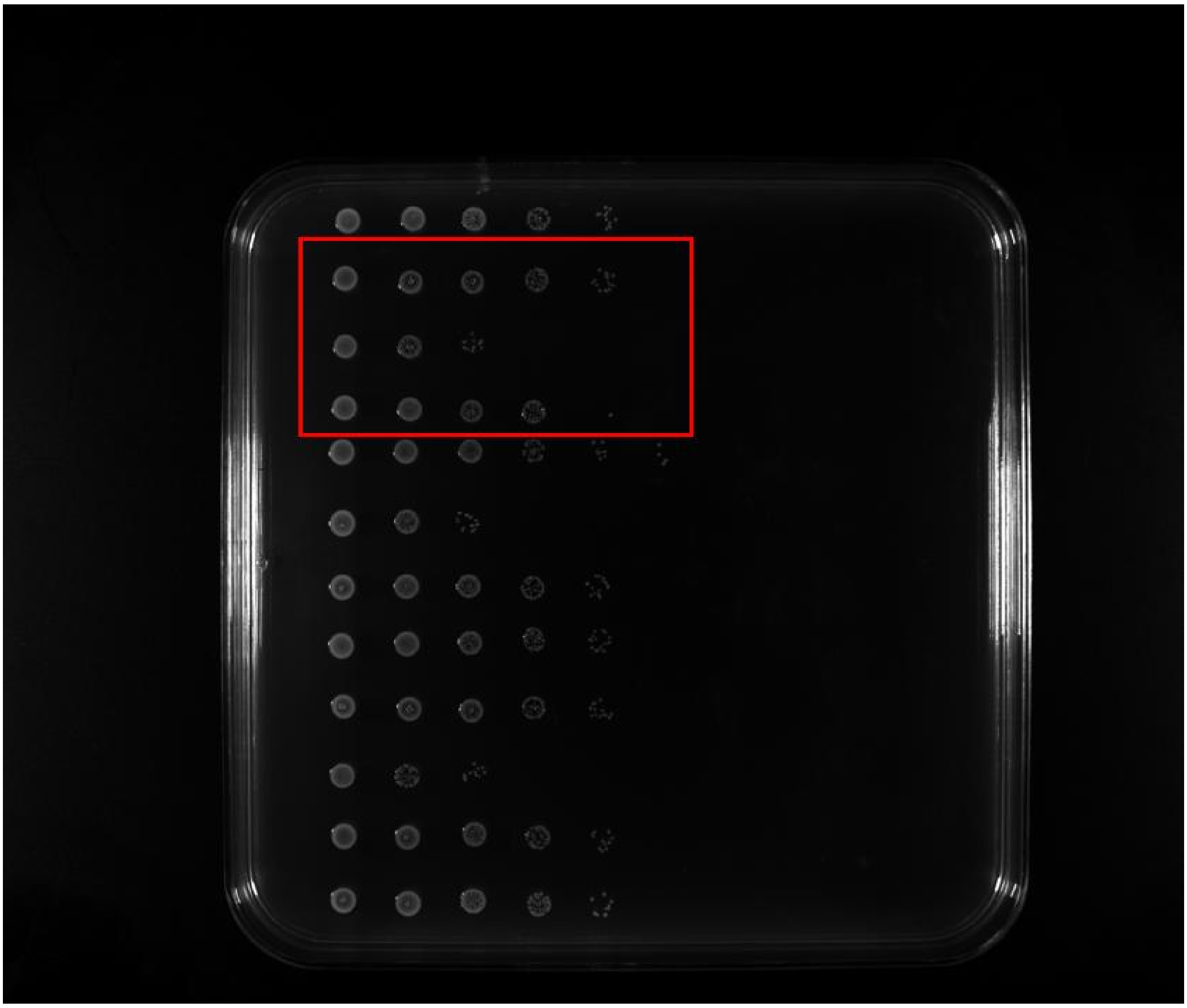

Supplementary_Fig_S5G_ raw_
image

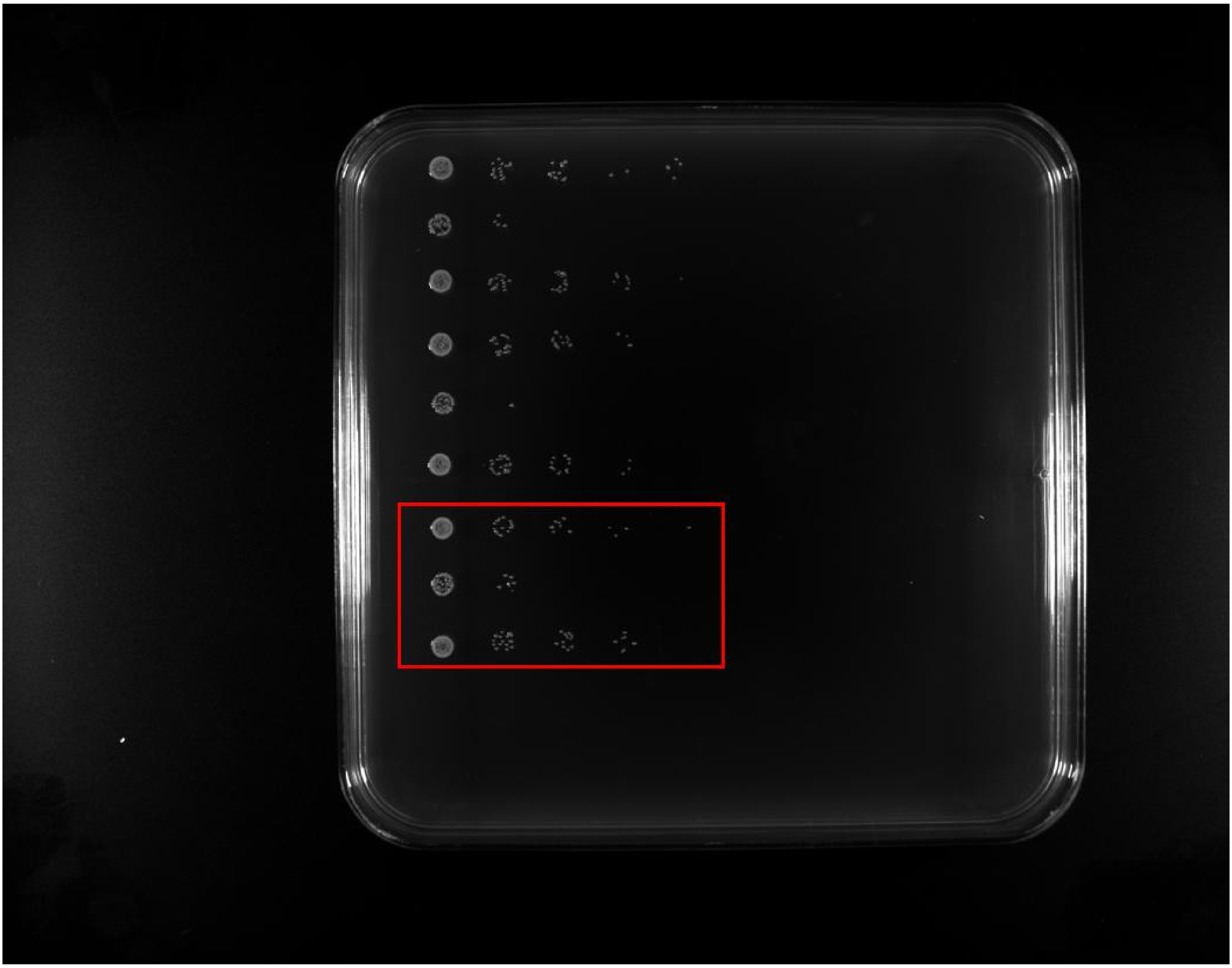

Supplementary_Fig_S5H_ raw_
image

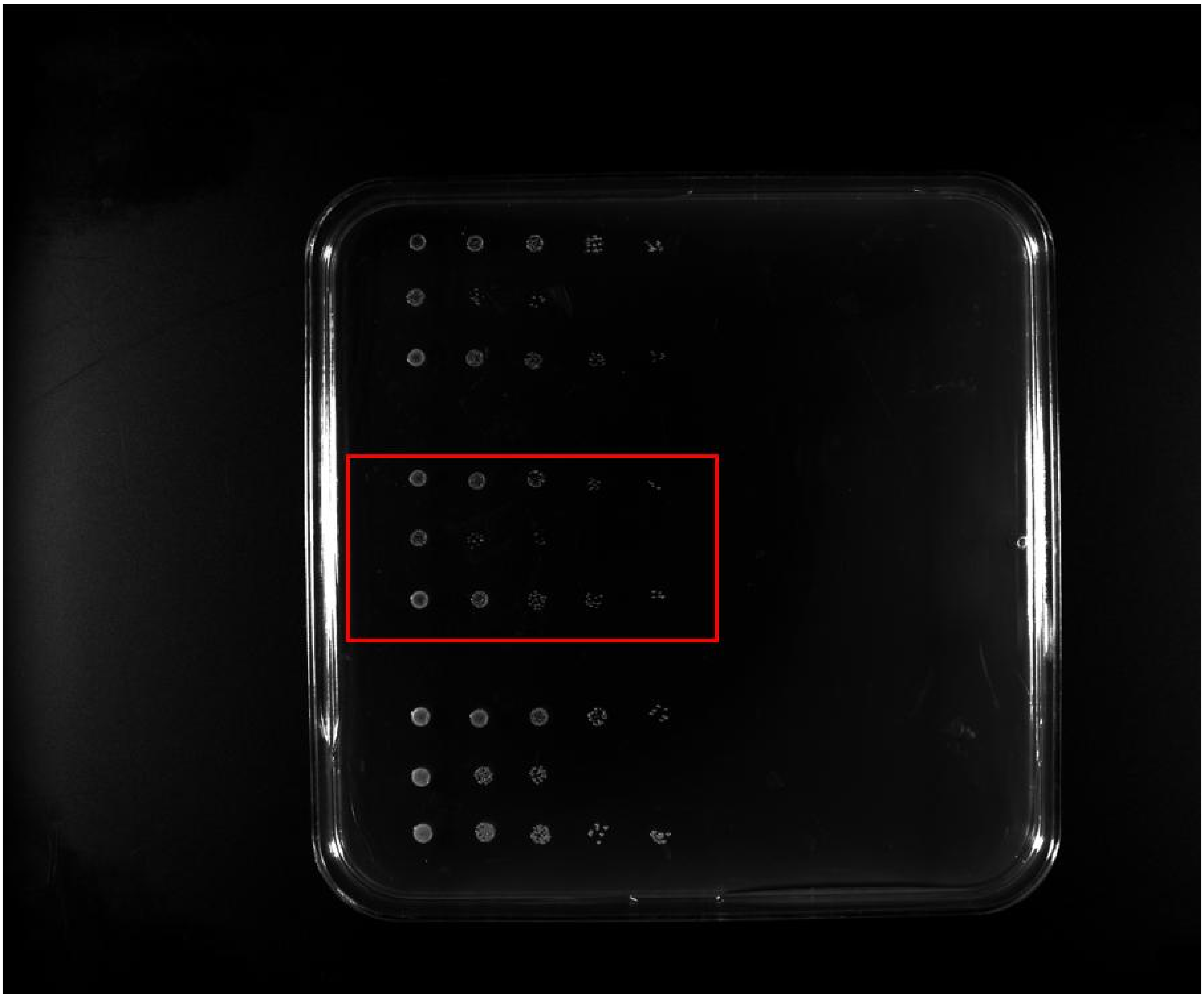

Supplementary_Fig_S5I_ raw_image

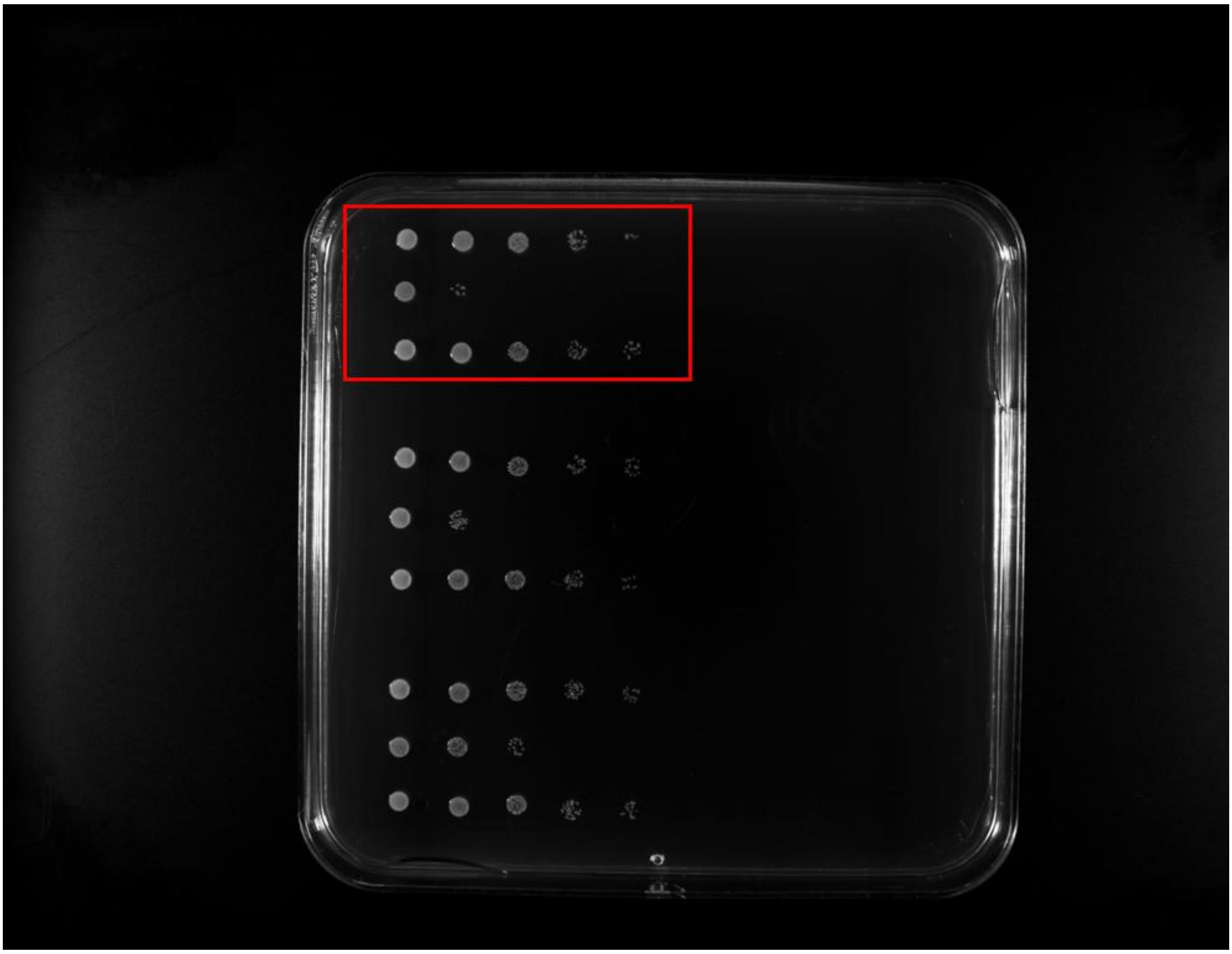

Supplementary_Fig_S7A_ raw_image

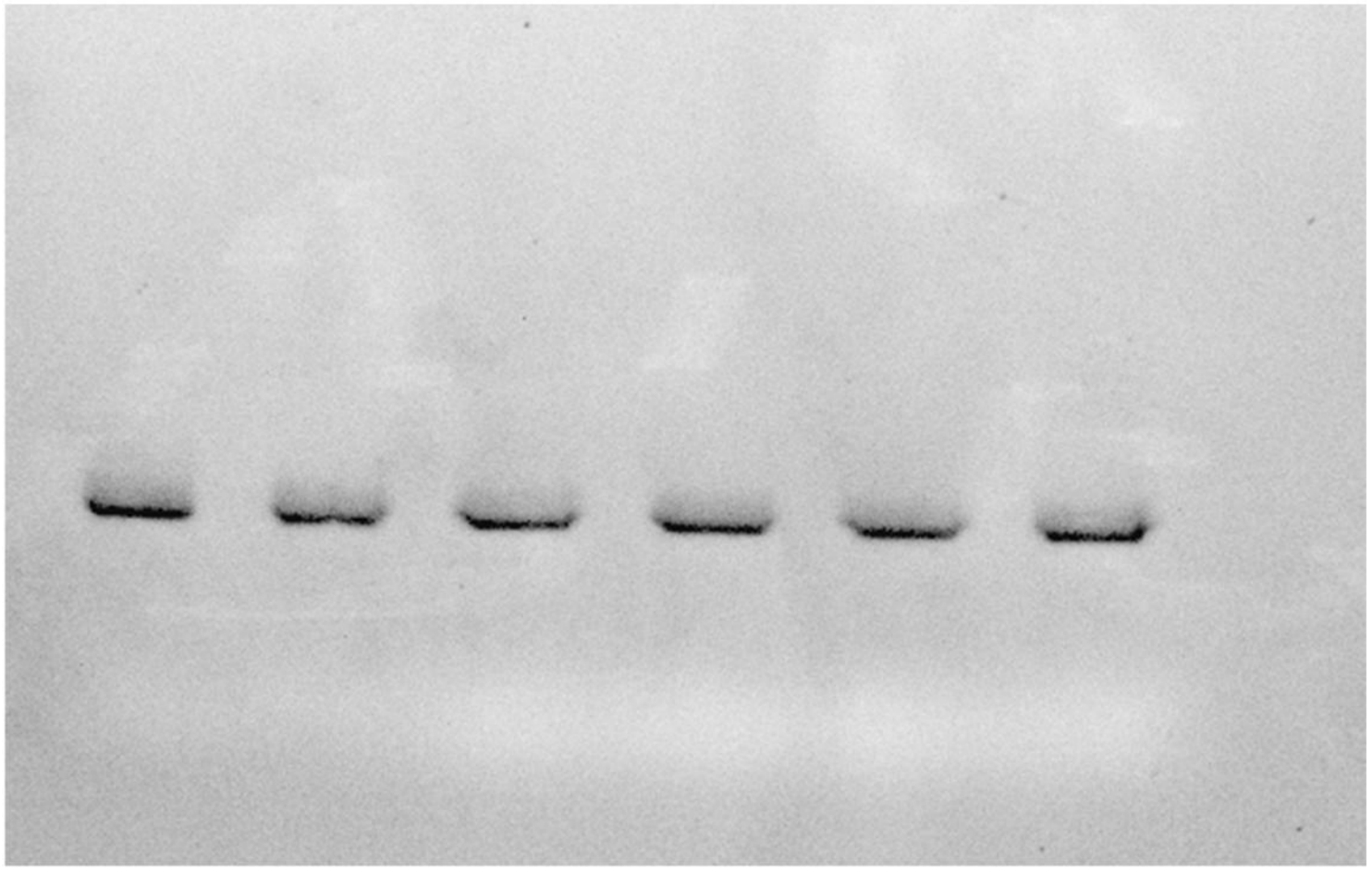

Supplementary_Fig_S7B_ raw_image

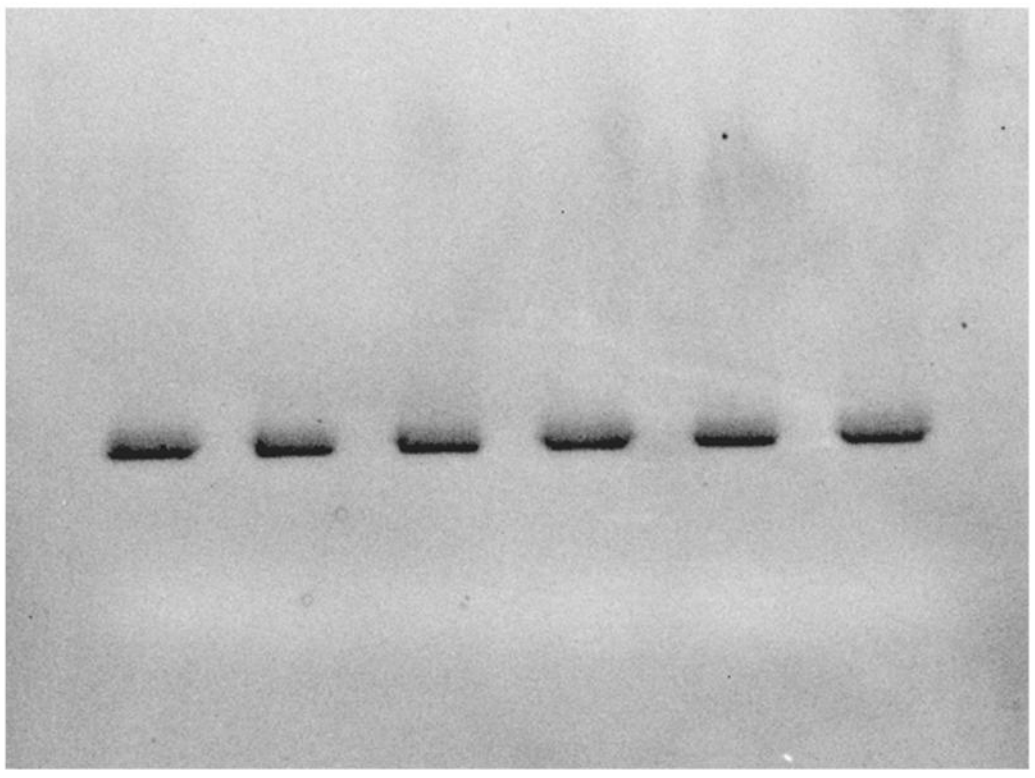

Supplementary_Fig_S8A_ raw_image

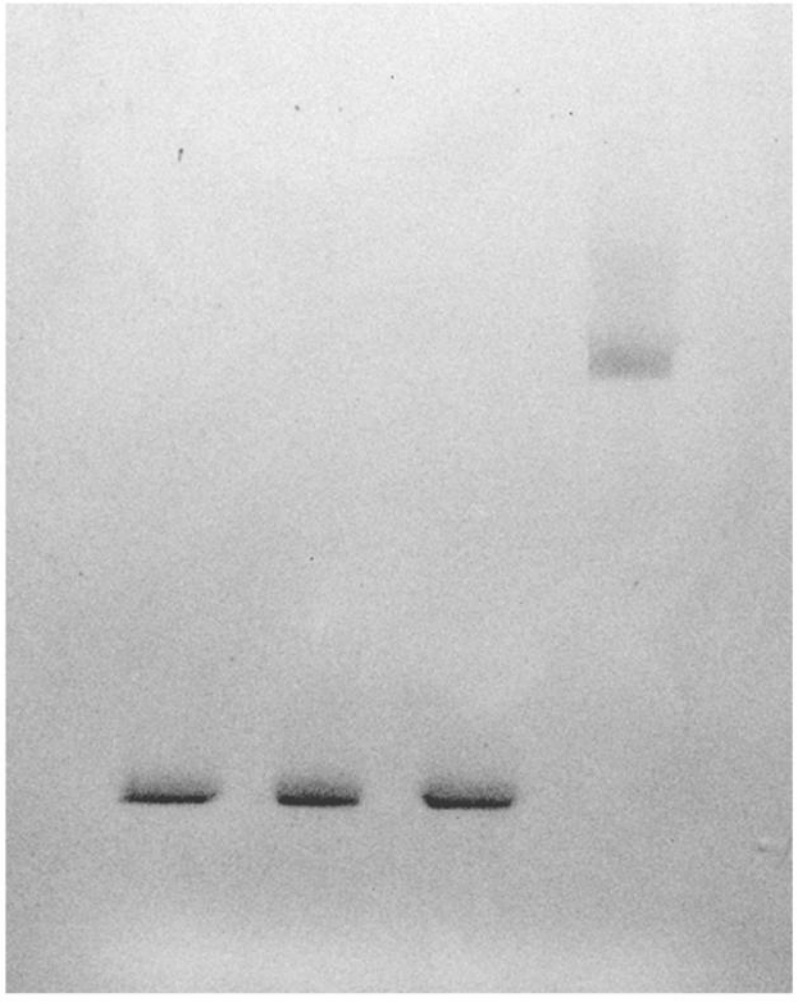

Supplementary_Fig_S8B_ raw_image

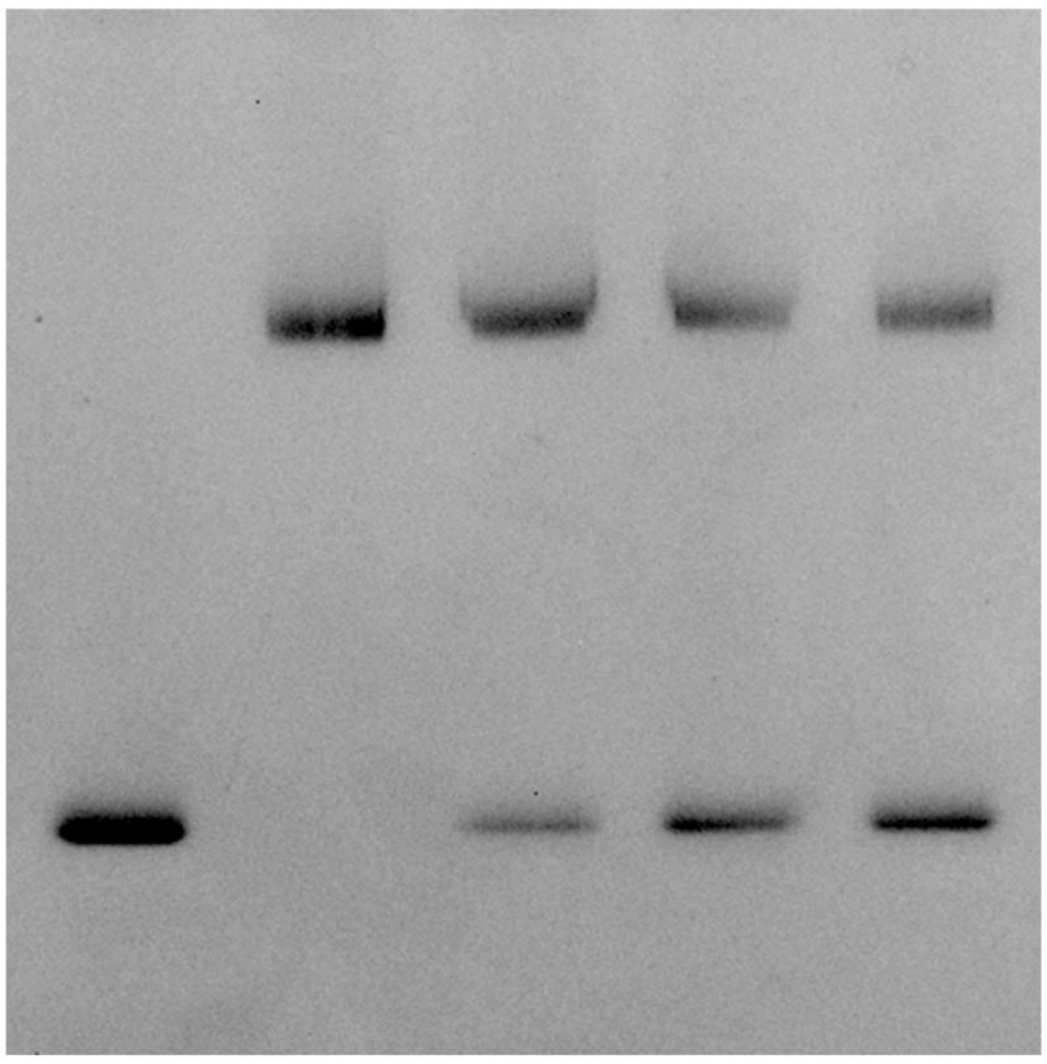

Supplementary_Fig_S8C_ raw_image

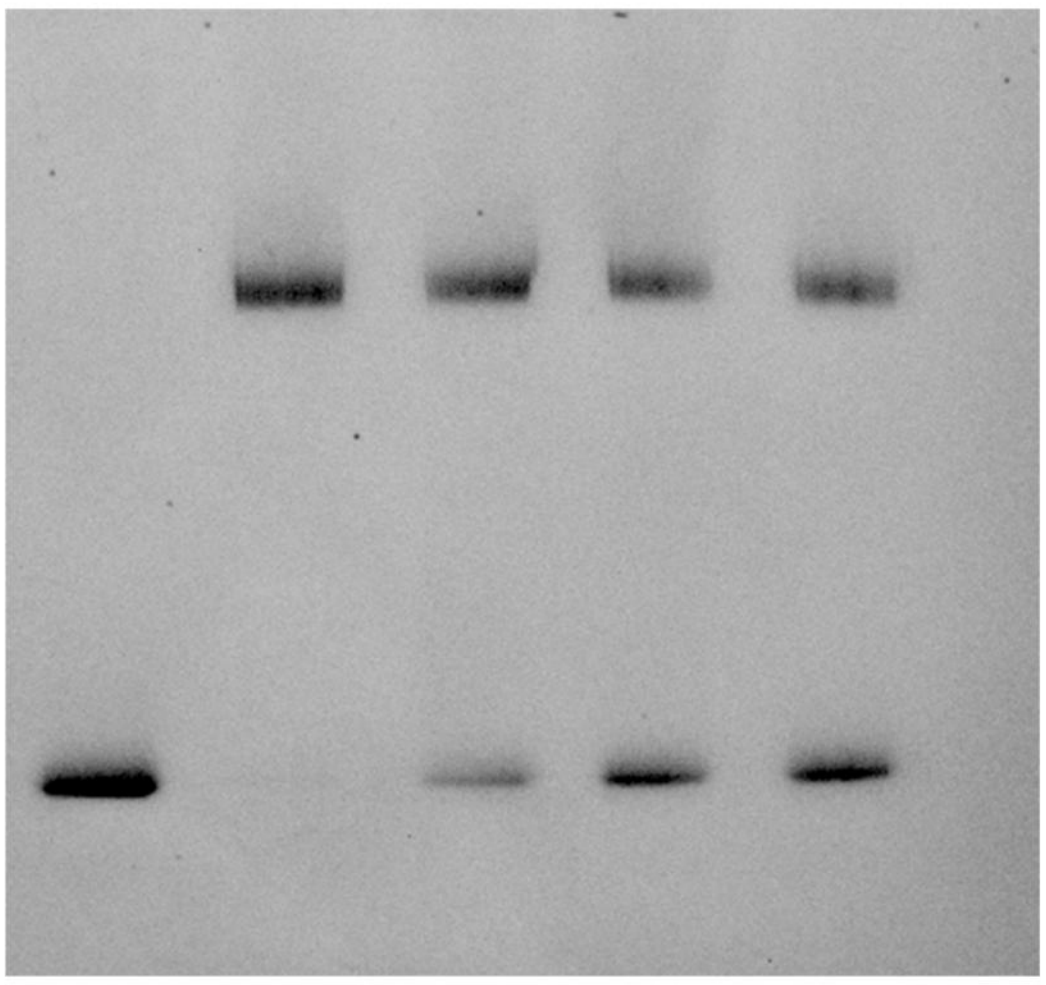

Supplementary_Fig_S8D raw_image

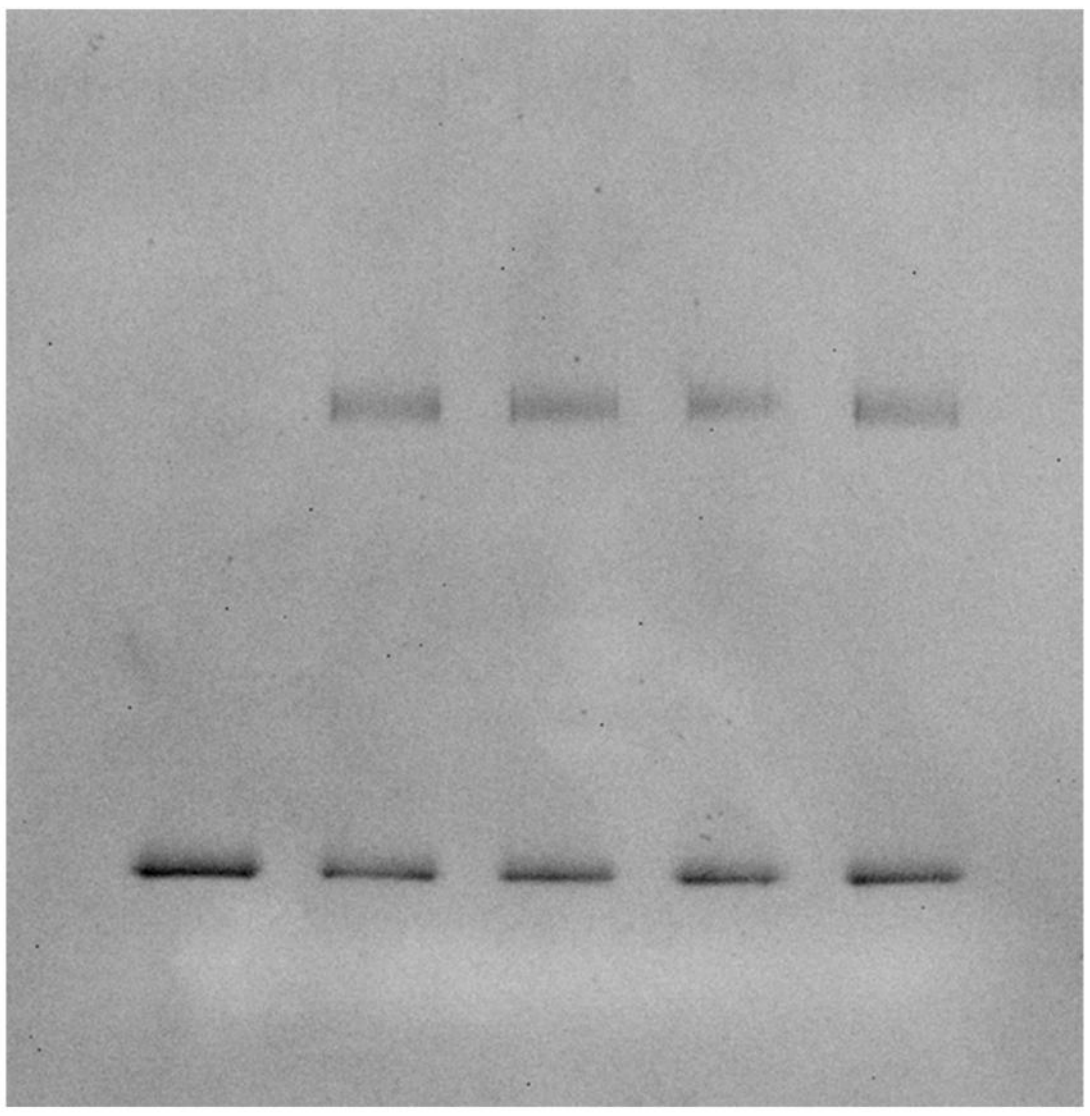

Supplement: Supplementary file 1 [file ppat.1014537.s014.pdf]
