# Supplementary material for: Cyclic di-GMP directly reprograms the multidrug resistance machinery via UspG-mediated sequestration of RamR in Klebsiella
Source: PLoS Pathog. 2026 Aug 24;22(8):e1014537. doi: 10.1371/journal.ppat.1014537 (PMC13524336; doi:10.1371/journal.ppat.1014537)
Supplement: Supplementary file 2 [file ppat.1014537.s012.docx]

**Table S4.** Bacterial strains and plasmids used in this study.

| **Bacterial strains/Plasmids Description** | | **Source** |
| --- | --- | --- |
| **Bacterial strains** | **Phenotype or characteristics** |  |
| *Klebsiella quasipneumoniae* ATCC 700603 | Wild-type strain of *Klebsiella quasipneumoniae* ATCC 700603 | Lab stock |
| *Klebsiella pneumoniae* ATCC 43816 | Wild-type strain of *Klebsiella pneumoniae* ATCC43816 | Lab stock |
| *Salmonella typhimurium* | Wild-type strain of *Salmonella typhimurium* | Lab stock |
| *Klebsiella oxytoca* | Wild-type strain of *Klebsiella oxytoca* | Lab stock |
| ATCC 700603 Δ*uspG* | Full deletion of *uspG* in ATCC 700603 | This study |
| ATCC 700603 Δ*ramA* | Full deletion of *ramA* in ATCC 700603 | This study |
| ATCC 43816 Δ*uspG* | Full deletion of *uspG* in ATCC 43816 | This study |
| BTH101 | *E. coli* adenylates cyclase deficient reporter strains | This study |
| *E. coil* DH5α | *supE44 lacU169*(*80lacZ M15*) *hsdR17 recA1 endA1 gyrA96 thi-1 relA1 pir* | This study |
| *E. coil* BL21(DE3) | *F-ompT hsdS* (*rB-mB-*) *dcm+ Tetr gal* (*DE3*) *endA* | This study |
| **Plasmids** | **Phenotype or characteristics** |  |
| pCasKP-apr | Bacterial expression of Cas9 nuclease and lambda-red system*,* Apr^r^ | Lab stock |
| pSGKP-smR | A sgRNA expression plasmid for genome editing in *K. pneumoniae,* Sm^r^ | Lab stock |
| pUC-smR | Cloning vector, Sm^r^ | This study |
| pUC-*wspR* | pUC containing *wspR* | This study |
| pUC-*rocR* | pUC containing *rocR* | This study |
| pUC-*uspG* | pUC containing *uspG* | This study |
| pUC-*ramA* | pUC containing *ramA* | This study |
| pUC-*uspG*^N39A^ | pUC containing *uspG*^N39A^ | This study |
| pUC-*uspG*^K116A^ | pUC containing *uspG*^K116A^ | This study |
| pUC-*uspG*^S126A^ | pUC containing *uspG*^S126A^ | This study |
| pME2-*wspR* | pME2-MCS carrying *wspR*, IPTG inducible *tac* promoter, Apr^r^ | This study |
| pME2-*ramR* | pME2-MCS carrying *ramR* which were expressed from IPTG inducible *tac* promoter, Apr^r^ | ^4^ |
| pME2-*uspG* | pME2-MCS carrying *uspG* which were expressed from IPTG inducible *tac* promoter, Apr^r^ | This study |
| pCold I | Expression vector, amp^r^ | This study |
| pET30-*uspG* | pET30a containing *uspG* | This study |
| pET30-*uspG*^N39A^ | pET30a containing *uspG*^N39A^ | This study |
| pET30-*uspG*^K116A^ | pET30a containing *uspG*^K116A^ | This study |
| pET30-*uspG*^S126A^ | pET30a containing *uspG*^S126A^ | This study |
| pCold-*ramR* | pCold I containing *ramR* | This study |
| pCold-*rimO* | pCold I containing *rimO* | This study |
| pCold-*sbcD* | pCold I containing *sbcD* | This study |
| pCold-*uspG*_Kp43816_ | pCold I containing *uspG*_Kp43816_ | This study |
| pCold-*uspG_K._ _oxytoca_* | pCold I containing *uspG_K._ _oxytoca_* | This study |
| pCold-*uspG_K. variicola_* | pCold I containing *uspG_K. variicola_* | This study |
| pCold-*uspG_E. coil_* | pCold I containing *uspG_E. coil_* | This study |
| pCold-*uspG_E. cloacae_* | pCold I containing *uspG_E. cloacae_* | This study |
| pCold-*uspG_S. typhimurium_* | pCold I containing *uspG_S. typhimurium_* | This study |
| pCold-*uspG_R. ornithinolytica_* | pCold I containing *uspG_R. ornithinolytica_* | This study |
| pUT18C | Bacterial Adenylate Cyclase Two-hybrid System Kit, Amp^r^ | This study |
| pKNT25 | Bacterial Adenylate Cyclase Two-hybrid System Kit, Kan^r^ | This study |
| *ramR*-T18 | pUT18C containing *ramR* | This study |
| *uspG*-T25 | pKNT25 containing *uspG* | This study |

Amp^r^, Kan^r^, Sm^r^，and Apr^r^ indicate resistance to ampicillin, kanamycin, streptomycin and apramycin, respectively.

**References**

1.	Zeng, Z., Liu, X., Yao, J., Guo, Y., Li, B., Li, Y., Jiao, N. & Wang, X. Cold adaptation regulated by cryptic prophage excision in *Shewanella oneidensis*. *The ISME J* **10**, 2787-2800 (2016).

2.	Jin, M., Jiang, Y., Sun, L., Yin, J., Fu, H., Wu, G. & Gao, H. Unique organizational and functional features of the cytochrome c maturation system in *Shewanella oneidensis*. *PLoS One* **8**, e75610 (2013).

3.	Kovach, M.E., Elzer, P.H., Hill, D.S., Robertson, G.T., Farris, M.A., Roop, R.M., 2nd & Peterson, K.M. Four new derivatives of the broad-host-range cloning vector pBBR1MCS, carrying different antibiotic-resistance cassettes. *Gene* **166**, 175-176 (1995).

4.	Liu, X., Lin, S., Liu, T., Zhou, Y., Wang, W., Yao, J., Guo, Y., Tang, K., Chen, R., Benedik, M.J. & Wang, X. Xenogeneic silencing relies on temperature-dependent phosphorylation of the host H-NS protein in *Shewanella*. *Nucleic Acids Res* **49**, 3427-3440 (2021).

5.	Shi, M., Wu, L., Xia, Y., Chen, H., Luo, Q., Sun, L. & Gao, H. Exoprotein production correlates with morphotype changes of nonmotile *Shewanella oneidensis* mutants. *J Bacteriol* **195**, 1463-1474 (2013).
