# Supplementary material for: Cyclic di-GMP directly reprograms the multidrug resistance machinery via UspG-mediated sequestration of RamR in Klebsiella
Source: PLoS Pathog. 2026 Aug 24;22(8):e1014537. doi: 10.1371/journal.ppat.1014537 (PMC13524336; doi:10.1371/journal.ppat.1014537)
Supplement: Supplementary file 3 [file ppat.1014537.s013.docx]

**Table S5.** Nucleotide sequences of primers used in this study. F indicates forward primer and R indicates reverse primer.

| **Primer /Purpose** | **Primer Sequence (listed 5’ to 3’)** |
| --- | --- |
| **Construction of in-frame insertion/deletion mutants** | |
| ATCC 700603 *uspG*-N20-F | TAGTGTTGGAGCCCAGCAGATGGG |
| ATCC 700603 *uspG*-N20-R | AAACCCCATCTGCTGGGCTCCAAC |
| ATCC 700603 *uspG*-up-F | AATTGACGCGTATTGGGATGGTACCTGTTGACGACGCCGGCCAG |
| ATCC 700603 *uspG*-up-R | ACGGAGGGGAGGCCCGCACTCCTTTTCC |
| ATCC 700603 *uspG*-down-F | AGTGCGGGCCTCCCCTCCGTCACGACGGC |
| ATCC 700603 *uspG*-down-R | CGACCTCGAGGGGGGGCCCGGTACCAATCTCCCTGTCTCAGGTGC |
| ATCC 700603 out-*uspG*-F | GGCTGGTCATTCACCGCA |
| ATCC 700603 out-*uspG*-R | CGCTGAGGCAGTCGAGAA |
| *ramA*-up-F | AATTGACGCGTATTGGGATGGTACCCGATTCCGGTTAACGACGA |
| *ramA*-up-R | GAACGCTAAACCAGGATGCTCTCTCCTCTGATACT |
| *ramA*-down-F | CAGAGGAGAGAGCATCCTGGTTTAGCGTTCGAGC |
| *ramA*-down-R | CGACCTCGAGGGGGGGCCCGGTACCGCTGGTGGGATATAGCACTT |
| *ramA*-N20-F | TAGTGATAGTATCAATCACCTGAG |
| *ramA*-N20-R | AAACCTCAGGTGATTGATACTATC |
| OuT-*ramA*-F | TGGCTCGCACCTGCGTTA |
| OuT-*ramA*-R | TGTACATCCTGCTGGCCA |
| ATCC 43816 *uspG*-N20-F | TAGTTTACCGGGATATGGGCATGG |
| ATCC 43816 *uspG*-N20-R | AAACCCATGCCCATATCCCGGTAA |
| ATCC 43816 *uspG*-up-F | AATTGACGCGTATTGGGATGGTACCCTCAGTATGATGGTAGGACCTT |
| ATCC 43816 *uspG*-up-R | ACCTGACGACCATTACCGGGATCGGCATAATGATAGTCTTATAC |
| ATCC 43816 *uspG*-down-F | GTATAAGACTATCATTATGCCGATCCCGGTAATGGTCGTCAGGT |
| ATCC 43816 *uspG*-down-R | CGACCTCGAGGGGGGGCCCGGTACCCTACTGCCCTCACTGCGATGGT |
| ATCC 43816 out-*uspG*-F | CTAATTCCGCTGCCTGAG |
| ATCC 43816 out-*uspG*-R | TGCTGGATACCGTGGATAT |
| **For EMSA** | |
| pI-F | CGCTACTTTTTTCCTCACGC |
| pI-R | CCCCTGCGGCGCCTTACCA |
| pII-F | TGGGCCAGTTTTCTGTTCGT |
| pII-R | GATAGTATCAATCACCTGAGCG |
| **For bacterial two-hybrid** | |
| *uspG*-T18-F | CACTGCAGGTCGACTATGTATAAAAAGATTTTATTGC |
| *uspG*-T18-R | AGTTATATCGATGAATCTGACCACCAGCACCGG |
| *uspG*-T25-F | TGCAGGTCGACTCTAATGTATAAAAAGATTTTATTGC |
| *uspG*-T25-R | CATTGAATTCGAGCTTCTGACCACCAGCACCGG |
| *ramR*-T18-F | CACTGCAGGTCGACTGTGAGGAAAAAAGTAGCGG |
| *ramR*-T18-R | AGTTATATCGATGAATTAGGCGTCCGCCTCATG |
| *ramR*-T25-F | TGCAGGTCGACTCTAGTGAGGAAAAAAGTAGCGG |
| *ramR*-T25-R | CATTGAATTCGAGCTTTAGGCGTCCGCCTCATG |
| **For in trans expression** | |
| pUC-*wspR*-F | CGACTCTAGAGGATCCCATGCACAACCCTCATGAGA |
| pUC-*wspR*-R | ACGACGGCCAGTGAATTCTCAGCCCGCCGGGGCCGG |
| pUC-*rocR*-F | CGACTCTAGAGGATCCCATGAATGATTTGAATGTTCTGG |
| pUC-*rocR*-R | ACGACGGCCAGTGAATTCTCAGGATCCGGAGCAATAGT |
| pUC-*uspG*-F | CGACTCTAGAGGATCCCATGTATAAAAAGATTTTATTGCCTGTT |
| pUC-*uspG*-R | ACGACGGCCAGTGAATTCTTATCTGACCACCAGCACC |
| pUC-*ramA*-F | CGACTCTAGAGGATCCCATGACGATTTCCGCTCAGGTG |
| pUC-*ramA*-R | ACGACGGCCAGTGAATTCTCAGTGGGCGCGACTGT |
| pUC-*uspG*^N39A^-F | CTTGCTCGCGGTCCTGCCGAACAGCAG |
| pUC-*uspG*^N39A^-R | GCAGGACCGCGAGCAAGGTGATCTCGCC |
| pUC-*uspG*^K116A^-F | CTCCCGCGCGCCGGGGATCTCCACCCAT |
| pUC-*uspG*^K116A^-R | TCCCCGGCGCGCGGGAGCCAATCACTA |
| pUC-*uspG*^S126A^-F | GCTGGGCGCGAACGCCGAGTCCATTTT |
| pUC-*uspG*^S126A^-R | CGGCGTTCGCGCCCAGCAGATGGGTGG |
| pME2-*wspR*-F | TCACACAGGAGAGAATTCATGCACAACCCTCATGAGA |
| pME2-*wspR*-R | GCCAAAACAGCCAAGCTTTCAGCCCGCCGGGGCCGG |
| pME2-*uspG*-F | TCACACAGGAGAGAATTCATGTATAAAAAGATTTTATTGCCTGTT |
| pME2-*uspG*-R | GCCAAAACAGCCAAGCTTTTATCTGACCACCAGCACC |
| pME2-*ramR*-F | TCACACAGGAGAGAATTCGTGAGGAAAAAAGTAGCGG |
| pME2-*ramR*-R | GCCAAAACAGCCAAGCTTTTAGGCGTCCGCCTCATG |
| **For recombinant protein expression** | |
| *ramR*-PC-F | ACCCTCGAGGGATCCGTGAGGAAAAAAGTAGCGGCT |
| *ramR*-PC-R | GACAAGCTTGAATTCTTAGGCGTCCGCCTCAT |
| *uspG*-P30-F | GCTGATATCGGATCCATGTATAAAAAGATTTTATTGCC |
| *uspG*-P30-R | ACGGAGCTCGAATTCTTATCTGACCACCAGCACCG |
| *uspG*^N39A^-P30-F | CTTGCTCGCGGTCCTGCCGAACAGCAG |
| *uspG*^N39A^-P30-R | GCAGGACCGCGAGCAAGGTGATCTCGCC |
| *uspG*^K116A^-P30-F | CTCCCGCGCGCCGGGGATCTCCACCCAT |
| *uspG*^K116A^-P30-R | TCCCCGGCGCGCGGGAGCCAATCACTA |
| *uspG*^S126A^-P30-F | GCTGGGCGCGAACGCCGAGTCCATTTT |
| *uspG*^S126A^-P30-R | CGGCGTTCGCGCCCAGCAGATGGGTGG |
| *uspG_K._* _oxytoca_-PC-F | ACCCTCGAGGGATCCATGTATAAAACAATCATTATGCCC |
| *uspG_K._* _oxytoca_-PC-R | GACAAGCTTGAATTCCTATCTCACCACCATCACCGGA |
| *uspG_S.typhimurium_*-PC-F | ACCCTCGAGGGATCCATGTATAAGACAATCATTATGCC |
| *uspG_S.typhimurium_*-PC-R | GACAAGCTTGAATTCTTAACGCACAACCAATACGGG |
| *uspG_E. coil_*-PC-F | ACCCTCGAGGGATCCATGTATAAGACAATCATTATGCC |
| *uspG_E. coil_*-PC-R | GACAAGCTTGAATTCTTAACGCACAACCAGCAC |
| *uspG*_Kp43816_-PC-F | ACCCTCGAGGGATCCATGTATAAAAAGATTTTATTGCCTG |
| *uspG*_Kp43816_-PC-R | GACAAGCTTGAATTCTTATCTGACCACCAGCACC |
| *uspG_K. variicola_*-PC-F | ACCCTCGAGGGATCCATGTATAAAAAAATTTTATTGCCT |
| *uspG_K. variicola_*-PC-R | GACAAGCTTGAATTCTTATCTGACCACCAGCACC |
| *uspG_R. ornithinolytica_*-PC-F | ACCCTCGAGGGATCCATGTACAAAAAAATTTTGCTA |
| *uspG_R. ornithinolytica_*-PC-R | GACAAGCTTGAATTCCTATCTGACCACCAGAACCGG |
| *uspG_E. cloacae_*-PC-F | ACCCTCGAGGGATCCATGTATAAAAAGATTTTGATGCC |
| *uspG_E. cloacae_*-PC-R | GACAAGCTTGAATTCTCAGCGCACGACCAGGAC |
| **For qPCR** | |
| *ramA*-F | CGTCGAGTGGATTGATGAC |
| *ramA*-R | ATCGGTGTCACGCAGATC |
| *romA*-F | ATCCTGATCGACCCGGT |
| *romA*-R | TGGCGTAATCCAGATGGT |
| *lpxO*-F | GATTCTCACCGCTGGTAT |
| *lpxO*-R | GCGCCATCGCGACCCCTA |
| *lpxL*-F | AGACCAGCGGCGTAAAC |
| *lpxL*-R | GGTCTGCAGCCAGTCAATC |
| *acrA*-F | CCTTCGGTCACTGCGGA |
| *acrA*-R | CAAGACTACGATACCGCC |
| *acrB*-F | CGTCATCTCATCCAGCAC |
| *acrB*-R | CGCTATCTGGTGCTGTATC |
| *tolC*-F | CCATTACTGCCTCAGCTCG |
| *tolC*-R | CGTGACATCCTGAATGCC |
| *yhcN*-B-F | GCGCAATGATCATCGCA |
| *yhcN*-B-R | TAGCGGCCAGTTCCTGAC |
| *ariR*-F | TGCATCAGCAACCCGAT |
| *ariR*-R | GTTCAGGTGACCCTCGGAC |
| Hp09-F | ATGGCTTGGGAACCAGCTGAAT |
| Hp09-R | GGCTTAGTTGCTCATCATCGCG |
| *mrkB*-F | GGCGGAATTTCCAACAGAT |
| *mrkB*-R | TGCCACACCGGATACCA |
| *AVR78_RS21270*-F | CCGTTGCCCAGGCTGATA |
| *AVR78_RS21270*-R | TTGGCTGCGCCGAAGGTA |
| RNA16S-F | ATGACCAGCCACACTGGAAC |
| RNA16S-R | CTTCCTCCCCGCTGAAAGTA |
| ATCC 700603 *uspG*-F | CATTTCAGCATTGACCCCTCAC |
| ATCC 700603 *uspG*-R | GGATATGGGCATGACGGATGAC |
